# *Taxator-tk*: precise taxonomic assignment of metagenomes by fast approximation of evolutionary neighborhoods

**DOI:** 10.1093/bioinformatics/btu745

**Published:** 2014-11-10

**Authors:** J. Dröge, I. Gregor, A. C. McHardy

**Affiliations:** ^1^Department for Algorithmic Bioinformatics, Heinrich Heine University, Universitätsstraße 1, 40225 Düsseldorf, Germany, ^2^Max-Planck Research Group for Computational Genomics and Epidemiology, Max-Planck Institute for Informatics, University Campus E1 4, 66123 Saarbrücken, Germany and ^3^Computational Biology of Infection Research, Helmholtz Centre for Infection Research, Inhoffenstraße 7, 38124 Braunschweig, Germany

## Abstract

**Motivation:** Metagenomics characterizes microbial communities by random shotgun sequencing of DNA isolated directly from an environment of interest. An essential step in computational metagenome analysis is taxonomic sequence assignment, which allows identifying the sequenced community members and reconstructing taxonomic bins with sequence data for the individual taxa. For the massive datasets generated by next-generation sequencing technologies, this cannot be performed with *de-novo* phylogenetic inference methods. We describe an algorithm and the accompanying software, *taxator-tk*, which performs taxonomic sequence assignment by fast approximate determination of evolutionary neighbors from sequence similarities.

**Results:** *Taxator-tk* was precise in its taxonomic assignment across all ranks and taxa for a range of evolutionary distances and for short as well as for long sequences. In addition to the taxonomic binning of metagenomes, it is well suited for profiling microbial communities from metagenome samples because it identifies bacterial, archaeal and eukaryotic community members without being affected by varying primer binding strengths, as in marker gene amplification, or copy number variations of marker genes across different taxa. *Taxator-tk* has an efficient, parallelized implementation that allows the assignment of 6 Gb of sequence data per day on a standard multiprocessor system with 10 CPU cores and microbial *RefSeq* as the genomic reference data.

**Availability and implementation**: *Taxator-tk* source and binary program files are publicly available at http://algbio.cs.uni-duesseldorf.de/software/.

**Contact:** Alice.McHardy@uni-duesseldorf.de

**Supplementary information:** Supplementary data are available at *Bioinformatics* online.

## 1 Introduction

Metagenomics allows us to study microbial communities from natural environments without the need to obtain pure cultures of the individual member species (Hugenholtz, 2002; Riesenfeld *et al*., 2004). The shotgun sequencing of microbial community DNA with current techniques generates reads that range from less than 100 to several thousand nucleotides (Dröge and McHardy, 2012; Klumpp *et al*., 2012). By computational analyses of metagenome sequence samples, we can estimate the abundances of different taxa for the sampled communities, known as taxonomic profiling, characterize their functional and metabolic potential based on the predicted proteins and resolve the contributions of individual taxa to the latter by reconstructing ‘bins’ of unassembled or assembled sequences that originate from the same taxon.

A taxonomic profile of a microbial community can be inferred by either targeted amplification and sequencing of taxonomic marker genes or from metagenome shotgun datasets (Lindner and Renard, 2013; Sunagawa *et al.*, 2013; Silva *et al.*, 2014). Most metagenome profiling methods classify reads based on predefined taxon-specific (Segata *et al.*, 2012) or ‘universal’ marker genes (Darling *et al.*, 2014), or directly estimate a taxonomic profile for the underlying microbial community from their *k*-mer composition (Koslicki *et al.*, 2013). Frequently used phylogenetic placement programs within such frameworks are *pplacer* (Matsen *et al*., 2010) or *EPA/RAxML* (Berger *et al*., 2011), which both operate in a probabilistic framework to place a query gene sequence in a pre-computed reference phylogeny of a particular gene family. If this gene tree is an approximate representation of the respective species tree—or reference taxonomy—this can be used to assign a taxon identifier (ID) to the query sequence (Stark *et al*., 2010; Matsen and Gallagher, 2012). Taxon abundances are then derived from the individual read counts or gene frequencies within each taxonomic group.

Binning methods place the sequences of a shotgun metagenome sample into bins representing the different taxa of the sampled microbial community. If a bin represents a low-ranking taxon, such as species, then the set of reads or contigs of an individual taxonomic bin serves as a draft-genome reconstruction for a community member (Pope *et al.*, 2011). Binning methods are either based on clustering or classification. Clustering methods group sequences into bins without consideration of external reference sequences or taxonomic information. Instead, bins are inferred based on similarities in GC content, oligomer frequencies, the abundance of genes or contig coverage within one or multiple samples (Baran and Halperin, 2012; Albertsen *et al.,* 2013; Carr *et al*., 2013; Alneberg *et al*., 2014), or by using a combination of these (Iverson *et al.*, 2012). This allows draft genome recovery from deep lineages for sequences of sufficient length. Taxonomic binning, like profiling, uses the resemblance of a sequence to known taxa in either global sequence composition or local sequence similarity to assign a taxon ID. For the human gut microbiome, extensive genome sequencing of isolate cultures allowed species-level taxonomic binning for a substantial portion (∼40%) of a metagenome sample (Schloissnig *et al*., 2013) by mapping the reads to isolate genome sequences, which exist for many abundant species (Sunagawa *et al.*, 2013). However, this procedure is not suitable for environments in which most species are from deep-branching lineages without available reference genome sequences. Taxonomic binning of these requires more sophisticated similarity-based or composition-based taxonomic assignment methods (McHardy *et al*., 2007; Brady and Salzberg, 2011; Huson *et al*., 2011). Taxonomic binning by sequence composition also allows draft genome recovery from deep-branching lineages, based on limited amounts of sequences for the individual taxa (McHardy *et al*., 2007). Composition-based programs achieve linear classification times regarding metagenome sample size, whereas similarity-based binning methods require considerably more computational resources for sequence similarity searches in large reference sequence collections. Programs with a focus on processing large amounts of raw sequencing reads, such as *Kraken* (Wood and Salzberg, 2014), implement the fastest search strategies. Similarity-based programs are more accurate for the assignment of sequences shorter than 1 kb (Patil *et al*., 2011).

A common aim in taxonomic profiling and taxonomic binning is the identification of known taxa from a sample. A taxonomic profiler estimates a taxonomic abundance profile for the entire sample, which can be inferred by analyzing a smaller number of marker genes, though one needs to account for variations in gene copy numbers for taxon-specific markers (Lindner and Renard, 2013). Taxonomic binning assigns taxon IDs to a large portion of the sample sequences for subsequent functional and metabolic analyses of individual taxon bins. In addition, one can generate a taxonomic profile by quantifying the assigned reads, based on read counts or coverage for each individual bin.

From a methodological standpoint, the differences between the phylogenetic-placement-based methods for profiling and alignment-score-based methods for taxonomic binning and profiling, such as *MEGAN* (Huson *et al*., 2011), *CARMA3* (Gerlach and Stoye, 2011) or *SOrT-ITEMS* (Monzoorul Haque *et al*., 2009) are that the latter lack a well-motivated evolutionary framework. However, they have the advantages of being computationally lightweight and applicable to arbitrary genes, which is a necessity for taxonomic binning. Phylogenetic-placement-based methods cannot currently be used for binning, because the *de-novo* inference of trees for gene families on a metagenome-wide scale is computationally too demanding, particularly for next-generation sequencing (NGS) data.

Our taxator toolkit (*taxator-tk*) is a software package for the taxonomic sequence assignment in shotgun metagenomics with applications to both profiling and binning. Conceptually, it lies between sequence similarity-based programs which use local sequence alignment scores and those using trees. *Taxator-tk* extends the alignment score-based approach by approximating phylogenetic gene trees and thereby provides more accurate taxonomic assignments, without assuming universal, rank or clade-specific gene conservation levels as parameters. We improve in terms of applicability to large datasets compared with phylogenetic methods by assigning genomic sequences without the computationally demanding steps of *de-novo* multiple sequence alignment (MSA) and tree inference. *Taxator-tk* determines a subset of homologs, which represent the approximate evolutionary neighbors for a query sequence, by a linear number of pairwise sequence comparisons with regard to the number of considered homologs and then assigns a taxon ID using a reference taxonomy based on the taxonomic IDs of these neighbors. We have furthermore reduced the run-time by limiting the analysis to distinct homology-supported regions of the query sequence, which we termed query segmentation. Our open-source (GPLv3) software can be applied to arbitrary nucleotide sequences, such as reads, contigs, scaffolds and complete genomes sequences. It can be downloaded from http://algbio.cs.uni-duesseldorf.de/software/.

## 2 Methods

### 2.1 *Taxator-tk*’s workflow for taxonomic assignment

**Fig. 1.**
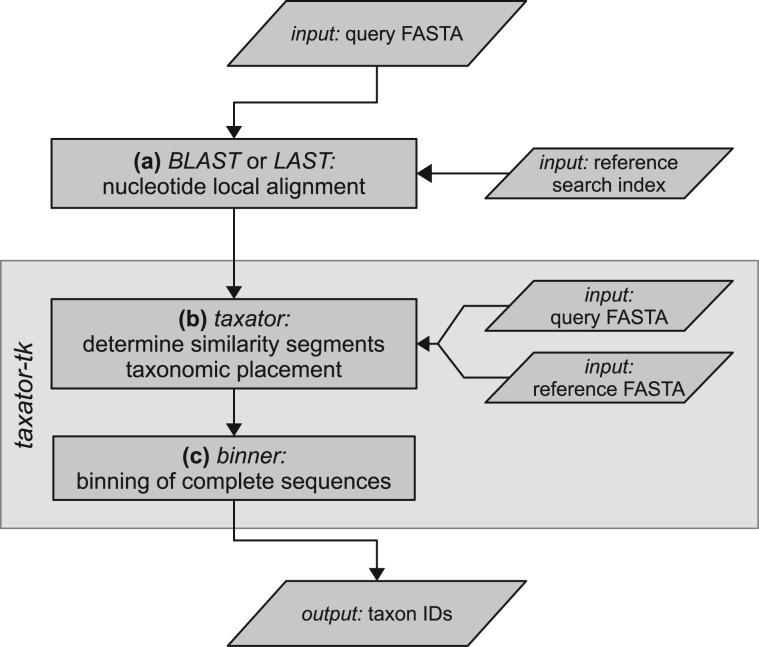
Workflow diagram for the taxonomic assignment of a nucleotide query sequence with *taxator-tk*. Taxonomic assignment with *taxator-tk* includes three steps. (**a**) Homology search for query sequence in reference collection using a nucleotide local alignment program. (**b**) Program *taxator* splits the query into distinct segments and determines a taxon ID for each using the corresponding homologs. (**c**) Program *binner* determines a taxon ID for the entire query based on the taxonomic assignments of the individual segments

The workflow for the taxonomic assignment of a nucleotide query sequence comprises three stages (Fig. 1a–c). The first stage uses a local sequence aligner to identify similar regions from a reference sequence collection, such as microbial *RefSeq* (*mRefSeq*) (Sayers *et al*., 2009). The implemented workflows currently use *BLAST+* (Camacho *et al*., 2009) version 2.2.28+ using any of the *blastn*, *megablast* or *tblastx* algorithms and nucleotide *LAST* (Frith *et al*., 2010) version 320. Other aligners can be used via conversion to a TAB-separated format, if found to be more appropriate. We discuss our choice of the aligner in the Supplementary Material (‘IX. Sequence homology search via local alignment’). At the beginning of the *taxator* algorithm in Stage 2, overlapping regions on the query, each defined by local alignment to a nucleotide reference sequence, are merged into larger subsequences called segments (Supplementary Fig. S1). These query segments are flanked by regions without similarity to any reference data (Supplementary Fig. S2) and are not considered further. This step reduces the overall number of positions in the following alignment computations and improves the taxonomic assignment of queries that have undergone genome rearrangements, resulting in a different order of these segments. The reference sequence regions corresponding to the local alignments are extended at both sides by the missing number of nucleotides to match to the corresponding query segment with respect to its length and we refer to these as reference segments. Each independent set of homologous segments is the input to the core algorithm in the program *taxator* in Stage 2 (Fig. 1b), which calculates independent taxon IDs for every corresponding query segment.

In the third stage (Fig. 1c), multiple segments belonging to the same query are considered and their IDs are combined in the program *binner*, to derive a consensus taxon ID. The corresponding algorithm weights the individual segment assignments by the number of identical bases to the closest reference sequence and assigns to the entire query the taxon ID supported by the majority (default = 70% identical bases) of weighted assignments with a minimum number of identical bases (default = 50 bp) (Supplementary Material, ‘II. Consensus binning algorithm’). *Binner* has the optional parameters minimum sequence identity and minimum sample abundance, but these were not applied in our analysis. If the taxonomic information is limited or contradictory, *taxator* and *binner* assign identifiers to higher ranking taxa in a conservative fashion to obtain the most reliable taxonomic assignments.

### 2.2 The taxonomic assignment algorithm (*taxator*)

**Fig. 2.**
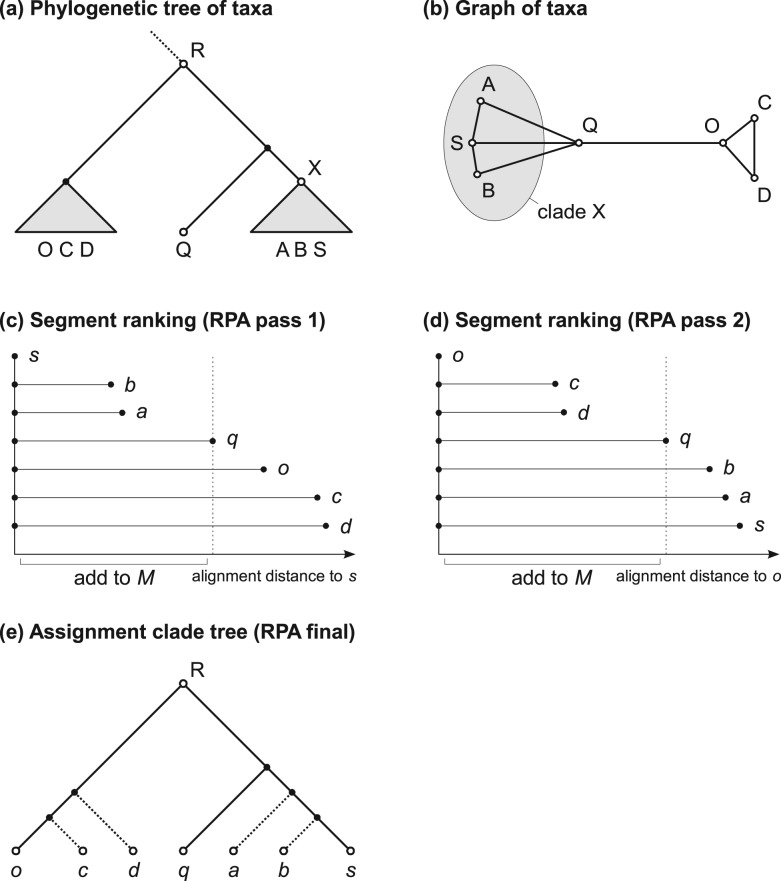
Algorithm for taxonomic labeling of query segments (realignment placement algorithm/RPA). The RPA assigns a taxon ID to a query segment *q*. (**a**) Species reference tree with query taxon Q and reference taxa A, B, C, D, O and S. This will be approximated by the segment phylogenetic tree for the query segment and homologous segments of reference taxa. (**b**) Approximate graph representing pairwise distances between the taxa. The subgraph for clade X is highlighted. (**c** and **d**) The two alignment passes which add segment taxa to an (empty) set *M*. Segment *s* is the segment with the smallest local alignment score (distance) to *q* in the initial similarity search. (**c**) First, all segments are aligned to segment *s*. The resulting distances are ordered and the taxa with equal or smaller distances than *distance(s,q)* are added to *M*. The outgroup segment, here *o*, is the next most similar segment to *s* after *q,* with *distance(o,s) > distance(s,q).* (**d**) All segments are aligned to *o*. From the ranked distances, taxa with distances smaller than *distance(o,q)* are also added to *M*. Thus, *M* includes all the nearest evolutionary neighbors for the query segment *q* (the taxa corresponding to segments *a*, *b*, *c*, *d*, *o* and *s*)*.* The taxon ID then assigned to *q* is the lowest common ancestor in the reference species tree (reference taxonomy) of these taxa in *M*. (**e**) Partially resolved segment subtree at node R that is implied by distances obtained in (c) and (d), where the exact position of some segments (*a*, *b*, *c* and *d*; dashed branches) is left unresolved by the RPA

The input to the algorithm is a segment *q* of the original query sequence from an (unknown) taxon Q and a set of homologous segments with known taxon IDs. The term ‘segment’ refers to a gap-less subsequence of either the query or a reference sequence. Given that for the set of homologs we know the correct underlying species tree of taxa (Fig. 2a), we can see that for our query taxon Q, the closest evolutionary neighbors would be A, B and S. If we simply assign X, the parental taxon of A, B and S, as a taxon identifier, this would be inaccurate, as A, B and S are more closely related to each other than to Q. Instead, the correct taxonomic assignment would be a parent of X and Q, and of at least one additional outgroup taxon (O) in the reference tree, such that Q also becomes a descendant of the identified parent (R in Fig. 2a). If we therefore identify the taxa A, B, S and O in the reference tree, we can determine the taxon ID of R as the lowest common ancestor (LCA) of these taxa and assign it to Q (and *q*).

Assuming that the underlying segment tree for a set of homologs is similar to the species tree, a natural procedure to identify the segments corresponding to the leaf taxa within R among the homologs would be to construct a MSA for the segment and a phylogenetic tree with a corresponding subtree as in Figure 2a. However, the computational effort for this approach is superlinear with respect to the number of homologs being compared and substantial for all the query segments in a large sample, even using fast techniques for MSA construction and tree inference. The *taxator* algorithm attempts to identify these segments with a linear number of pairwise segment comparisons. Let us consider an undirected graph in which nodes represent the segments (tree leaves) and edge lengths the evolutionary distances between pairs of segments within the underlying tree (Fig. 2b). In this graph, a monophyletic group in the species tree is a subgraph. For all pairs of subgraph nodes, the following inequality is true, given that the segments have evolved with a constant rate of evolution (i.e. the segment tree is ultrametric): The distance between any two subgraph nodes is smaller than that to any other node outside the subgraph. The relationship becomes clearer when thinking of the evolutionary distance between two nodes as the divergence time from their most recent ancestor. Members of a monophyletic group derive from a single common ancestor and thus there is a maximum distance for all possible pairs. If one member’s distance to an outside node is smaller than this maximum, both must share a more recent common ancestor and the corresponding group is not monophyletic by definition. The stated inequality can be used to augment an incomplete group or corresponding subgraph iteratively by taking an internal distance, ideally close to the maximum, as a threshold and adding outside nodes to the group which have a smaller distance to some internal node.

In this manner, *taxator* searches for the leaf node taxa of clade R among all segments based on a linear number of sequence comparisons between the input segments and adds them to an empty working set *M:*

0. A ranking by alignment scores from the input local alignments is used at the beginning to identify the reference segment *s* that is most similar to the query *q*.

The working set *M* is then augmented in two passes:

1. In the first pass, all segments are aligned to *s* using fast nucleotide alignment and the edit distance. These scores in the following serve to approximate the evolutionary distances in the underlying segment phylogeny. All segment taxa with a distance less than or equal to the threshold *distance(s,q)* are added to *M* (Fig. 2c).
2. The outgroup segment *o* is determined as the first segment for which *distance(s,o)* is larger than *distance(s,q)*. In the second pass, all segments are then aligned to *o* and segment taxa with distances smaller than or equal to *distance(o,q)* are added to *M* as well (Fig. 2d).

This procedure requires approximately 2*n* alignments, where *n* is the number of reference segments.

3. The resulting set *M* of taxa (implicit in the partially resolved tree in Fig. 2e) is used to determine the taxon ID for *q*, corresponding to the LCA of these taxa in a reference taxonomy, such as the NCBI taxonomy.

If no outgroup could be determined or if *M* is so diverse that the LCA corresponds to the taxonomy root, *q* is left unassigned. The algorithm requires at least two homologous segments (*s* and *o*) to determine a meaningful taxon ID. The taxa in *M* become more diverse if the alignment scores are inaccurate ultrametric distance estimates, if the species subtree’s topology deviates from the respective part of the taxonomy or if the gene tree’s topology deviates from the species tree, for instance due to varying rates of evolution or the inclusion of non-homologous segments in the analysis. The robustness of the algorithm in avoiding incorrect assignments under these circumstances relies on the number of taxa in *M* and the subsequent LCA operation. Further details relating to the robustness of the implementation are given in the Supplementary Materials, ‘I. Taxonomic assignment of sequence segments’.

### 2.3 Evaluation procedures

Before evaluating any method, we removed the smallest predicted bins (1%) as likely errors. We used the macro-precision and macro-recall as measures of assignment performance (Supplementary Materials, ‘Performance measures’). The macro-precision specifies the fraction of correct assignments per predicted bin (precision), averaged over all such bins, whereas the macro-recall measures the fraction of correctly recovered sequence data per truly existing bin (recall), averaged over all such bins. To account for strong differences in bin size, we also pooled the species, genus and family assignments, and reported the overall precision for these ranks as the total fraction of correct assignments. We tested the assignment performance of different methods using three simulated short read datasets, simulated 16S rRNA data, three simulated metagenome contig datasets and using assembled cow rumen metagenome contigs. For every simulated dataset, we performed seven cross-validation experiments (Supplementary Materials, ‘VII. Cross-validation’). In each experiment, we simulated a specific minimum taxonomic distance between a query sequence and the reference sequences. For the first experiment, all reference data, including the species genome data from which the query had been sampled, were made available to the method for assigning a single query sequence as an idealized test case. In the other six scenarios, all reference data belonging to the species, genus, family, order, class or phylum of the query sequence, respectively, were made inaccessible for the method in leave-one-taxon-out cross-validation experiments. We summarized the sequence assignments from these experiments to characterize a method’s assignment performance across the entire range of taxonomic distances. For evaluation with the cow rumen metagenome sample, for which no true taxonomic labels were known, we divided the assembled contigs into multiple sequence ‘chunks’ and characterized the consistency of taxonomic assignments for chunks originating from same contig (Supplementary Material, ‘VIII. Consistency analysis’).

## 3 Results

### 3.1 Evaluation with unassembled data

We first evaluated the performance of *taxator-tk* for classification of the most widely used taxonomic marker in bacterial diversity studies—the 16S rRNA gene (Supplementary Fig. S3). This served as a proof of concept, as *taxator-tk* classifies arbitrary sequence regions including taxonomic marker genes. We did not expect it to perform better than sophisticated phylogenetic models for this task, but wanted to confirm a satisfactory performance. The macro-precision for the taxonomic assignment of 7176 16S rRNA genes (Supplementary Fig. S4) was constantly above 92% (Supplementary Fig. S3a) in the combined cross-validation (Methods), using the whole-genome reference sequences in *mRefSeq47* (Supplementary Fig. S5), not just the 16S genes. More precisely, the average error rate per bin (one minus precision) was 7.4% at the species level and 4.6% at the order level.

Next, we simulated 100 000 reads at 100, 500 and 1000 bp by subsampling randomly from 1729 species in *mRefSeq47* and evaluated *taxator-tk* with these three datasets using the (combined) cross-validation experiments. The performance was very similar for the different fragment sizes (Supplementary Figs S6–S8a). Overall, *taxator-tk* showed high precision in simulated read assignment: the macro-precision for all short read lengths remained above 74% and was 82–99% for the genus to kingdom ranks, about 10% lower on average than for the 16S data. This was still good for the assignment of short sequence fragments from arbitrary genomic regions compared with a marker gene. At genus level, the macro-recall was 19–23% (∼33% genera recovered) if genome sequences of the same species as the query sequence were provided in the reference (Supplementary Figs S6–S8b) and as low as 5–7% (∼16% genera recovered) otherwise (Supplementary Figs S6–S8c). The macro-recall depends on the availability of related reference data at the respective ranks. It decreases when removing reference data for cross-validation. For example, if all reference data at genus level are removed, then no correct assignments to the genus rank are possible. For lower taxonomic ranks, the macro-recall was also low due to the large number of sample taxa and their uneven representation caused by the taxonomic bias toward a few abundant phyla in *mRefSeq47*. The longer reads had a slightly higher macro-recall than the shorter ones. Since longer sequences yield better recall and because overlapping reads contain redundant information, leading to more alignment computations, we recommend applying *taxator-tk* to (partially) assembled data. For longer query sequences, we were more likely to find segments for processing and therefore to assign a larger portion of the sample.

### 3.2 Evaluation with simulated metagenome contigs

For our tests on three simulated contig samples, we compared *taxator-tk* to *CARMA3* and *MEGAN4/5* using the same taxonomy and the same nucleotide alignments against *mRefSeq54* (Supplementary Fig. S9). Additionally, we applied these three methods to two datasets using protein-level alignments which we inferred using *BLAST+/tblastx*. When doing so, we used the programs recommended parameter settings (Supplementary Material, ‘X. Program parameters and versions’) and cross-validation, as before (Supplementary Material, ‘V. Cross-validation’).

We created a simulated NGS metagenome dataset (simArt49e, composition in Supplementary Fig. S10) for our evaluation. This sample includes 49 equally abundant species (51 strains) and was created by Illumina paired read simulation with *pIRS* (Hu *et al*., 2012), followed by *SOAPdenovo* version 1.05 (Luo *et al.,* 2012) assembly. Around 160 Mb or 267 178 contigs remained after removal of 0.03% chimeric sequences. In the combined cross-validation with this dataset (Supplementary Figs S11–S13a), *taxator-tk* produced substantially fewer errors: sequence assignments to species, genus and family were 91% correct for *taxator-tk*, compared with 52% for *CARMA3* and 59% for *MEGAN4*. Accordingly, *taxator-tk* showed the highest macro-precision of all methods, e.g. 61% at the species level, compared with 3% (*CARMA3*) and 5% (*MEGAN4*). The low macro-precision observed for *CARMA3* and *MEGAN4* is largely due to the prediction of many small bins with many false assignments (Supplementary Material, ‘V. Performance measures’). The majority of assignments were to bacteria, archaea or undetermined in the case of *CARMA3*, because we restricted the availability of similar reference sequences in each of the individual cross-validations, which we then jointly assessed.

**Fig. 3.**
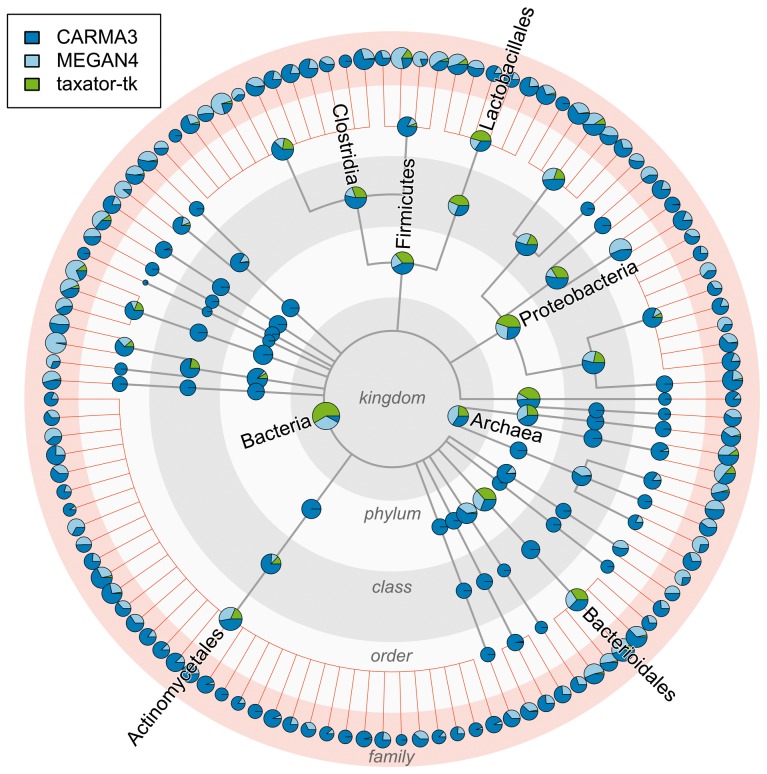
Comparison of three classifiers for a novel-family simulation using a simulated metagenome sample (simArt49e) with 49 species. *CARMA3*, *MEGAN4* and *taxator-tk*: the outer ring with red background shading shows family-level assignments for all orders included in the simulated dataset. These are all false in the chosen evaluation scenario, as no data from the families of the query sequences were included in the reference collection in the leave-one-taxon-out cross-validation experiments. Clearly, taxator-tk had the fewest assignments at family level, demonstrating its high precision in assignments. Assignments at inner rings, gray background shading, can be correct in principle, demonstrating at which taxonomic ranks the different methods tend to make their assignments, with taxator-tk tending toward producing higher ranking assignments, as a trade-off for the high precision

When only the sequences from the corresponding species and genus were removed from the reference (new genus scenario, Supplementary Figs S11–S13d), *taxator-tk* was also the most precise, though it had a lower recall than the other methods (*taxator-tk*: 56% family macro-precision, 60% overall precision for species to family, 10% family macro-recall; *CARMA3*: 13%, 27% and 20%; *MEGAN4*: 22%, 27% and 31%). Differences in assignment precision were also evident in the number of predicted taxon bins: for instance, when simulating novel families (Supplementary Figs S11–S13e), many more species bins were predicted by *CARMA3* (1672) and *MEGAN4* (824) than by *taxator-tk* (65), with 49 species being present in the sample. Similarly, *MEGAN4* predicted 69 orders, *CARMA3* 81 and *taxator-tk* 27, compared with the existing 32 orders in simArt49e (Fig. 3). Overall, taxonomic assignments of *taxator-tk* were more rarely to false taxa at low ranks than with the other methods, and instead were to higher-ranking correct taxa. The other two methods assigned a substantial amount of sequence data incorrectly to bins at the family level or below. This can be seriously misleading if the results were to be used to estimate species diversity or to reconstruct genomes. Therefore, *taxator-tk* is better suited for taxonomic profiling in addition to its primary task—the recovery of individual taxonomic sequence bins from shotgun datasets.

**Fig. 4.**
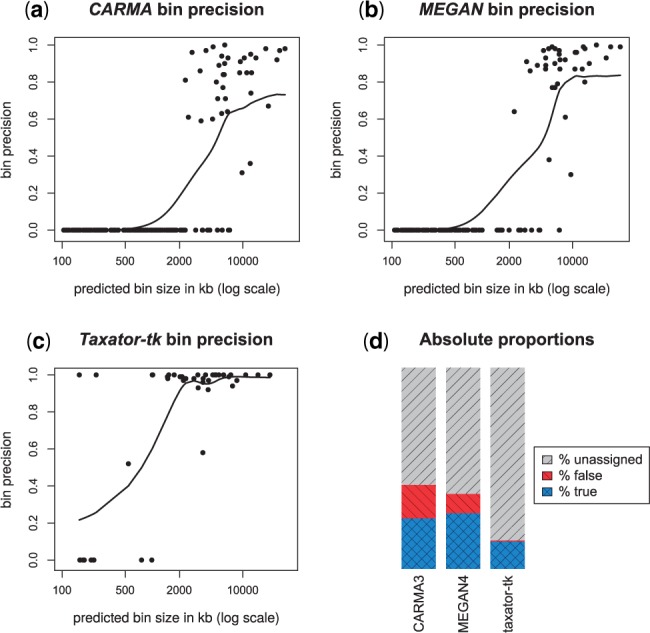
Family-level bin precision for the simulated metagenome sample with 49 species (simArt49e). (**a–c**) Each family bin’s assignment precision related to logarithmic bin size for seven cross-validation experiments with simArt49e. The results of the single experiments were added to assess the taxonomic assignment performance across a range of evolutionary distances between the query and the reference sequences, excluding the least abundant bins (1% of total basepairs). We calculated the precision values for (**a**) *CARMA3*, (**b**) *MEGAN4* and (**c**) *taxator-tk*, counting assignments to lower-ranking taxa at the family level, and added a smoothed *k*-nearest-neighbor estimate of the mean precision in R using *wapply* (width = 0.3) followed by *smooth.spline* (df = 10). *CARMA3* and *MEGAN4* incorrectly identified many small taxonomic bins, substantially more than *taxator-tk*. (**d**) The amount of correct, false and undetermined family-level assignments for the different classifiers with simArt49e

To investigate the reason for the observed differences between overall and macro-precision, which reflect variations in assignment precision for bins of different sizes, we plotted the per-bin precision at the family level in the combined cross-validation, as a function of predicted bin size with a *k*-nearest-neighbor (kNN) estimate of macro-precision (Fig. 4; see Supplementary Fig. S14 for all ranks). Overall, the bins predicted by *taxator-tk* were smaller, more precise and much more likely to represent truly existing taxa than those predicted by the other programs although larger bins tended to be more accurate for all methods. *CARMA3* and *MEGAN4* predicted a substantial number of mostly smaller-sized incorrect bins. Although the size-dependent kNN precision curves at large bin sizes is unaffected by these small bins, the curves remained below 70% (*CARMA3*) or 80% (*MEGAN4*), whereas the *taxator-tk* curve reached almost 100%. For the smallest bins, *taxator-tk*’s kNN precision was approximately 20% whereas bins below 500 kb for *CARMA3* and *MEGAN4* were practically indistinguishable from noise. This shows that the high macro-precision with *taxator-tk* is not only due to a lower frequency of falsely predicted bins, but also due to a substantially higher precision for the large bins.

Next, we performed cross-validation on the FAMeS (Mavromatis *et al*., 2007) SimMC/AMD (∼17 Mb/7307 contigs) and SimHC/soil (∼1 Mb/578 contigs) simulated metagenome datasets. These contigs were assembled from simulated Sanger (not NGS) reads and represent considerably smaller samples than those which are generated with the current NGS technologies (Dröge and McHardy, 2012). We also measured the methods’ performance on these data for a direct comparison to previous works. As before, *taxator-tk* had the highest macro-precision and the most realistic number of predicted taxon bins (Supplementary Figs S15–S20; Supplementary Material ‘XII. FAMeS cross-validation’).

For the contig assignments of the composition-based program *PhyloPythiaS* (Patil *et al*., 2011), we could not apply cross-validation, due to the computational effort of training many models. Therefore, we adopted the published evaluation scenario from Patil *et al* (2011), in which all genome sequences of the SimMC genera were removed from the reference genome sequence before classifying the contigs. All programs were provided with the remaining sequenced genomes and an additional 100 kb of reference data for each of the three dominant strains. The latter could be used by *PhyloPythiaS* to infer a corresponding species model, but were less helpful for the similarity-based classifiers. We generated assignments with *taxator-tk*, *CARMA3* and *MEGAN4/5* under equivalent conditions, once with nucleotide and once separately with protein local alignments, and compared them with both *Kraken* and the published *PhyloPythiaS* assignments (Supplementary Fig. S21). The performance and error distributions for the similarity-based programs (Supplementary Fig. S21c and d) were consistent with our previous evaluations with SimMC. *MEGAN4* and *MEGAN5* produced almost identical results. Using protein local alignments, we observed a moderate increase in overall species to family precision for *MEGAN5* and *CARMA3*, while *taxator-tk* improved in macro-recall. Notably, *taxator-tk* showed the best macro-precision of all similarity-based programs and all ranks, regardless of which alignment kind was used. *Kraken* produced most errors and the lowest macro-precision, because it assigned almost exclusively at species level. This would make it generally unsuitable in situations where sequences of closely related genomes are unavailable. However, it had a comparatively high macro-recall up to the order level.

Assignment with *PhyloPythiaS* showed that composition-based classification, when supplied with limited amounts of additional training data from the relevant species, correctly assigned most data at the genus and family levels (species assignments were not assessed in the original publication), which were either rarely assigned by *taxator-tk* or mostly incorrectly assigned by *CARMA3*, *MEGAN* and *Kraken*. However, *PhyloPythiaS* predicted only 6 families compared with 29 underlying families, versus 43 (*Kraken*), 14/18 (*taxator-tk*), 50/32 (*CARMA3*) and 17/18 (*MEGAN5*) with nucleotide or protein alignments, respectively. *PhyloPythiaS* had the highest macro-recall. The macro-precision (∼50% for genus, family and order level) was also higher than for *Kraken* (∼4–13%), *MEGAN* (∼7–31%) or *CARMA3* (∼7–48%) but less than for *taxator-tk* (∼32–68%). However, unlike for the other programs, the modeled taxa for *PhyloPythiaS* should be specified *a priori* to achieve optimal performance. It is therefore best applied when the taxonomic composition of a microbial community has already been determined and sufficient training data are available for the identified taxa.

### 3.3 Evaluation with real metagenome contigs

For microbial communities in many environments, only distantly related reference genome sequences are available. We analyzed a medium complex metagenome sample of such a microbial community from cow rumen (Hess *et al*., 2011) with *taxator-tk*, *CARMA3*, *MEGAN4/5* and *PhyloPythiaS* (the general model with the 100 most abundant species among sequenced prokaryotes). We considered scaffolds to be less reliable than contigs, which we reconstructed by splitting the available scaffolds at gaps of more than 200 positions (A.Sczyrba, personal communication). We subsequently divided contigs longer than 10 kb into sequence ‘chunks’ of 2 kb, resulting in a 319-Mb dataset, which we used to assess the assignment consistency for chunks originating from the same contig. The chunk sequences were assigned with *taxator-tk*, *CARMA3*, *MEGAN* (given identical nucleotide/protein alignments), *Kraken* and *PhyloPythiaS*. As the standard of truth for each contig, we determined the taxon minimizing the inconsistency between all corresponding chunk assignments (Gregor *et al.*, 2014; unpublished data) for each method independently. A chunk assignment was considered consistent, if it was to the same taxon as the assignment of the entire contig, and inconsistent otherwise. The consistency of a taxonomic bin is the fraction of chunk sequences with matching contig assignments and the macro-consistency is the consistency averaged over all predicted taxa, similar to the macro-precision.

In agreement with the results for the simulated metagenome datasets, the *taxator-tk* results were the most consistent among all tested methods, regardless of the alignment type (Supplementary Fig. S22): 76–89% macro-consistency at species-to-order level, in comparison to *MEGAN* (34–40%), *CARMA* (0–55%), *Kraken* (32–35%) and *PhyloPythiaS* (56–65%). The overall consistency (analogous to overall precision) for species-to-family levels was 97%/97% with *taxator-tk*, 39%/48% with *CARMA3*, 62%/64% with *MEGAN* (nucleotide/protein-level), 42% with *Kraken* and 82% with *PhyloPythiaS*. Likewise, *taxator-tk* assigned less data at species-to-family level, with a total of 13/12 Mb being consistent compared with *CARMA3* (8/26 Mb), *MEGAN* (42/47 Mb), *Kraken* (19 Mb) or *PhyloPythiaS* (14 Mb). The different methods again determined different numbers of taxa: *CARMA3* predicted 572/611 genera with a macro-consistency of 53%/31%, *MEGAN* 264/203 genera (34%/37%), *Kraken* 661 (32%), *PhyloPythiaS* 33 (63%) and *taxator-tk* found 110/27 genera (76%/81%). The high consistency values observed for *taxator-tk* indicate that it is a precise taxonomic classifier for real metagenomic contigs.

### 3.4 Run-time analyses

The run-time for the taxonomic metagenome assignment was measured as the time to find homologs and to assign taxon IDs to all sequences. We evaluated the run-times of all methods using the same set of alignments generated with either *BLAST* or *LAST*. Thus, the run-time for the initial similarity search was identical for all methods. We determined the time for the taxonomic assignment of simArt49e for all methods when performing a cross-validation with families present in the test dataset removed from the reference data (Fig. 3). This took 2 min with *Kraken* (single CPU core and ∼100 GiB RAM), 1 h for *MEGAN4* (interactive mode), 6 h for *taxator-tk* (∼10 CPU cores) and almost a week for *CARMA3* (∼20 CPU cores). The parallelization of *taxator-tk* led to a linear decrease in time with the number of CPU cores for up to 15 cores, which became sublinear for 20 cores or more (Supplementary Fig. S23). To provide a more specific estimate of the throughput of *taxator-tk*, we aligned ∼1 Gb of cow rumen sequence data with *BLAST* against *mRefSeq54* and assigned the data with *taxator-tk* on 10 CPU cores (AMD Opteron 6386 SE). We measured an average throughput of 5.9 Gb per day for the combined alignment and taxonomic assignment steps with this dataset. We also determined how our implementation scaled for increasing input sequence lengths and reference exclusion scenarios (Supplementary Fig. S24a). The run-time scaled approximately linearly except when the same or very similar species were among the reference genome sequences. In general, the greater the number of similar sequences in the reference data, the longer *taxator-tk’s* run-time was for the alignment of longer sequence stretches with more homologs. Simultaneously, we investigated the impact of the query segmentation on *taxator-tk’s* run-time (Supplementary Fig. S24b) and found that it reduced the total run-time by up to 30%.

## 4 Discussion

*Taxator-tk* is a taxonomic assignment software package which generates very precise taxonomic assignments with few errors for metagenome shotgun sequences. To provide a fair comparison, we invested extensive effort into ensuring that we evaluated all methods under identical conditions with the same reference sequences, test datasets and background taxonomies, using their recommended settings. We evaluated *taxator-tk* on 16S gene sequences, on simulated short reads, with simulated assembled contigs and with 2 kb contig fragments from a real cow rumen metagenome. For each simulated sample, we evaluated a wide range of evolutionary distances between the query and reference sequences using leave-one-taxon-out cross-validation. *Taxator-tk* was the most precise of all tested methods with the most realistic number of identified taxa overall. This property was very pronounced for lower taxonomic ranks from species-to-family level. However, *taxator-tk* assigned fewer data overall than other methods from species to family. For the small assembled SimMC dataset, it assigned fewer data, particularly in comparison to the composition-based classifier *PhyloPythiaS*, when 100 kb of data were provided for individual community members to train species-level models. For the real cow rumen dataset, *taxator-tk* was the most consistent in terms of classifying multiple pieces of one contig. Our results consistently indicate that *taxator-tk*’s strength is its high precision of assignments, which allows us to confidently assign a core of sample sequences and thereby to infer the taxonomic composition of the community. In comparison to assignments based on marker genes, it has the advantages that it makes assignments across all domains of life and that corresponding abundance estimates from shotgun sequences are less affected by copy number variations of individual genes. Such shotgun estimates are also unaffected by PCR primer amplification biases, unlike marker gene sequencing techniques, and do not require high-quality reference gene phylogenies for marker genes. We confirmed this by in depth analysis of six 15 Gb shotgun samples from the barley rhizosphere, where we applied *taxator-tk* to characterize the taxonomic composition of bacteria, archaea and eukaryotes, which correlated with results from 16S rRNA profiling and showed the most notable deviations for taxa known to be affected by primer biases or having multiple copies of the 16S rRNA gene (Bulgarelli *et al*., unpublished data). To target draft genome reconstructions, the data assigned to individual taxonomic bins by *taxator-tk* can be used as training data for complementary approaches, such as composition-based methods, or as independent information in combination with recently proposed clustering methods using the abundance of genes or contigs across multiple samples.

From a methodological point of view, we have introduced a method for the fast approximation of the evolutionary neighborhood of a query sequence with a run-time that increases linearly with the number of homologs. In *de-novo* phylogenetic inference methods, the run-time increases at least log-linearly with the number of homologs or they rely on time-consuming optimizations of parameter-rich phylogenetic models, which generates excessive computational requirements for the analysis of Gb-sized NGS samples. Our software provides an easy to use and scalable alternative to taxonomic classification of marker genes that is applicable to any nucleotide fragment. Unlike other similarity-based taxonomic classifiers for shotgun data, our algorithm handles different degrees of sequence conservation without preset or user-specified parameters such as alignment scores (overall or per gene family) and without being restricted to the analysis of a number of high-quality homologs with a minimal length. At the same time, the inferred evolutionary neighborhood is extended by the identification of an outgroup, leading to more precise taxonomic assignments, while regions without detectable taxonomic signal are instantly discarded. We post-process independent taxonomic assignments of query segments to infer an assignment for the entire query and do this using a majority vote algorithm with a few robust default parameters. This computationally lightweight step can be quickly repeated with other values for the majority and minimum support parameters, if required. In addition to the algorithmic considerations and other run-time optimizations, we implemented query sequence segmentation and program parallelization, which allow large-scale data analysis with a throughput of several Gbs per day on a standard multiprocessor system.

The program’s scope is also not limited to the taxonomic assignment of metagenomes: It can be applied to any DNA or RNA sequence. For instance, another successful in-house application is the detection of contaminations in isolate sequencing data. Furthermore, the program *taxator* within *taxator-tk* provides taxonomic information for individual query segments (Supplementary Figs S2 and S25), which could be used to identify assembly errors or regions acquired by lateral gene transfer.

## Supplementary Material

Supplementary Data
