# Supplementary material for: Taxator-tk: precise taxonomic assignment of metagenomes by fast approximation of evolutionary neighborhoods
Source: Bioinformatics. 2014 Nov 10;31(6):817–24. doi: 10.1093/bioinformatics/btu745 (PMC4380030; doi:10.1093/bioinformatics/btu745)
Supplement: Supplementary Data [file supp_31_6_817__index.html]

Taxator-tk: precise taxonomic assignment of metagenomes by fast approximation of evolutionary neighborhoods — Taxator-tk: precise taxonomic assignment of metagenomes by fast approximation of evolutionary neighborhoods — Taxator-tk: precise taxonomic assignment of metagenomes by fast approximation of evolutionary neighborhoods — Supplementary Data 

# *Taxator-tk*: precise taxonomic assignment of metagenomes by fast approximation of evolutionary neighborhoods

## Supplementary Data

files

**Files in this Data Supplement:**

- Supplementary Data - pdf file
