# Supplementary material for: Taxator-tk: precise taxonomic assignment of metagenomes by fast approximation of evolutionary neighborhoods
Source: Bioinformatics. 2014 Nov 10;31(6):817–24. doi: 10.1093/bioinformatics/btu745 (PMC4380030; doi:10.1093/bioinformatics/btu745)
Supplement: Supplementary Data [file supp_btu745_Supplementary_Material_Taxator-tk_updated.pdf]

Supplementary Methods for *Taxator-tk*: Precise Taxonomic Assignment of Metagenomes by Fast Approximation of Evolutionary Neighborhoods

I. Taxonomic Assignment of Sequence Segments

Here we describe in detail the individual steps and the run-time properties of the algorithm which is implemented in the program *taxator,* the second stage of the overall binning workflow using *taxator-tk* (Fig. 2b). We propose the realignment placement algorithm (RPA) for the taxonomic assignment of a query segment *q*, which can be any subsequence of the full query sequence (i.e. the query can be a read, contig, scaffold or a complete genome sequence). The algorithm constitutes **two pairwise alignment passes** and in each, *q* is aligned to segments of nucleotide reference sequences. It aims at identifying as many as possible taxa of the prediction clade (node R in Fig. 2a) without explicitly resolving its phylogenetic structure.

1. Among the given set of homologous segments constructed from overlapping alignments before application of the RPA, we define *s* to be the most similar segment to *q*, i.e. the one with the best local alignment score of all reference segments. In the first pass, all segments are aligned against *s* (n alignments). The resulting pairwise scores, our implementation uses the **edit distance** (mismatches + gaps), define an ordering among all segments or their corresponding taxa. The distinction between segments and associated taxa will be neglected in the following for better readability. All taxa which are less distant to *s* than *q*, including *s* itself, are added to an empty set *M* which holds all identified taxa of the prediction clade. The first more distant taxon than *q* is defined to be the outgroup segment *o* (Fig. 2c) and used as the alignment target in the following second and last pass in which similar taxa to *o* are added *M*.

2. We align all segments, including *q*, against *o* and rank the resulting scores. Then we add all taxa to *M* which have a lower score than *q*. With some fine-tuning, we chose to also add taxa with a higher score than *q*, within a small range accounting for erroneous scores, because *o* and *q* can be very distant homologs with noisy alignment. The width of this **error band** is determined on a per-segment basis as a linear score function of the taxonomic disorder in the alignment scores and not a universal or configurable run-time parameter. We interpret a rank disorder (e.g. a known family member of *o* being more similar to *o* than a

corresponding species member segment) as a discordance between gene tree and taxonomy and proportionally scale the effective score of *q* to enlarge *M* by taxa which are slightly more distant to *o* than *q*. This second pass requires $n-1$ new alignments, or less if some segments are identical to either *q* or *s*.

If multiple best references (*s)* or outgroup segments (*o)* were present in these two passes with identical alignment scores, the calculations are repeated for every such segment in order to produce stable output. We reduced the additional computational effort in our implementation by detecting frequent identical segments and uninformative homologs. The final assignment taxon ID of *q* is the lowest common ancestor (LCA) of the taxa in *M*, or none if no outgroup had been found. The theoretical run-time in the segment assignment algorithm measured in units "number of pairwise alignments" is in $O(n)$ and about $2n$, where n denotes the number of homologous segments. The run-time complexity for a single pairwise alignment is $O(l^2)$ and scales quadratically with the segment length l. Therefore the total run-time complexity per segment is $O(nl^2)$ and the total worst-case run-time for the entire query sequence can be bounded above by mL^2 where m denotes the maximum number n of segment homologs among all query segments and L is the total length of the query sequence. Thus, the run-time for the entire sample in the worst case scales linearly with the amount of sequence data (bp) and linearly with the number of homologs but quadratically with the length of the individual segments. Segments with an excessive number of homologs, most often short segments of abundant and uninformative regions, have a negative impact on the program run-time. We currently limit the number of homologs per query to the **top-scoring 50** by default in our pipeline scripts (configurable run-time parameter in program *alignments-filter* or directly in the local alignment search program), before passing them to *taxator*. Other tested **values gave similar results** and the parameter, if changed, should be chosen based on hardware limitations. If this parameter is set lower, then the number of reference segments drops below a critical value such that no outgroup can be determined for some *q* and which therefore remain unassigned (but without impacting the taxon ID of other segments).

II. Consensus Binning Algorithm

Due to sparse segments and taxonomic assignment thereof with *taxator* in stage two of the workflow (Fig. 1b), a final processing step (Fig. 1c) is required to determine

a taxon ID for the entire query sequence. Therefore we have implemented a simplistic, weighted consensus assignment scheme in the program *binner,* which optionally permits to apply custom constraints, e.g. the minimum percentage identity (PID) for classification at the species level or the removal of taxa with low counts in the whole sample. However, there are currently only **two mandatory run-time parameters** to control the actual post-processing consensus algorithm. First we define the support of a query segment to be the number of total identical positions to the best reference segment. The first run-time parameter specifies the **minimum combined support** at any rank (50 positions by default) and serves to ignore false predictions caused by short and often noisy segments. The other parameter specifies the **minimum percentage of the summed support** (70% by default) to allow a majority taxon to outvote a contradicting minority. Inconsistent taxa below this support value are resolved by the LCA operation until the threshold is reached. Probably due to the conservative nature of the RPA, we found those two parameters to have minimal impact on the binning results in practice. The output of taxator additionally includes the taxa in the evolutionary neighborhood, a score reflecting the agreement between the segment tree and the taxonomy, as well as a score for interpolation of the query-branch location between the R and X nodes of Fig. 2. We provide Python language bindings for processing with other applications.

III. Taxonomy and Phylogeny

Taxator-tk assumes that the NCBI taxonomy used for the assignment correctly captures the evolutionary process of speciation, although we know that the categorization of some taxa might be inconsistent with their evolution. If the phylogenetic information inferred from similarity scores disagrees with the taxonomic structure, assignments are made to a consistent higher rank. For instance horizontal gene transfer and upstream sequence misassembly can cause multiple similar copies of a sequence to be distributed across unrelated taxa. In case a query sequence cannot be traced by the algorithm to have evolved with either copy, it is usually assigned to the LCA of these clades. However, if the donor clade is unknown, the query may also be assigned to the recipient clade and the horizontal transfer or misassembly can go undetected. Thus assignment errors caused by the evolution of genes, upstream technical errors or taxonomy cannot always be eliminated in this framework. It remains to be assessed whether the use of an alternative microbial taxonomy such as the GreenGenes[1] or the SILVA[2] taxonomy would improve on the

taxonomic assignment.

IV. Comparison and Innovations

Taxator-tk shares some ideas with previous programs: Starting with *MEGAN*[3], which uses local alignments scores to define a "neighborhood of related sequences" and then makes a taxonomic estimate which is the LCA of the corresponding taxa. This neighborhood threshold is a percentage of the local alignment score and can be interpreted to reflect the rate of evolution within a taxonomic group. Its value is empirical and lacks stronger justification. The neighborhood definition has been improved in *taxator-tk* and other programs. To our knowledge, *SOrt-ITEMS*[4] was the first algorithm to use the logic of realignment to the best reference (termed reciprocal similarity) for read assignment but is restricted to protein level alignment and is implemented as a wrapper around (the legacy C version of) *BLAST+*[3]. Protein-level alignment in general triples the run-time of the local alignment step (translation into three frame shifts) and cannot make use of faster nucleotide aligners. *SOrt-ITEMS* also uses fixed similarity thresholds in terms of percentage identity to define universal levels of conservation within taxonomic groups assuming the same rate of evolution for different genetic regions and clades. Furthermore *SOrt-ITEMS* was primarily designed for reads and if it performs well for longer sequences, its run-time is expected to increase proportionally with input sequence lengths. Both follow-up programs *taxator-tk* and *CARMA3*[6] adopted the logic of reciprocal alignment, extended it and removed the assumption of universal conservation levels. *CARMA3* accounts for a heterogeneous rate of evolution for different genetic regions. The initial identification of similar sequences in the reference can be based on nucleotide or protein *BLAST* search or profile Hidden Markov Models with *HMMER*[7]. In *BLAST* mode, *CARMA3*, like *SOrt-ITEMS*, uses a single reciprocal alignment search and then extra or interpolates alignment scores to select a taxonomic rank for prediction. It therefore assumes a parameterized model for the conservation level at a taxonomic rank: a linear function which is fitted to the observed local alignment scores.

With *taxator-tk*, we use a non-parametric score ranking algorithm, instead. Also, to our knowledge, we provide the first algorithm to determine a proper outgroup and to sparsify the input data being able to assign distinct regions on the query sequence to possibly different taxonomic groups. Also, we at most assume segment-wise constant

rates of evolution (equally long branches from a common ancestor). This makes the major algorithmic component parameter-less and robust in itself, independent of the individual segment sizes. Through the sparsification procedure it incorporates structural rearrangements among distant relatives and scales better with the length of the input sequences. The individual segment assignments allow for a robust consensus voting scheme for the assignment of entire sequence fragments. The segment-specific classifications could also be used to detect the inconsistent taxonomic composition of an input sequence which can be caused by horizontal gene transfers events (HGTs) and assembly errors. Different from most previous approaches, *taxator-tk* was developed for and tested using fast nucleotide sequence local alignments instead of protein sequence alignments, although for the local alignments in stage 1 of the workflow both can be used. Our comparisons, however, suggest that the additional computations which are required for protein-level homology search do not considerably improve the results with *taxator-tk*. Thus, taxonomic binning of a metagenome sample with *taxator-tk* requires no more than specification of reference sequences, their taxonomic affiliations and an aligner like *BLAST* or *LAST*[8]. On the implementation side, all workflow steps for taxonomic assignment with *taxator-tk* are designed in a modular way making it easy to save, compress, reuse or recompute results. The computation-intensive classification of segments in *taxator* is run in parallel on many CPU cores while at the same time using the open source C++ algorithm library SeqAn[9] for fast pairwise alignment.

V. Performance Measures

As metagenome datasets can have varying taxonomic composition in terms of which taxa are present and their relative abundances, this needs to be taken into consideration in evaluating taxonomic assignment methods. If an algorithm performs better for some clades than for others at a given rank we call it taxonomically biased. Oftentimes a classifier is biased, if it uses parameters that fit one clade better than another. This can be the case if the parameters were chosen to give good overall assignment accuracy (low total number of false predictions) on training data with biased taxonomic composition. Such a method is optimized to perform well for the abundant taxa of these particular training data and will not generalize well when applied to a sample of different taxonomic structure and abundances. To account for uneven taxonomic composition in evaluation datasets and to obtain comparable performance estimates across datasets of different taxonomic composition, we used

as the primary evaluation measure the bin-averaged **precision** (or **positive predictive value**), also known as **macro-precision**.

$$\text{macro-precision} = \frac{1}{N_p}\sum_{i=1}^{N_p}\text{precision}_i \qquad \text{(Equation V.1)}$$

where N_p is number of all predicted bins and

$$\text{precision}_i = \frac{\text{TP}_i}{\text{TP}_i + \text{FP}_i} \qquad \text{(Equation V.2)}$$

True positives TP_i are the correct assignments to the ith bin and false positives FP_i the incorrect assignments to the same bin.

The macro-precision is the fraction of correct sequence assignments over all assignments to a given taxonomic bin, averaged over all predicted bins for a given rank. For falsely predicted bins which do not occur in the data, the precision is therefore zero. This value reflects how trustworthy the bin assignments are on average from a user's perspective, as it is averaged overall predicted bins.

In addition to the macro-precision, we report the raw numbers of true and false predictions for every cross-validation, as well as a quick overall precision for pooled ranks. This overall precision is most informative for species+genus+family and reports the fraction of true classifications among the predictions for all these ranks in a single pooled bin.

$$\text{overall-precision} = \frac{\text{TP}}{\text{TP} + \text{FP}} \qquad \text{(Equation V.3)}$$

We measure the taxonomic bias of a method in terms of the standard deviation over all individual bin precisions.

$$\text{sd}_{prec} = \sqrt{\frac{1}{N_p}\sum_{i=1}^{N_p}\left(\text{precision}_i - \overline{\text{precision}}\right)^2} \qquad \text{(Equation V.4)}$$

where

$$\overline{\text{precision}} = \frac{1}{N_p}\sum_{i=1}^{N_p}\text{precision}_i \qquad \text{(Equation V.5)}$$

The standard deviation is small if all predicted bins have a similar precision. A universally good method should have a high macro-precision with a low taxonomic

bias.

The **recall** (or **sensitivity**) is a measure of completeness of a predicted bin and, analogously, the **macro-recall** is the fraction of correctly assigned sequences of all sequences belonging to a certain bin, averaged over all existing bins in the test data[10].

$$\text{macro-recall} = \frac{1}{N_r}\sum_{i=1}^{N_r}\text{recall}_i \qquad \text{(Equation V.6)}$$

where N_r is the number of all existing bins in the test data and

$$\text{recall}_i = \frac{\text{TP}_i}{\text{TP}_i + \text{FN}_i} \qquad \text{(Equation V.7)}$$

False negatives (FN_i) are the assignments belonging to the ith bin but which where classified to another bin or left unassigned.

The macro-recall reflects how well the classifier works more from a developer's perspective than from the user's perspective, as it is usually not known which predicted bins correspond to existing ones and which do not.

VI. Low-abundance Filtering

The number of predicted bins at each rank can be quite large, at most the number of known taxa in the taxonomy and reference sequence data. When noise is considered to occur evenly distributed across this large output space, bins with few assigned sequences are more likely to be falsely identified, than larger bins (the chance to independently classify the same bin by chance *n* times is $\left(\frac{1}{m}\right)^n$, where m is the number of possible bins). Since the macro precision is an average over all predicted bins, it is heavily affected by bins with few sequences assigned. As a result, classifiers that predict clades present at low frequencies in the sample score badly under this measure. To correct for this effect, we define a truncated average precision ignoring the least abundant predicted bins and consider only the **largest predicted bins constituting a minimum fraction α of the total assignments** (equal size bins are also included). This modification acts as a noise filter and accounts for different behavior of classifiers without explicitly considering the size of the model space or the number of existing species in the actual sample. We set α to 0.99 for our evaluations.

VII. Cross-validation

Despite the limitations of simulated metagenomes, which incorporate assumptions about sequencing error rates or species abundance distributions, it is very informative to evaluate taxonomic assignment methods on simulated sequence data as real metagenome samples lack taxon IDs for evaluation. Our canonical way of evaluating a method on simulated data is a version of **leave-one-out cross-validation**: Each query sequence is classified by removing all identical or related sequences up to a given rank from the reference collection: For example, to assess the performance in assigning query sequences from a new species, all sequences belonging to this species are removed from the reference sequence collection for the classifier. Performance measures (macro-recall, macro-precision), along with other statistics (true/false/unassigned data, overall precision, bin counts) which are available in the coupled tables, were normally calculated in units of the number of assigned basepairs or the number of assigned sequences, if these had comparable lengths. These values were calculated for all ranks (species, genus, family, order, class, phylum, domain/superkingdom) for seven simulations: either all reference data was used (per query) or all data from **the query** species, genus, family, order, class or phylum was removed from the reference data prior to classification. The assignments of these seven cross-validation experiments were averaged for a combined performance summary with standard measures.

VIII. Consistency Analysis

In order to evaluate the predictions for real metagenome samples where no underlying correct taxon IDs are known for the sequences, we assigned sequences linked by assembly and calculated an assignment consistency value. We split long contigs into multiple pieces and classified each piece independently. Assuming that the sequence assembly was correct in the first place, contradicting assignments of pieces that originate from the same contig represent false assignments. This unveils part of the errors made by a particular method but some, if not the majority, will go undetected because the actual ID stays unknown and the assignments for a contig can be consistently wrong. Hence these results are generally more difficult to interpret than those from simulated data.

IX. Sequence Homology Search via Local Alignment

In the course of evaluation we created many local alignments as input to the

taxonomic assignment programs *CARMA3*, *MEGAN4/5* and *taxator-tk*. The nucleotide alignments were mostly generated using the alignment program *LAST* (version 320) because it ran faster without noticeable differences in the output alignments than *BLAST+/blastn (version 2.2.28+)*. The protein-level alignments which we used in our evaluations were generated with *BLAST+/tblastx (version 2.2.28+)* because we wanted to compare with identical nucleotide reference sequences. We support and tested with different alignment programs for the fact that *BLAST* is standard and easy to parallelize whereas *LAST* has a faster algorithm but high memory requirements. It ran with comparable speed to the *BLAST+/megablast* algorithm which has a limited sensitivity and in practice resulted in a two to four times reduced amount of query sequences being aligned and classified. For a detailed comparison of alignment programs and how LAST compares to other programs such as *RAPSEARCH2*[11] and *BLAT*[12], consider Niu et al.[13] and Darling et al.[14]. In our evaluations, *LAST* was roughly 50 to 200 times faster than *BLAST+/blastn* and about as fast as *BLAST+/megablast* (which has much reduced sensitivity). *LAST* is also tunable for better sensitivity with protein-coding nucleotide sequences using a special form of seeding. If other alignment programs are found to be better-suited for a particular data type, these can easily be incorporated into the provided workflows. For instance, local protein sequence alignments can be performed in the homology search step, e.g. by using *BLAST+/tblastx*. There are fast aligners such as *RAPSEARCH2*, *PAUDA*[15] and *DIAMOND*[16] that allow searching for homologs in large reference collections of amino acid sequences. To produce compatible input for *taxator-tk*, the amino acid alignment positions must be converted into nucleotide positions.

For our short sequence length evaluation (Supplementary Fig. S6-S8), evaluation of a published SimMC scenario (Supplementary Fig. S21) and evaluation of a simulated metagenome sample with 49 species (Fig. 3, Supplementary Fig. S11-S13), we used a standard *BLAST+/blastn (version 2.2.28+)* and *BLAST+/tblastx* search. We chose the default alignment parameters and scoring schemes with each aligner. The generated alignments were then provided in *BLAST* tabular format to be usable with *CARMA3* and *MEGAN4/MEGAN5*. *Taxator-tk* reads a simplistic tab-separated alignment format that can be generated directly with *BLAST+* or with conversion scripts which we provide for the MAF alignment format of *LAST*. This arrangement ensures that *taxator-tk* can be easily adapted to profit from

advancements in the field of local alignment in future. Users can also employ amino acid level alignment if the final output is mapped back to positions on the nucleotide reference and query sequences. The easiest way to achieve this is to use *BLAST+/tblastx* although this is computationally more demanding than directly searching a collection of protein sequences for which also nucleotide sequences are available.

X. Program Parameters and Versions

For taxonomic assignment with *MEGAN4* (version 4.70.4) we used minscore=20, toppercent=20, minsupport=5 and mincomplexity=0.44 parameters. With *MEGAN5* (version 5.4.3), we used the default options minsupport=10, minscore=50, max_expected=0.01, minimal_coverage_heuristic=on and top_percent=20, as with *MEGAN4*. In *CARMA3*, we used the standard parameters in the contained configuration file. *Kraken* (version 0.10.4b) was also applied with the standard commands and without shrinking the database (shrink_db.sh). *Taxator-tk* (version 1.1.1-extended) was run with standard settings, being restricted to the 50 best scoring local alignments to avoid long run-times for some of the query sequences. This is purely a convenience filter at the current state of development and is meant to be replaced by an adaptive per-segment heuristic.

XI. 16S Cross-validation

We evaluated the performance of *taxator-tk* in classifying the most widely used taxonomic marker gene in studies of microbial diversity, the 16S rRNA gene, as a proof of concept. For our evaluation, we extracted 7,175 annotated 16S rRNA genes (Suppl. Fig. 5) each with a minimum length of 1 kb from *mRefSeq47* (Suppl. Fig. 9). The sequences were assigned with *taxator-tk* using the entire mRefSeq as reference, not just 16S genes. The cross-validation assesses the performance of 16S gene assignment in a wide range of situations. The performance statistics were calculated based on the number of assigned sequences, as all have comparable length. When using the complete reference sequences, 87% of sequences were assigned to the ranks of species, genus and family with 100% accuracy (Supplementary Fig. S3b), the remaining 13% were correctly assigned at higher ranks. This is an ideal situation showing the baseline on our dataset (in terms of the assigned rank depth). In more realistic simulations, when we tested assignment of genes from novel species or novel higher-level clades, assignments were accordingly made to higher ranks in

most cases. For instance, when simulation novels species, 2,678 contigs were assigned to the correct genera, while 491 erroneous species and genus assignments were made. The macro-precision in the combined cross-validation (Fig. 2) was always above 92%, with standard deviations from 10 to 25%, which demonstrates a good and even performance of *taxator-tk* for all clades in the case of 16S rRNA data.

XII. FAMeS Cross-validation

On the FAMeS contig datasets, taxator-tk produced fewer errors for all taxonomic ranks than *MEGAN4*, which was accompanied by a moderate reduction in macro-recall throughout all individual experiments and in the combined cross-validation experiments: For SimMC, the macro-precision was three to four times as large as *MEGAN4*'s for species to order, with higher macro-recall (Supplementary Fig. S17-S18). The species to family overall precision was ~91% for *taxator-tk* (~59% for *MEGAN4*) and taxator-tk estimated 54 species bins (*MEGAN4* 188) for the 47 actual species in SimMC. Similarly, for SimHC, *taxator-tk* achieved a higher macro-precision for all ranks, which was most pronounced for class and phylum (Supplementary Fig. S19-S20). By contrast, the macro-recall was slightly reduced and both methods underestimated the 96 existing species in SimHC.

XIII. Supplementary Files

The PDF attachment includes informative interactive charts and files which are necessary to reproduce the results which are shown in the article. Larger benchmark data can be downloaded from http://algbio.cs.uni-duesseldorf.de/software/.

Supplementary Methods References

1. DeSantis, T. Z. *et al.* Greengenes, a chimera-checked 16S rRNA gene database and workbench compatible with ARB. *Appl. Environ. Microbiol.* **72,** 5069–72 (2006).
2. Quast, C. *et al.* The SILVA ribosomal RNA gene database project: improved data processing and web-based tools. *Nucleic Acids Res.* **41,** D590–6 (2013).
3. Huson, D. H., Auch, A. F., Qi, J. & Schuster, S. C. MEGAN analysis of metagenomic data. *Genome Res.* **17,** 377–86 (2007).
4. Monzoorul Haque, M., Ghosh, T. S., Komanduri, D. & Mande, S. S. SOrt-ITEMS: Sequence orthology based approach for improved taxonomic estimation of metagenomic sequences. *Bioinformatics* **25,** 1722–30 (2009).
5. Camacho, C. *et al.* BLAST+: architecture and applications. *BMC Bioinformatics* **10,** 421 (2009).

6. Gerlach, W. & Stoye, J. Taxonomic classification of metagenomic shotgun sequences with CARMA3. *Nucleic Acids Res.* 1–11 (2011).

7. Finn, R. D., Clements, J. & Eddy, S. R. HMMER web server: interactive sequence similarity searching. *Nucleic Acids Res.* **39 Suppl 2,** W29–37 (2011).

8. Frith, M. C., Hamada, M. & Horton, P. Parameters for accurate genome alignment. *BMC Bioinformatics* **11,** 80 (2010).

9. SeqAn. at http://www.seqan.de

10. McHardy, A. C., Martín, H. G., Tsirigos, A., Hugenholtz, P. & Rigoutsos, I. Accurate phylogenetic classification of variable-length DNA fragments. *Nat. Methods* **4,** 63–72 (2007).

11. Zhao, Y., Tang, H. & Ye, Y. RAPSearch2: a fast and memory-efficient protein similarity search tool for next generation sequencing data. *Bioinformatics* **28,** 125–126 (2011).

12. Kent, W. J. BLAT–the BLAST-like alignment tool. *Genome Res.* **12,** 656–64 (2002).

13. Niu, B., Zhu, Z., Fu, L., Wu, S. & Li, W. FR-HIT, a very fast program to recruit metagenomic reads to homologous reference genomes. *Bioinformatics* **27,** 1704–5 (2011).

14. Darling, A. E. *et al.* PhyloSift: phylogenetic analysis of genomes and metagenomes. *PeerJ* **2,** e243 (2014).

15. Huson, D. H. & Xie, C. A poor man's BLASTX--high-throughput metagenomic protein database search using PAUDA. *Bioinformatics* **30,** 38–9 (2014).

16. Buchfink, B., Xie, C. & Huson, D. H. Fast and Sensitive Protein Alignment using DIAMOND, under review.

Supplementary Figure S1: Query sequence segmentation and segment splicing

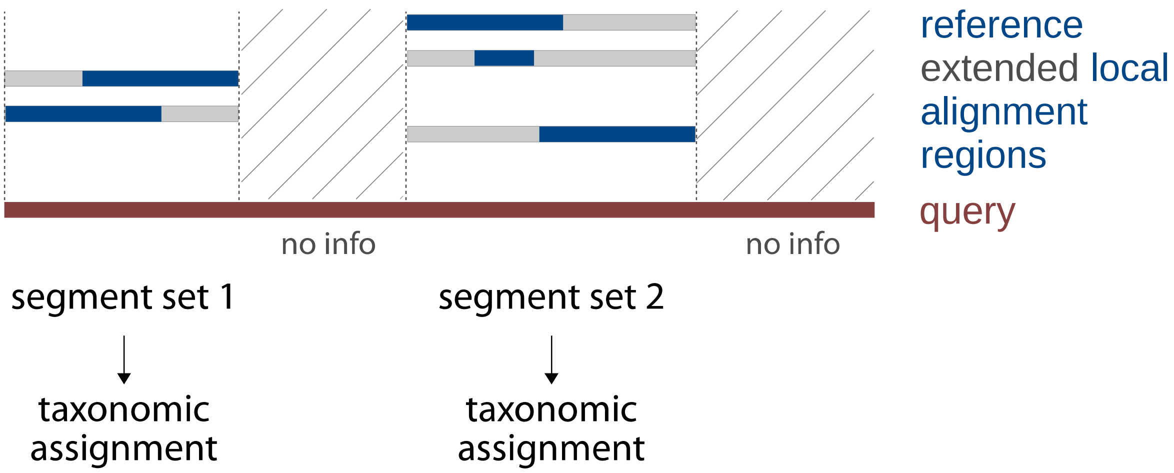

Query and corresponding reference segments from local alignment region extension and splicing. Blue bars correspond to original local alignment regions on reference nucleotide sequences which are positionally aligned to the query nucleotide sequence in red. These alignments are generated by a local (nucleotide) sequence aligner such as *BLAST* or *LAST* before running *taxator*. If alignments overlap on the query, they are joined into query segments which are flanked by regions without detected similarity to any known reference sequence. Reference segments are constructed from the original alignment reference regions (blue) by extension (gray bars) with the same number of nucleotides which are missing to match the length of the query segment. The corresponding sets of homologs are the input to the core taxonomic assignment algorithm in *taxator*.

Supplementary Figure S2: Taxonomic assignment of segments

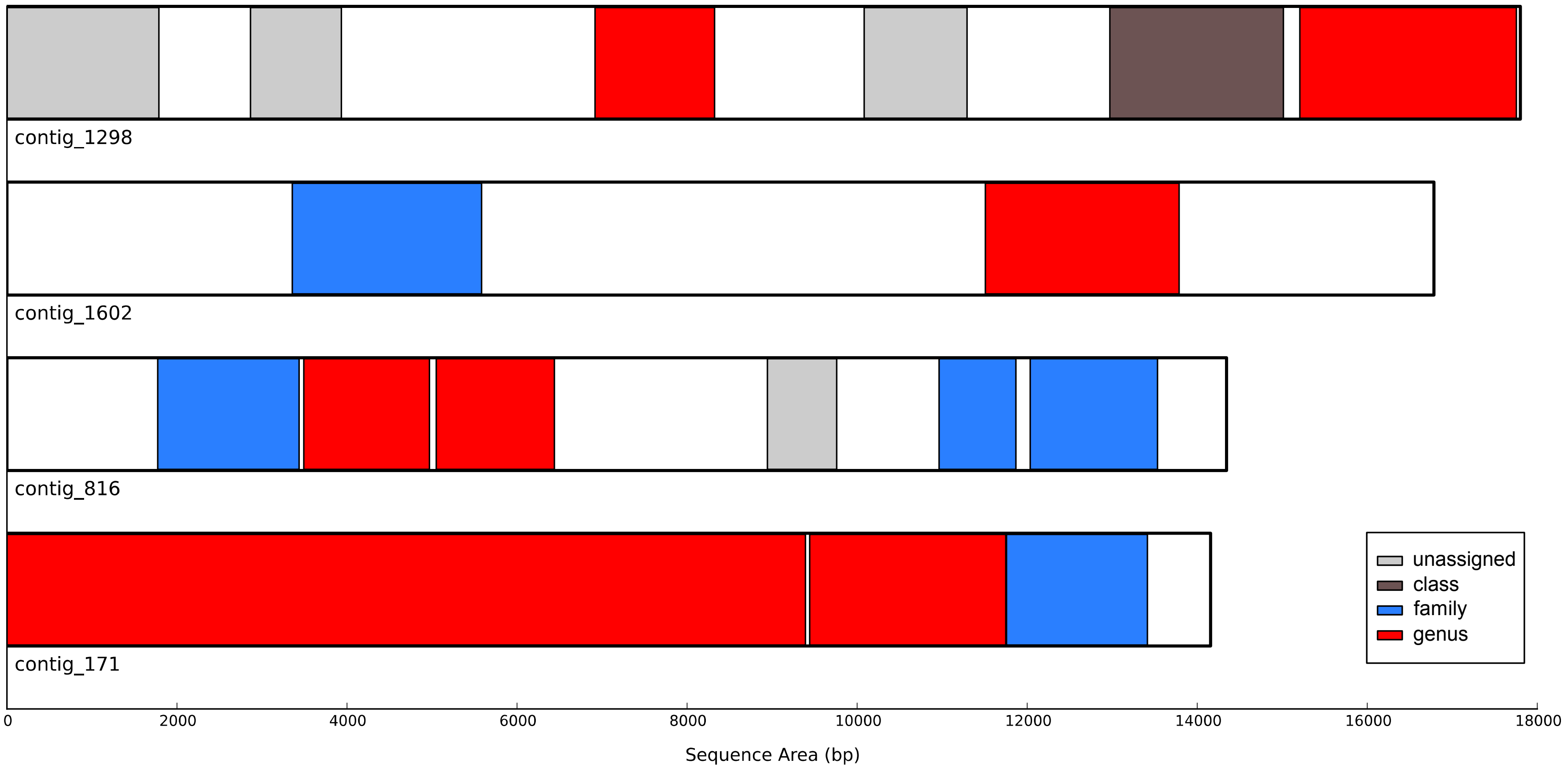

Four long contigs of the SimMC data-set. Colored boxes show segments that where assigned by *taxator*, when all species reference data was removed (new species simulation). White regions in between lack alignments by the local alignment search and have therefore no homologs for assignment. All assigned regions in this example are consistently assigned at the taxonomic ranks genus, family and class. The shown segments are used by the program *binner* to derive consistent whole-sequence taxonomic assignments, as done in our evaluations.

Supplementary Figure S3 - 16S gene assignment with taxator-tk

(a) summary scenario

rank	depth	true (sequences)	false (sequences)	unknown (sequences)	macro precision α=0.99	stdev	pred. bins	macro recall	stdev	real bins	sum true (sequences)	sum false (sequences)	overall prec.	description
unassigned	0	274.4	0.0	0	100.0	0.0	1	100.0	0.0	1	4593.6	0.0	100.0	root+superkingdom
superkingdom	1	2159.6	0.0	0	100.0	0.0	2	82.7	14.2	2				
phylum	2	869.1	66.6	0	95.5	13.8	13	33.5	23.6	32	2707.1	228.6	92.2	phylum+class+order
class	3	1417.9	92.6	0	96.1	10.7	25	27.3	18.4	52				
order	4	420.1	69.4	0	95.4	12.6	62	20.7	14.2	109				
family	5	471.6	26.1	0	95.7	13.5	148	16.0	11.9	235	1732.1	174.1	90.9	family+genus+species
genus	6	636.7	65.0	0	95.8	16.1	342	9.2	8.9	615				
species	7	623.8	83.0	0	92.6	24.5	570	5.1	6.7	1416				
avg/sum	2.6	6598.8	402.7	0	95.9	13.0	166.0	27.8	14.0	351.6			94.2	all but unassigned
avg/sum	2.6	6873.3	402.7	0	96.4	11.4	145.4	36.8	12.2	307.8			94.5	all with unassigned

taxator-tk on RefSeq 16S genes

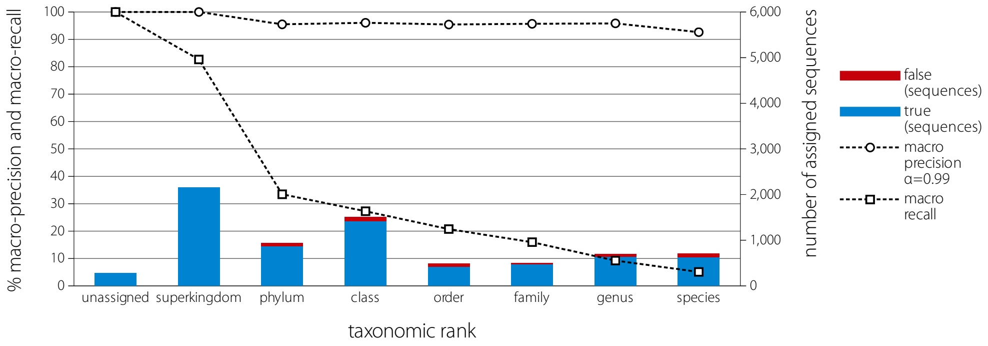

Supplementary Figure S3 - 16S gene assignment with taxator-tk

(b) all reference scenario

rank	depth	true (sequences)	false (sequences)	unknown (sequences)	macro precision α=0.99	stdev	pred. bins	macro recall	stdev	real bins	sum true (sequences)	sum false (sequences)	overall prec.	description
unassigned	0	10	0	0	100.0	0.0	1	100.0	0.0	1	170	0	100.0	root+superkingdom
superkingdom	1	80	0	0	100.0	0.0	2	97.6	2.4	2				
phylum	2	113	0	0	100.0	0.0	16	78.3	38.3	32	813	0	100.0	phylum+class+order
class	3	428	0	0	100.0	0.0	29	77.6	37.2	52				
order	4	272	0	0	100.0	0.0	67	72.4	39.6	109				
family	5	750	0	0	100.0	0.0	158	71.0	40.4	235	6272	0	100.0	family+genus+species
genus	6	1779	0	0	100.0	0.0	337	53.9	48.0	615				
species	7	3743	0	0	100.0	0.0	504	35.8	46.8	1416				
avg/sum	5.0	7165	0	0	100.0	0.0	159.0	69.5	36.1	351.6			100.0	all but unassigned
avg/sum	5.0	7175	0	0	100.0	0.0	139.3	73.3	31.6	307.8			100.0	all with unassigned

taxator-tk on RefSeq 16S genes

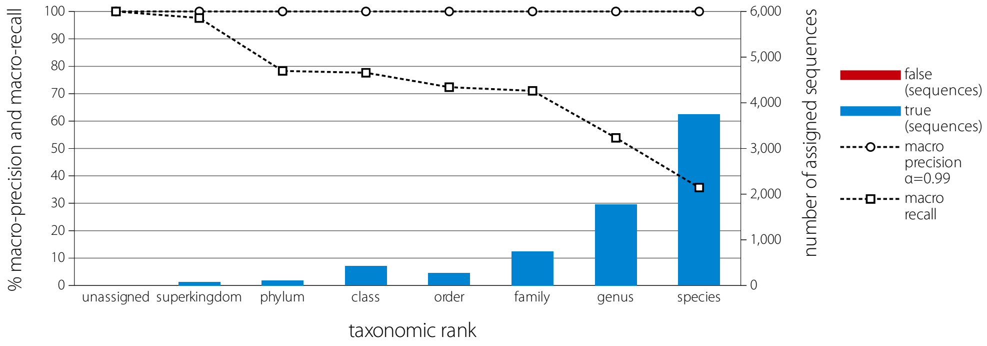

Supplementary Figure S3 - 16S gene assignment with taxator-tk

(c) new species scenario

rank	depth	true (sequences)	false (sequences)	unknown (sequences)	macro precision α=0.99	stdev	pred. bins	macro recall	stdev	real bins	sum true (sequences)	sum false (sequences)	overall prec.	description
unassigned	0	22	0	0	100.0	0.0	1	100.0	0.0	1	648	0	100.0	root+superkingdom
superkingdom	1	313	0	0	100.0	0.0	2	96.7	3.2	2				
phylum	2	347	2	0	100.0	0.0	14	54.8	39.5	32	2284	19	99.2	phylum+class+order
class	3	989	8	0	98.9	3.9	26	53.9	41.6	52				
order	4	948	9	0	98.8	7.9	54	44.2	39.6	109				
family	5	1350	18	0	98.2	7.3	91	28.8	37.5	235	4028	509	88.8	family+genus+species
genus	6	2678	64	0	95.2	18.9	88	10.8	26.3	615				
species	7	0	427	0	0.0	0.0	54	0.0	0.0	1416				
avg/sum	4.6	6625	528	0	84.4	5.4	47.0	41.3	26.8	351.6			92.6	all but unassigned
avg/sum	4.6	6647	528	0	86.4	4.7	41.3	48.6	23.5	307.8			92.6	all with unassigned

taxator-tk on RefSeq 16S genes

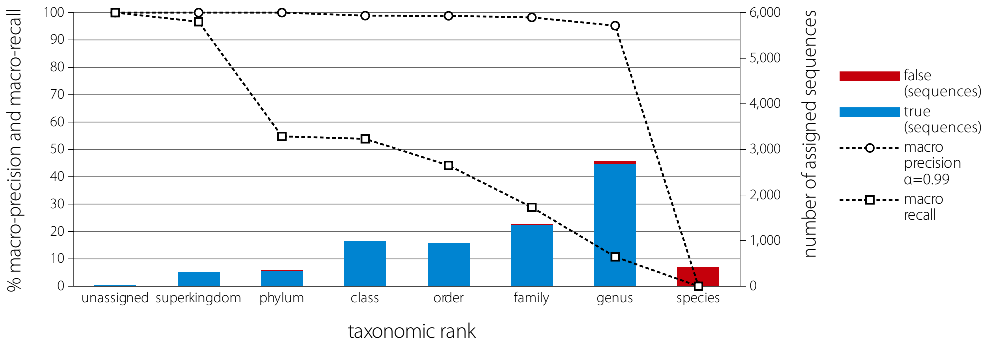

Supplementary Figure S3 - 16S gene assignment with taxator-tk

(d) new genus scenario

rank	depth	true (sequences)	false (sequences)	unknown (sequences)	macro precision α=0.99	stdev	pred. bins	macro recall	stdev	real bins	sum true (sequences)	sum false (sequences)	overall prec.	description
unassigned	0	48	0	0	100.0	0.0	1	100.0	0.0	1	1656	0	100.0	root+superkingdom
superkingdom	1	804	0	0	100.0	0.0	2	96.5	3.0	2				
phylum	2	1098	2	0	100.0	0.0	12	46.8	38.9	32	4680	19	99.6	phylum+class+order
class	3	2392	8	0	98.5	4.7	22	36.3	35.7	52				
order	4	1190	9	0	95.4	17.8	48	25.2	32.9	109				
family	5	1201	36	0	78.8	39.2	59	11.7	26.7	235	1201	423	74.0	family+genus+species
genus	6	0	344	0	0.0	0.0	34	0.0	0.0	615				
species	7	0	43	0	0.0	0.0	8	0.0	0.0	1416				
avg/sum	3.3	6685	442	0	67.5	8.8	26.4	30.9	19.6	351.6			93.8	all but unassigned
avg/sum	3.3	6733	442	0	71.6	7.7	23.3	39.6	17.2	307.8			93.8	all with unassigned

taxator-tk on RefSeq 16S genes

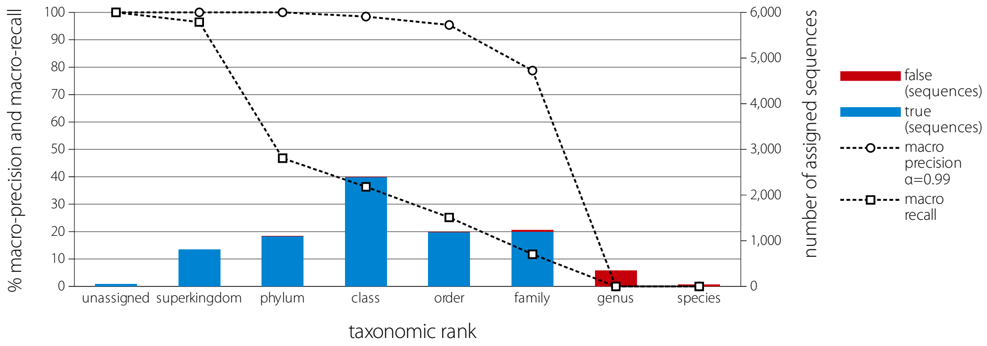

Supplementary Figure S3 - 16S gene assignment with taxator-tk

(e) new family scenario

rank	depth	true (sequences)	false (sequences)	unknown (sequences)	macro precision α=0.99	stdev	pred. bins	macro recall	stdev	real bins	sum true (sequences)	sum false (sequences)	overall prec.	description
unassigned	0	299	0	0	100.0	0.0	1	100.0	0.0	1	2941	0	100.0	root+superkingdom
superkingdom	1	1321	0	0	100.0	0.0	2	82.8	13.7	2				
phylum	2	1442	2	0	100.0	0.0	7	25.4	35.2	32	5458	28	99.5	phylum+class+order
class	3	3485	11	0	97.7	7.2	13	15.0	26.2	52				
order	4	531	15	0	69.6	42.6	28	3.4	12.1	109				
family	5	0	38	0	0.0	0.0	24	0.0	0.0	235	0	69	0.0	family+genus+species
genus	6	0	17	0	0.0	0.0	9	0.0	0.0	615				
species	7	0	14	0	0.0	0.0	3	0.0	0.0	1416				
avg/sum	2.4	6779	97	0	52.5	7.1	12.3	18.1	12.5	351.6			98.6	all but unassigned
avg/sum	2.4	7078	97	0	58.4	6.2	10.9	28.3	10.9	307.8			98.6	all with unassigned

taxator-tk on RefSeq 16S genes

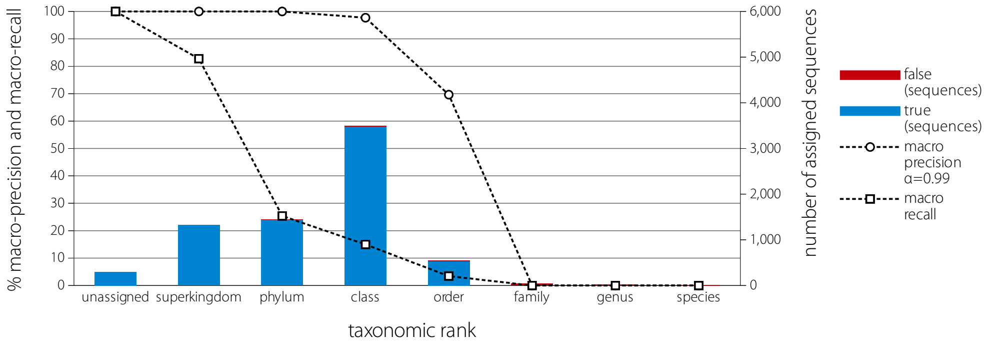

Supplementary Figure S3 - 16S gene assignment with taxator-tk

(f) new order scenario

rank	depth	true (sequences)	false (sequences)	unknown (sequences)	macro precision α=0.99	stdev	pred. bins	macro recall	stdev	real bins	sum true (sequences)	sum false (sequences)	overall prec.	description
unassigned	0	424	0	0	100.0	0.0	1	100.0	0.0	1	4264	0	100.0	root+superkingdom
superkingdom	1	1920	0	0	100.0	0.0	2	72.9	22.3	2				
phylum	2	1665	2	0	100.0	0.1	6	19.1	31.6	32	4296	448	90.6	phylum+class+order
class	3	2631	12	0	99.6	1.1	8	7.9	20.8	52				
order	4	0	434	0	0.0	0.0	17	0.0	0.0	109				
family	5	0	74	0	0.0	0.0	11	0.0	0.0	235	0	87	0.0	family+genus+species
genus	6	0	2	0	0.0	0.0	3	0.0	0.0	615				
species	7	0	11	0	0.0	0.0	1	0.0	0.0	1416				
avg/sum	2.1	6216	535	0	42.8	0.2	6.9	14.3	10.7	351.6			92.1	all but unassigned
avg/sum	2.1	6640	535	0	49.9	0.1	6.1	25.0	9.3	307.8			92.5	all with unassigned

taxator-tk on RefSeq 16S genes

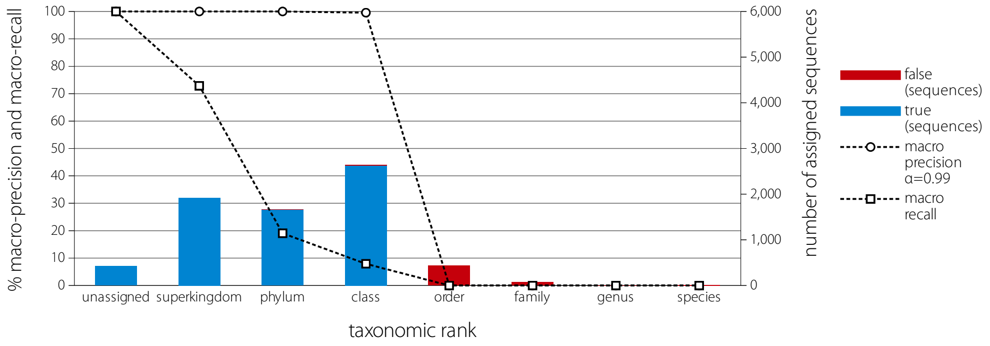

Supplementary Figure S3 - 16S gene assignment with taxator-tk

(g) new class scenario

rank	depth	true (sequences)	false (sequences)	unknown (sequences)	macro precision α=0.99	stdev	pred. bins	macro recall	stdev	real bins	sum true (sequences)	sum false (sequences)	overall prec.	description
unassigned	0	549	0	0	100.0	0.0	1	100.0	0.0	1	10017	0	100.0	root+superkingdom
superkingdom	1	4734	0	0	100.0	0.0	2	73.7	19.6	2				
phylum	2	1419	67	0	90.3	15.4	4	9.8	23.9	32	1419	460	75.5	phylum+class+order
class	3	0	390	0	0.0	0.0	8	0.0	0.0	52				
order	4	0	3	0	0.0	0.0	8	0.0	0.0	109				
family	5	0	9	0	0.0	0.0	6	0.0	0.0	235	0	13	0.0	family+genus+species
genus	6	0	1	0	0.0	0.0	2	0.0	0.0	615				
species	7	0	3	0	0.0	0.0	1	0.0	0.0	1416				
avg/sum	1.2	6153	473	0	27.2	2.2	4.4	11.9	6.2	351.6			92.9	all but unassigned
avg/sum	1.2	6702	473	0	36.3	1.9	4.0	22.9	5.4	307.8			93.4	all with unassigned

taxator-tk on RefSeq 16S genes

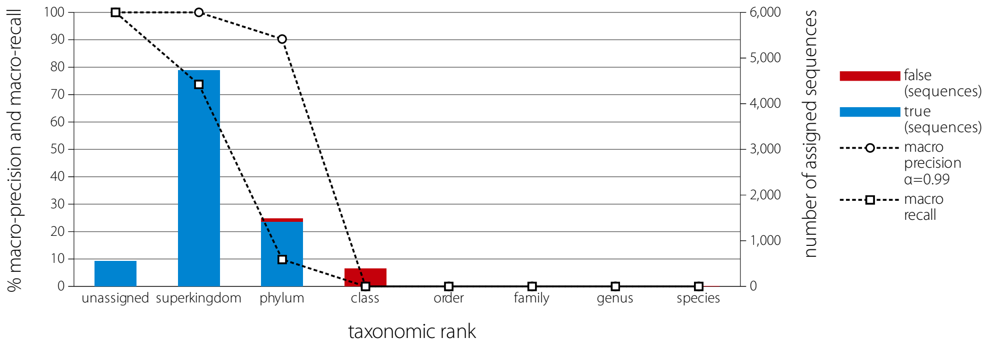

Supplementary Figure S3 - 16S gene assignment with taxator-tk

(h) new phylum scenario

rank	depth	true (sequences)	false (sequences)	unknown (sequences)	macro precision α=0.99	stdev	pred. bins	macro recall	stdev	real bins	sum true (sequences)	sum false (sequences)	overall prec.	description
unassigned	0	569	0	0	100.0	0.0	1	100.0	0.0	1	12459	0	100.0	root+superkingdom
superkingdom	1	5945	0	0	100.0	0.0	1	58.5	35.3	2				
phylum	2	0	391	0	0.0	0.0	5	0.0	0.0	32	0	626	0.0	phylum+class+order
class	3	0	219	0	0.0	0.0	8	0.0	0.0	52				
order	4	0	16	0	0.0	0.0	7	0.0	0.0	109				
family	5	0	8	0	0.0	0.0	5	0.0	0.0	235	0	35	0.0	family+genus+species
genus	6	0	27	0	0.0	0.0	2	0.0	0.0	615				
species	7				nan	nan	0	0.0	0.0	1416				
avg/sum	1.1	5945	661	0	16.7	0.0	4.0	8.4	5.0	351.6			90.0	all but unassigned
avg/sum	1.1	6514	661	0	28.6	0.0	3.6	19.8	4.4	307.8			90.8	all with unassigned

taxator-tk on RefSeq 16S genes

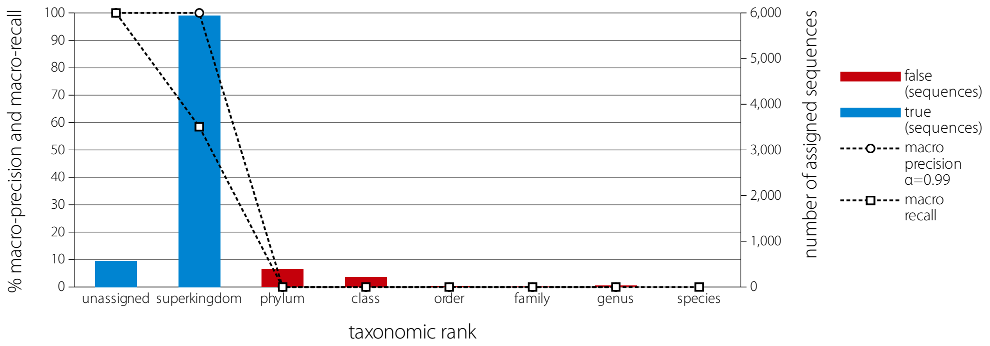

Supplementary Figure S4: Taxonomic composition of microbial RefSeq 47

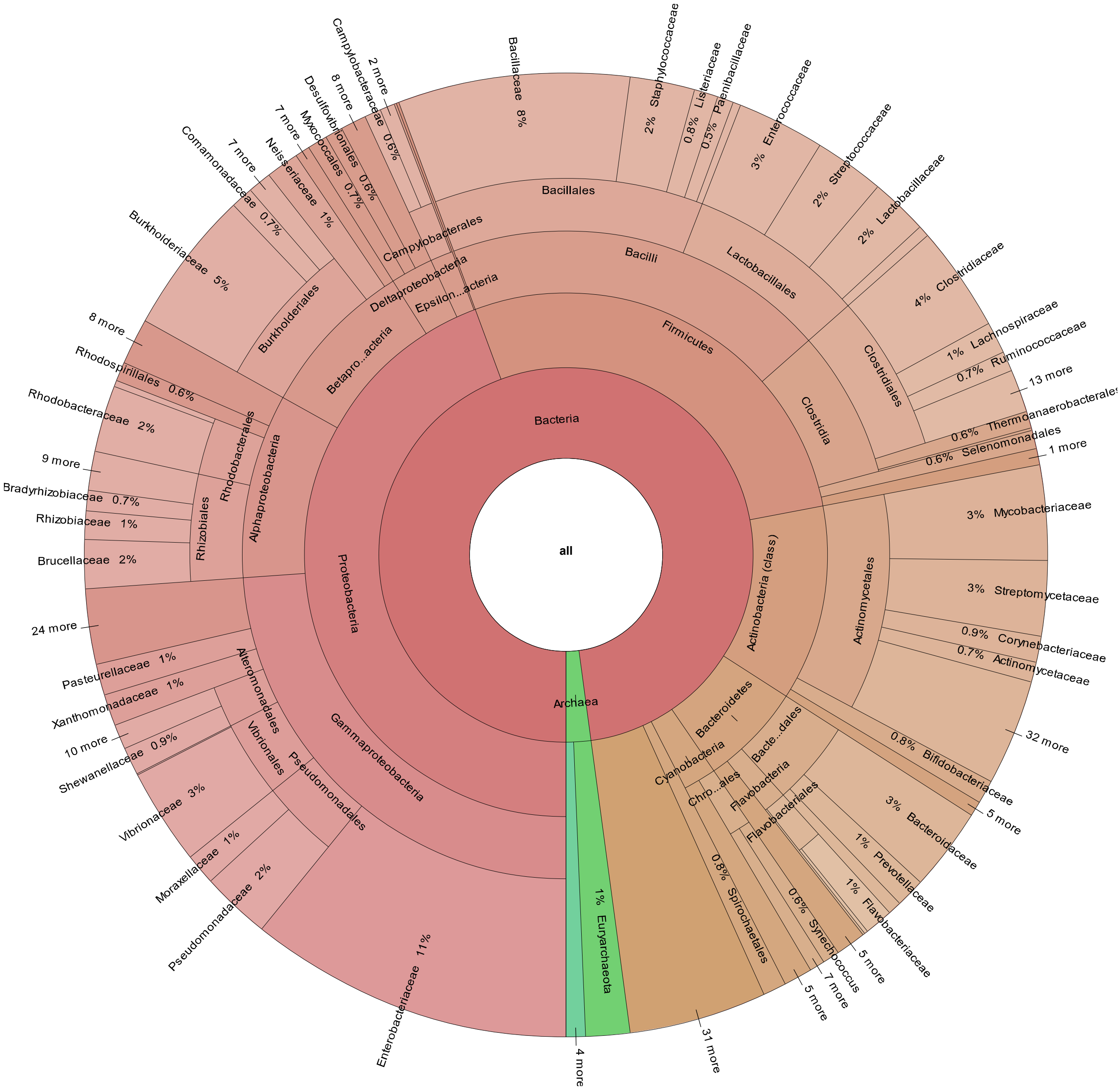

Taxonomic composition down to family level of the microbial (bacteria, archaea and viruses) portion of the *RefSeq47* sequence data collection using Krona (Ondov et *al.*, 2011). An interactive version can be found in the supplementary files (RefSeq47.krona.html). Abundance is measured in terms of accumulated sequence lengths per clade.

Supplementary Figure S5: Taxonomic composition of 16S genes extracted from *RefSeq47*

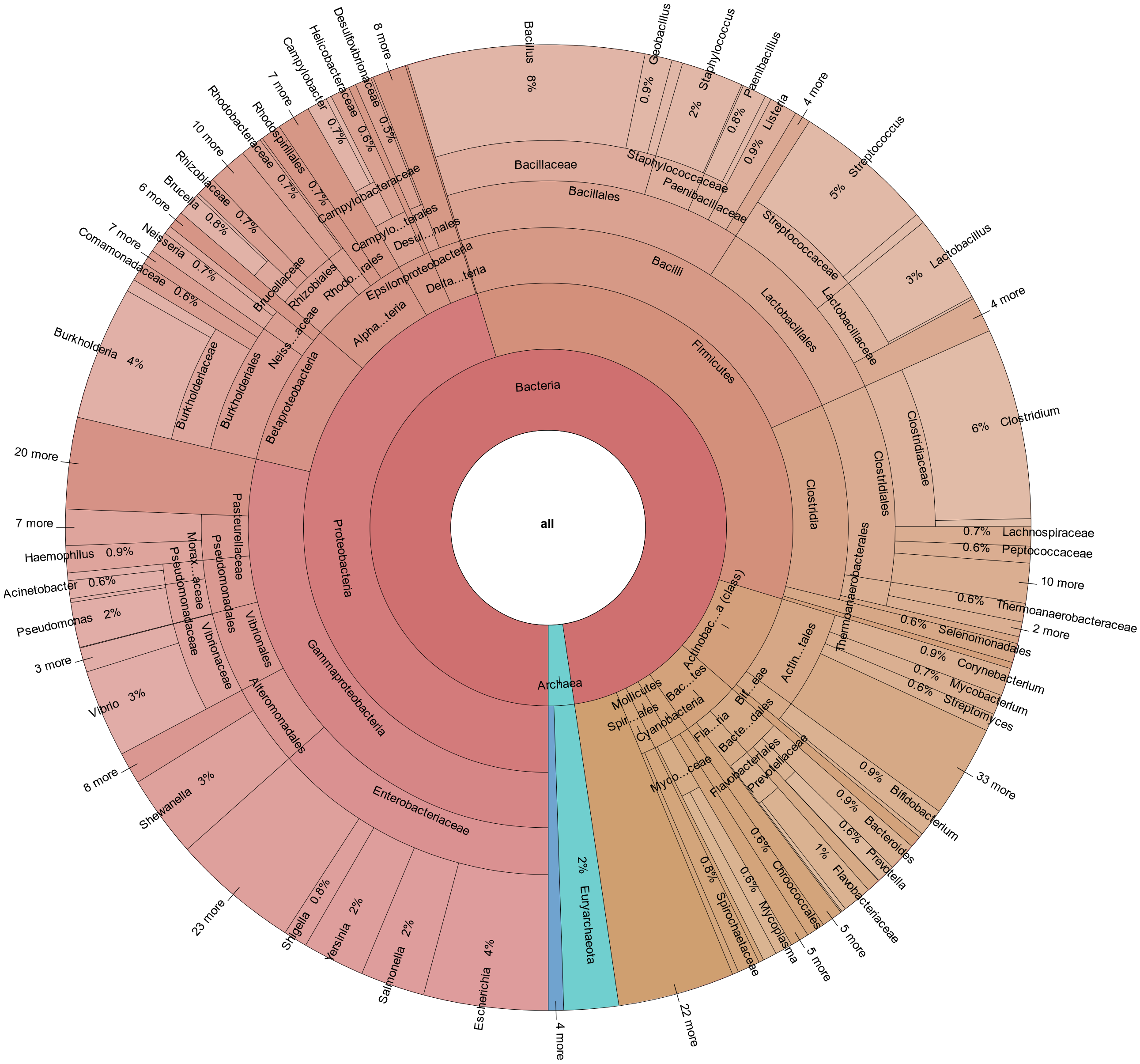

Taxonomic composition down to genus level of the 16S benchmark dataset using Krona (Ondov et *al*., 2011). The dataset was simulated by extracting every annotated 16S gene in *RefSeq47* which was at least 1000 bp long. An interactive version can be found in the supplementary files (refseq-16S.krona.html). Abundance is measured as the number of 16S genes.

(a) summary scenario

rank	depth	true (sequences)	false (sequences)	unknown (sequences)	macro precision α=0.99	stdev	pred. bins	macro recall	stdev	real bins	sum true (sequences)	sum false (sequences)	overall prec.	description
unassigned	0	37391.6	0.0	0	100.0	0.0	1	100.0	0.0	1	101937.3	427.3	99.6	root+superkingdom
superkingdom	1	32272.9	427.3	0	99.2	0.0	1	26.4	26.7	3				
phylum	2	4563.7	2340.3	0	83.0	10.2	11	9.3	8.5	32	8977.7	4995.4	64.2	phylum+class+order
class	3	2164.1	1120.1	0	82.0	13.1	23	8.9	7.6	52				
order	4	2249.9	1535.0	0	86.5	11.1	52	7.8	7.2	110				
family	5	2859.3	591.9	0	85.1	14.7	98	5.8	6.8	240	13520.1	2415.0	84.8	family+genus+species
genus	6	7852.3	1275.7	0	87.3	17.6	202	3.5	5.6	656				
species	7	2808.6	547.4	0	74.0	34.8	431	1.0	2.6	1697				
avg/sum	2.4	54770.7	7837.7	0	85.3	14.5	116.9	8.9	9.3	398.6			87.5	all but unassigned
avg/sum	1.5	92162.3	7837.7	0	87.1	12.7	102.4	20.3	8.1	348.9			92.2	all with unassigned

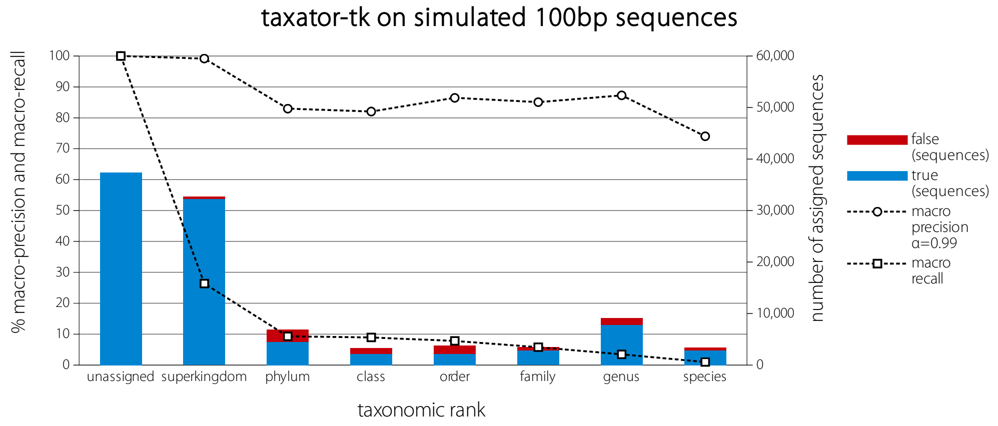

Supplementary Figure S6 - Simulated 100 bp sequence assignment with taxator-tk

(b) all reference scenario

rank	depth	true (sequences)	false (sequences)	unknown (sequences)	macro precision α=0.99	stdev	pred. bins	macro recall	stdev	real bins	sum true (sequences)	sum false (sequences)	overall prec.	description
unassigned	0	10662	0	0	100.0	0.0	1	100.0	0.0	1	47620	0	100.0	root+superkingdom
superkingdom	1	18479	0	0	100.0	0.0	2	48.0	37.0	3				
phylum	2	4362	0	0	100.0	0.0	12	35.2	28.1	32	11598	0	100.0	phylum+class+order
class	3	2607	0	0	100.0	0.0	24	35.7	27.1	52				
order	4	4629	0	0	100.0	0.0	54	33.9	28.2	110				
family	5	8015	0	0	100.0	0.0	104	27.8	29.2	240	59261	0	100.0	family+genus+species
genus	6	31586	0	0	100.0	0.0	211	19.2	28.2	656				
species	7	19660	0	0	100.0	0.0	365	6.9	18.2	1697				
avg/sum	4.1	89338	0	0	100.0	0.0	110.3	29.5	28.0	398.6			100.0	all but unassigned
avg/sum	3.5	100000	0	0	100.0	0.0	96.6	38.4	24.5	348.9			100.0	all with unassigned

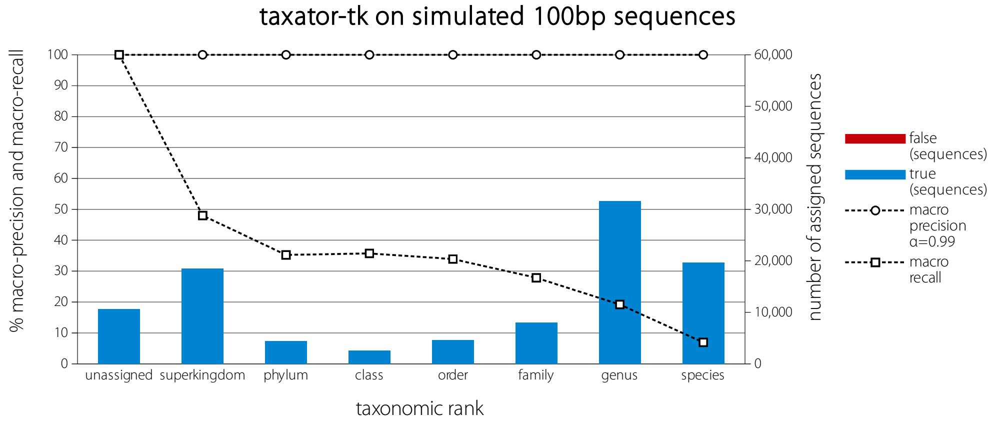

Supplementary Figure S6 - Simulated 100 bp sequence assignment with taxator-tk

(c) new species scenario

rank	depth	true (sequences)	false (sequences)	unknown (sequences)	macro precision α=0.99	stdev	pred. bins	macro recall	stdev	real bins	sum true (sequences)	sum false (sequences)	overall prec.	description
unassigned	0	22319	0	0	100.0	0.0	1	100.0	0.0	1	76901	252	99.7	root+superkingdom
superkingdom	1	27291	252	0	99.6	0.0	1	35.1	32.5	3				
phylum	2	5362	746	0	97.2	1.8	10	17.5	18.3	32	13213	1541	89.6	phylum+class+order
class	3	3240	327	0	97.2	2.9	22	18.1	19.1	52				
order	4	4611	468	0	97.3	3.3	45	15.4	18.5	110				
family	5	7973	255	0	96.1	6.7	75	10.9	18.3	240	31353	4031	88.6	family+genus+species
genus	6	23380	776	0	90.4	21.6	100	5.0	14.2	656				
species	7	0	3000	0	0.0	0.0	217	0.0	0.0	1697				
avg/sum	3.4	71857	5824	0	82.5	5.2	67.1	14.6	17.3	398.6			92.5	all but unassigned
avg/sum	2.6	94176	5824	0	84.7	4.5	58.9	25.3	15.1	348.9			94.2	all with unassigned

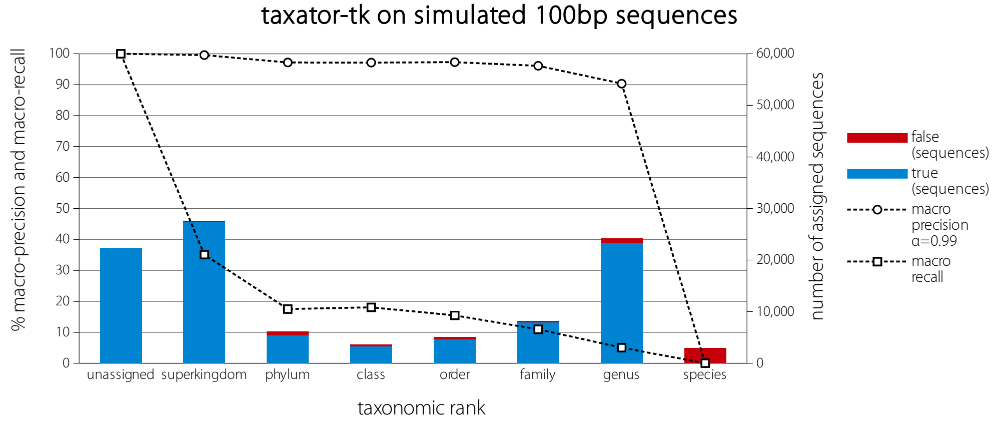

Supplementary Figure S6 - Simulated 100 bp sequence assignment with taxator-tk

(d) new genus scenario

rank	depth	true (sequences)	false (sequences)	unknown (sequences)	macro precision α=0.99	stdev	pred. bins	macro recall	stdev	real bins	sum true (sequences)	sum false (sequences)	overall prec.	description
unassigned	0	34909	0	0	100.0	0.0	1	100.0	0.0	1	110459	343	99.7	root+superkingdom
superkingdom	1	37775	343	0	99.3	0.0	1	27.2	28.0	3				
phylum	2	6814	1406	0	82.6	22.0	8	6.9	9.4	32	14566	3057	82.7	phylum+class+order
class	3	3906	689	0	82.0	17.8	19	6.1	7.9	52				
order	4	3846	962	0	80.6	17.2	44	4.4	7.5	110				
family	5	4027	657	0	49.4	39.3	77	1.7	5.3	240	4027	5323	43.1	family+genus+species
genus	6	0	4422	0	0.0	0.0	193	0.0	0.0	656				
species	7	0	244	0	0.0	0.0	103	0.0	0.0	1697				
avg/sum	2.1	56368	8723	0	56.3	13.8	63.6	6.6	8.3	398.6			86.6	all but unassigned
avg/sum	1.4	91277	8723	0	61.7	12.0	55.8	18.3	7.3	348.9			91.3	all with unassigned

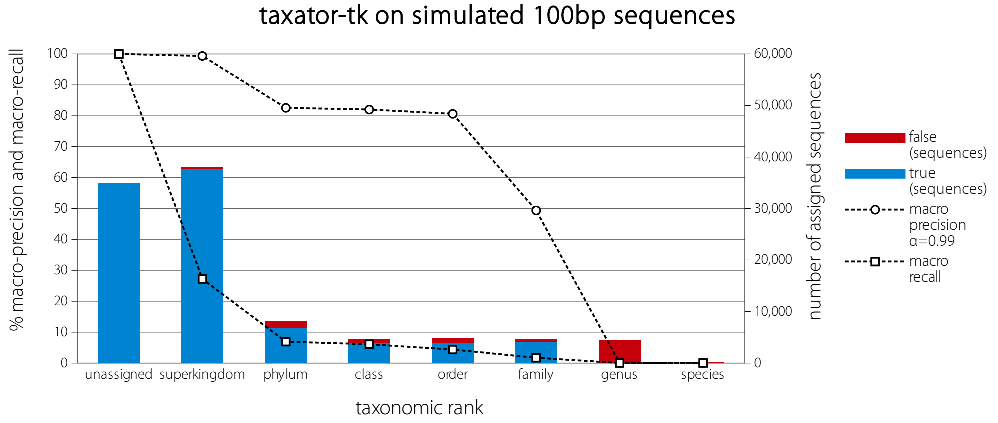

Supplementary Figure S6 - Simulated 100 bp sequence assignment with taxator-tk **(e) new family scenario**

rank	depth	true (sequences)	false (sequences)	unknown (sequences)	macro precision α=0.99	stdev	pred. bins	macro recall	stdev	real bins	sum true (sequences)	sum false (sequences)	overall prec.	description
unassigned	0	40215	0	0	100.0	0.0	1	100.0	0.0	1	120367	525	99.6	root+superkingdom
superkingdom	1	40076	525	0	98.9	0.0	1	22.2	27.0	3				
phylum	2	6632	1627	0	80.7	7.2	6	2.9	5.6	32				
class	3	3425	904	0	59.6	27.6	14	2.2	3.9	52	12720	3840	76.8	phylum+class+order
order	4	2663	1309	0	31.9	33.3	43	0.9	2.5	110				
family	5	0	1045	0	0.0	0.0	120	0.0	0.0	240				
genus	6	0	1413	0	0.0	0.0	133	0.0	0.0	656	0	2624	0.0	family+genus+species
species	7	0	166	0	0.0	0.0	80	0.0	0.0	1697				
avg/sum	1.7	52796	6989	0	38.7	9.7	56.7	4.0	5.6	398.6			88.3	all but unassigned
avg/sum	1.0	93011	6989	0	46.4	8.5	49.8	16.0	4.9	348.9			93.0	all with unassigned

taxator-tk on simulated 100bp sequences

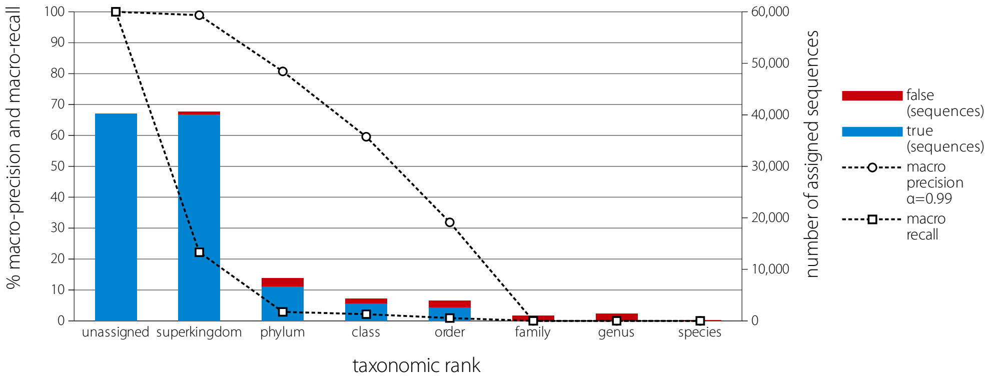

Supplementary Figure S6 - Simulated 100 bp sequence assignment with taxator-tk **(f) new order scenario**

rank	depth	true (sequences)	false (sequences)	unknown (sequences)	macro precision α=0.99	stdev	pred. bins	macro recall	stdev	real bins	sum true (sequences)	sum false (sequences)	overall prec.	description
unassigned	0	44037	0	0	100.0	0.0	1	100.0	0.0	1	121439	563	99.5	root+superkingdom
superkingdom	1	38701	563	0	98.8	0.0	1	20.3	25.5	3				
phylum	2	5817	3314	0	60.2	21.5	6	1.8	4.0	32				
class	3	1971	1414	0	23.3	23.8	18	0.6	1.5	52	7788	6917	53.0	phylum+class+order
order	4	0	2189	0	0.0	0.0	49	0.0	0.0	110				
family	5	0	961	0	0.0	0.0	106	0.0	0.0	240				
genus	6	0	873	0	0.0	0.0	118	0.0	0.0	656	0	1994	0.0	family+genus+species
species	7	0	160	0	0.0	0.0	72	0.0	0.0	1697				
avg/sum	1.5	46489	9474	0	26.0	6.5	52.9	3.2	4.4	398.6			83.1	all but unassigned
avg/sum	0.9	90526	9474	0	35.3	5.7	46.4	15.3	3.9	348.9			90.5	all with unassigned

taxator-tk on simulated 100bp sequences

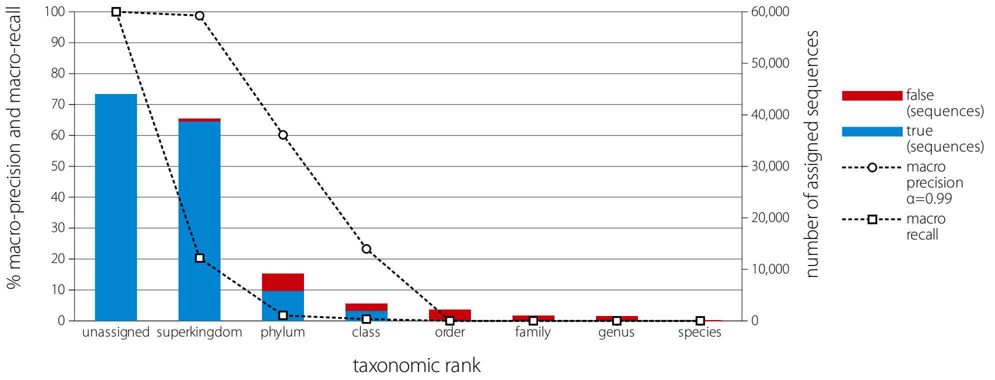

Supplementary Figure S6 - Simulated 100 bp sequence assignment with taxator-tk **(g) new class scenario**

rank	depth	true (sequences)	false (sequences)	unknown (sequences)	macro precision α=0.99	stdev	pred. bins	macro recall	stdev	real bins	sum true (sequences)	sum false (sequences)	overall prec.	description
unassigned	0	50150	0	0	100.0	0.0	1	100.0	0.0	1	122726	579	99.5	root+superkingdom
superkingdom	1	36288	579	0	98.6	0.0	1	17.9	22.8	3				
phylum	2	2959	4203	0	27.7	24.4	7	0.7	2.0	32				
class	3	0	2365	0	0.0	0.0	20	0.0	0.0	52	2959	8462	25.9	phylum+class+order
order	4	0	1894	0	0.0	0.0	49	0.0	0.0	110				
family	5	0	692	0	0.0	0.0	100	0.0	0.0	240				
genus	6	0	742	0	0.0	0.0	109	0.0	0.0	656	0	1562	0.0	family+genus+species
species	7	0	128	0	0.0	0.0	63	0.0	0.0	1697				
avg/sum	1.5	39247	10603	0	18.0	3.5	49.9	2.7	3.5	398.6			78.7	all but unassigned
avg/sum	0.7	89397	10603	0	28.3	3.0	43.8	14.8	3.1	348.9			89.4	all with unassigned

taxator-tk on simulated 100bp sequences

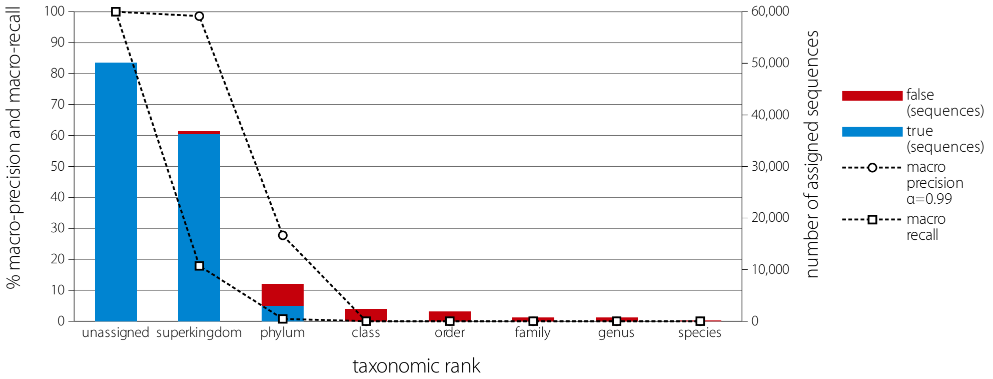

Supplementary Figure S6 - Simulated 100 bp sequence assignment with taxator-tk **(h) new phylum scenario**

rank	depth	true (sequences)	false (sequences)	unknown (sequences)	macro precision α=0.99	stdev	pred. bins	macro recall	stdev	real bins	sum true (sequences)	sum false (sequences)	overall prec.	description
unassigned	0	59449	0	0	100.0	0.0	1	100.0	0.0	1	114049	729	99.4	root+superkingdom
superkingdom	1	27300	729	0	97.9	0.0	1	14.0	18.6	3				
phylum	2	0	5086	0	0.0	0.0	11	0.0	0.0	32				
class	3	0	2142	0	0.0	0.0	20	0.0	0.0	52	0	11151	0.0	phylum+class+order
order	4	0	3923	0	0.0	0.0	44	0.0	0.0	110				
family	5	0	533	0	0.0	0.0	98	0.0	0.0	240				
genus	6	0	704	0	0.0	0.0	105	0.0	0.0	656	0	1371	0.0	family+genus+species
species	7	0	134	0	0.0	0.0	60	0.0	0.0	1697				
avg/sum	1.7	27300	13251	0	14.0	0.0	48.4	2.0	2.7	398.6			67.3	all but unassigned
avg/sum	0.7	86749	13251	0	24.7	0.0	42.5	14.3	2.3	348.9			86.7	all with unassigned

taxator-tk on simulated 100bp sequences

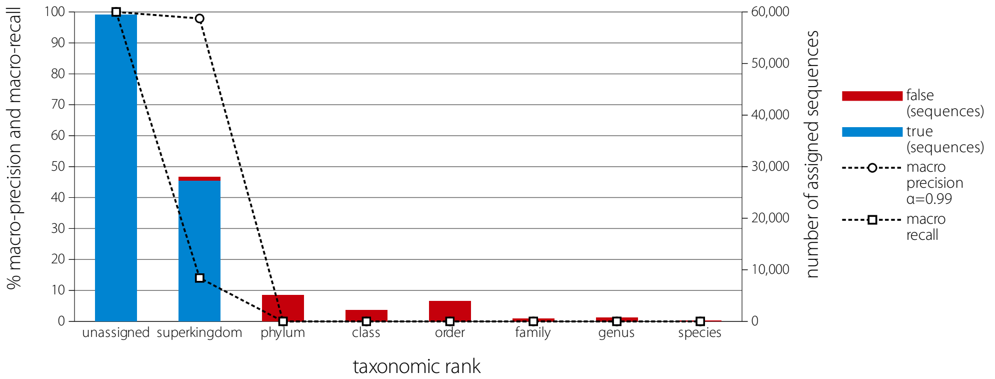

Supplementary Figure S7 - Simulated 500 bp sequence assignment with taxator-tk **(a) summary scenario**

rank	depth	true (sequences)	false (sequences)	unknown (sequences)	macro precision α=0.99	stdev	pred. bins	macro recall	stdev	real bins	sum true (sequences)	sum false (sequences)	overall prec.	description
unassigned	0	20001.1	0.0	0	100.0	0.0	1	100.0	0.0	1	99200.0	582.0	99.4	root+superkingdom
superkingdom	1	39599.4	582.0	0	99.1	0.0	1	53.6	26.8	2				
phylum	2	7862.7	3532.7	0	84.1	12.4	12	13.2	11.4	32				
class	3	3756.1	1555.3	0	81.8	14.8	24	12.1	9.4	52	15065.4	7226.3	67.6	phylum+class+order
order	4	3446.6	2138.3	0	85.1	13.1	56	10.2	8.6	110				
family	5	3162.4	702.6	0	84.6	17.2	104	7.1	7.8	240				
genus	6	7880.9	1428.4	0	87.6	19.3	212	4.2	6.3	656	14666.0	2859.7	83.7	family+genus+species
species	7	3622.7	728.7	0	76.5	34.0	480	1.4	3.4	1693				
avg/sum	2.3	69330.9	10668.0	0	85.6	15.8	127.0	14.5	10.5	397.9			86.7	all but unassigned
avg/sum	1.8	89332.0	10668.0	0	87.4	13.8	111.3	25.2	9.2	348.3			89.3	all with unassigned

taxator-tk on simulated 500bp sequences

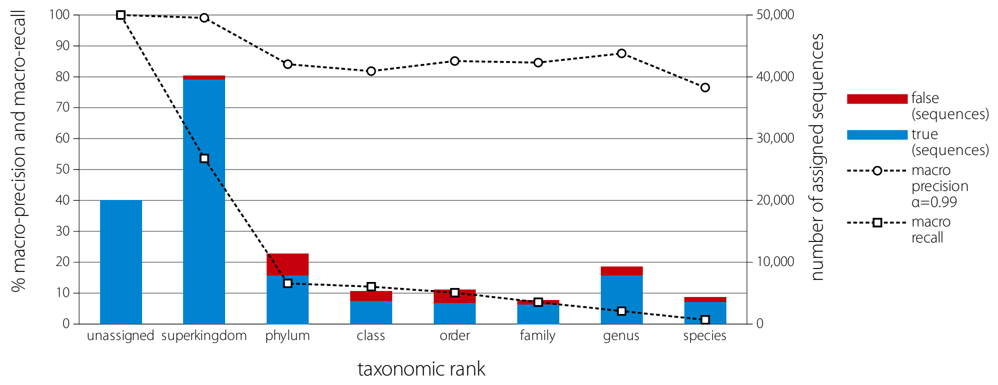

Supplementary Figure S7 - Simulated 500 bp sequence assignment with taxator-tk **(b) all reference scenario**

rank	depth	true (sequences)	false (sequences)	unknown (sequences)	macro precision α=0.99	stdev	pred. bins	macro recall	stdev	real bins	sum true (sequences)	sum false (sequences)	overall prec.	description
unassigned	0	7999	0	0	100.0	0.0	1	100.0	0.0	1	42883	0	100.0	root+superkingdom
superkingdom	1	17442	0	0	100.0	0.0	2	81.8	10.7	2				
phylum	2	4415	0	0	100.0	0.0	14	43.0	30.7	32				
class	3	2699	0	0	100.0	0.0	27	43.7	29.3	52	11750	0	100.0	phylum+class+order
order	4	4636	0	0	100.0	0.0	59	40.6	30.9	110				
family	5	7889	0	0	100.0	0.0	109	32.6	31.8	240				
genus	6	29561	0	0	100.0	0.0	221	23.0	32.0	656	62809	0	100.0	family+genus+species
species	7	25359	0	0	100.0	0.0	408	9.5	23.5	1693				
avg/sum	4.0	92001	0	0	100.0	0.0	120.0	39.2	27.0	397.9			100.0	all but unassigned
avg/sum	3.6	100000	0	0	100.0	0.0	105.1	46.8	23.6	348.3			100.0	all with unassigned

taxator-tk on simulated 500bp sequences

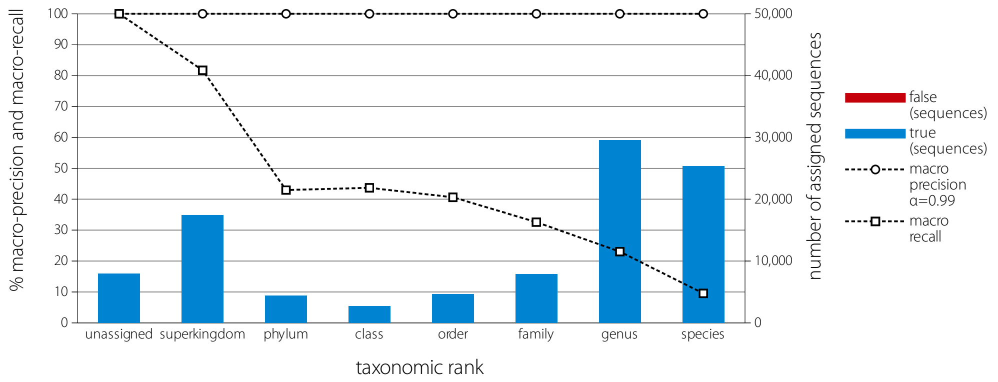

Supplementary Figure S7 - Simulated 500 bp sequence assignment with taxator-tk **(c) new species scenario**

rank	depth	true (sequences)	false (sequences)	unknown (sequences)	macro precision α=0.99	stdev	pred. bins	macro recall	stdev	real bins	sum true (sequences)	sum false (sequences)	overall prec.	description
unassigned	0	10520	0	0	100.0	0.0	1	100.0	0.0	1	67276	224	99.7	root+superkingdom
superkingdom	1	28378	224	0	99.2	0.5	2	68.6	21.6	2				
phylum	2	8027	773	0	98.4	1.2	11	24.7	23.6	32				
class	3	4991	337	0	97.8	2.6	23	24.9	23.3	52	19494	1591	92.5	phylum+class+order
order	4	6476	481	0	97.7	2.8	50	21.0	22.3	110				
family	5	9009	253	0	96.6	6.6	79	14.2	21.7	240				
genus	6	25605	910	0	90.8	21.1	107	6.3	16.4	656	34614	5179	87.0	family+genus+species
species	7	0	4016	0	0.0	0.0	237	0.0	0.0	1693				
avg/sum	3.5	82486	6994	0	82.9	5.0	72.7	22.8	18.4	397.9			92.2	all but unassigned
avg/sum	3.1	93006	6994	0	85.1	4.3	63.8	32.5	16.1	348.3			93.0	all with unassigned

taxator-tk on simulated 500bp sequences

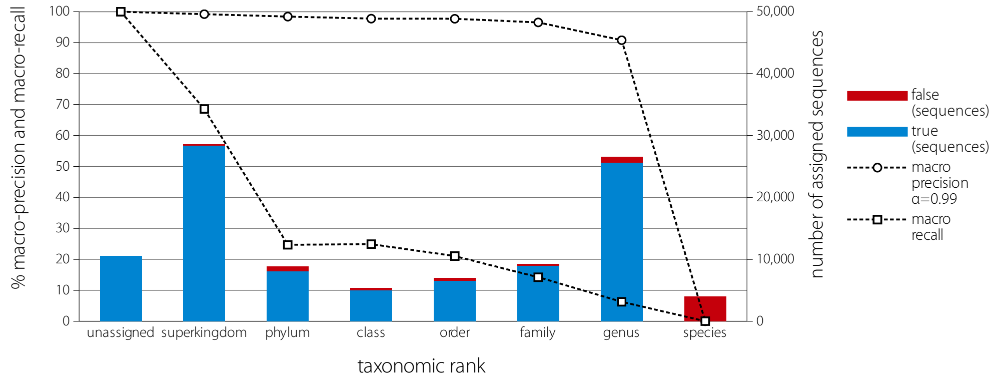

Supplementary Figure S7 - Simulated 500 bp sequence assignment with taxator-tk **(d) new genus scenario**

rank	depth	true (sequences)	false (sequences)	unknown (sequences)	macro precision α=0.99	stdev	pred. bins	macro recall	stdev	real bins	sum true (sequences)	sum false (sequences)	overall prec.	description
unassigned	0	13884	0	0	100.0	0.0	1	100.0	0.0	1	100784	357	99.6	root+superkingdom
superkingdom	1	43450	357	0	99.5	0.0	1	60.2	26.6	2				
phylum	2	12278	1548	0	95.9	2.9	9	13.4	15.2	32				
class	3	7723	760	0	87.2	13.6	21	10.9	12.4	52	27611	3340	89.2	phylum+class+order
order	4	7610	1032	0	86.3	12.4	47	7.8	11.2	110				
family	5	5239	761	0	52.7	40.4	81	2.8	7.9	240				
genus	6	0	5064	0	0.0	0.0	136	0.0	0.0	656	5239	6119	46.1	family+genus+species
species	7	0	294	0	0.0	0.0	105	0.0	0.0	1693				
avg/sum	2.2	76300	9816	0	60.2	9.9	57.1	13.6	10.5	397.9			88.6	all but unassigned
avg/sum	1.9	90184	9816	0	65.2	8.7	50.1	24.4	9.2	348.3			90.2	all with unassigned

taxator-tk on simulated 500bp sequences

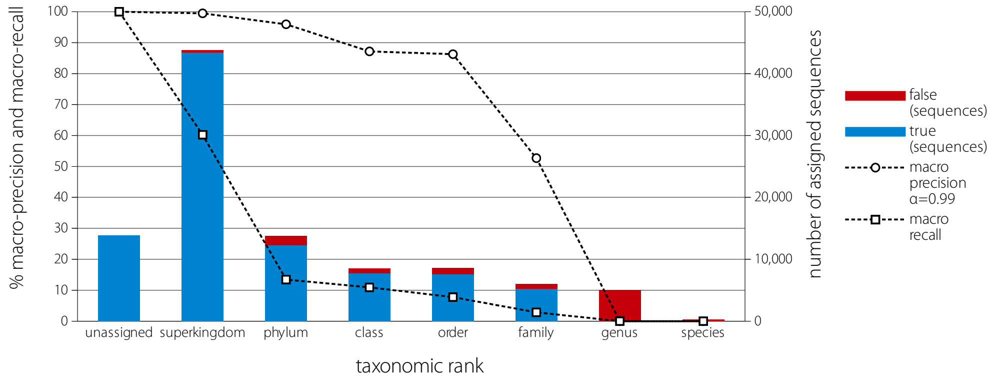

Supplementary Figure S7 - Simulated 500 bp sequence assignment with taxator-tk **(e) new family scenario**

rank	depth	true (sequences)	false (sequences)	unknown (sequences)	macro precision α=0.99	stdev	pred. bins	macro recall	stdev	real bins	sum true (sequences)	sum false (sequences)	overall prec.	description
unassigned	0	18731	0	0	100.0	0.0	1	100.0	0.0	1	114197	702	99.4	root+superkingdom
superkingdom	1	47733	702	0	98.9	0.0	1	47.8	34.0	2				
phylum	2	12581	2006	0	88.9	4.2	6	5.9	10.5	32				
class	3	7177	1099	0	64.4	31.5	16	4.0	6.7	52	25162	4675	84.3	phylum+class+order
order	4	5404	1570	0	40.0	36.6	42	1.6	4.2	110				
family	5	0	1251	0	0.0	0.0	103	0.0	0.0	240				
genus	6	0	1492	0	0.0	0.0	122	0.0	0.0	656	0	2997	0.0	family+genus+species
species	7	0	254	0	0.0	0.0	82	0.0	0.0	1693				
avg/sum	1.8	72895	8374	0	41.8	10.3	53.1	8.5	7.9	397.9			89.7	all but unassigned
avg/sum	1.5	91626	8374	0	49.0	9.0	46.6	19.9	6.9	348.3			91.6	all with unassigned

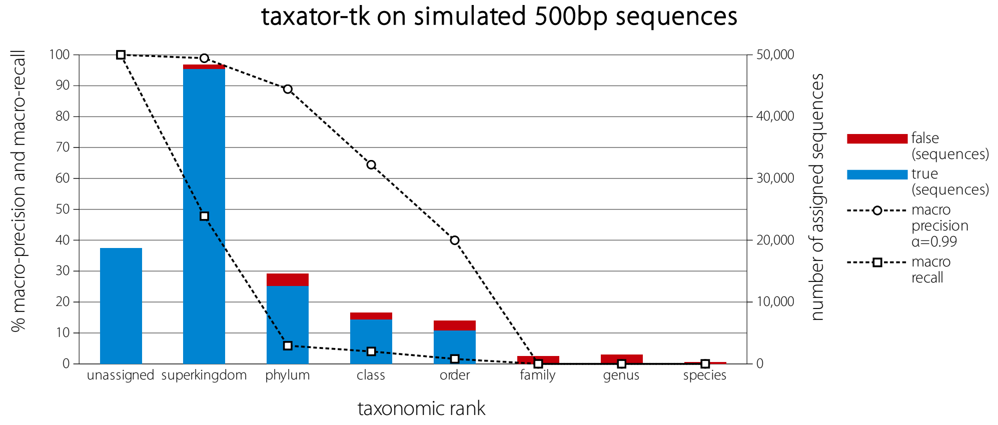

Supplementary Figure S7 - Simulated 500 bp sequence assignment with taxator-tk **(f) new order scenario**

rank	depth	true (sequences)	false (sequences)	unknown (sequences)	macro precision α=0.99	stdev	pred. bins	macro recall	stdev	real bins	sum true (sequences)	sum false (sequences)	overall prec.	description
unassigned	0	21881	0	0	100.0	0.0	1	100.0	0.0	1	121329	771	99.4	root+superkingdom
superkingdom	1	49724	771	0	98.8	0.0	1	44.4	34.2	2				
phylum	2	11475	4991	0	71.7	17.0	6	3.6	7.8	32				
class	3	3703	1888	0	31.9	29.3	18	1.1	2.5	52	15178	10362	59.4	phylum+class+order
order	4	0	3483	0	0.0	0.0	56	0.0	0.0	110				
family	5	0	1003	0	0.0	0.0	106	0.0	0.0	240				
genus	6	0	868	0	0.0	0.0	110	0.0	0.0	656	0	2084	0.0	family+genus+species
species	7	0	213	0	0.0	0.0	63	0.0	0.0	1693				
avg/sum	1.6	64902	13217	0	28.9	6.6	51.4	7.0	6.4	397.9			83.1	all but unassigned
avg/sum	1.2	86783	13217	0	37.8	5.8	45.1	18.6	5.6	348.3			86.8	all with unassigned

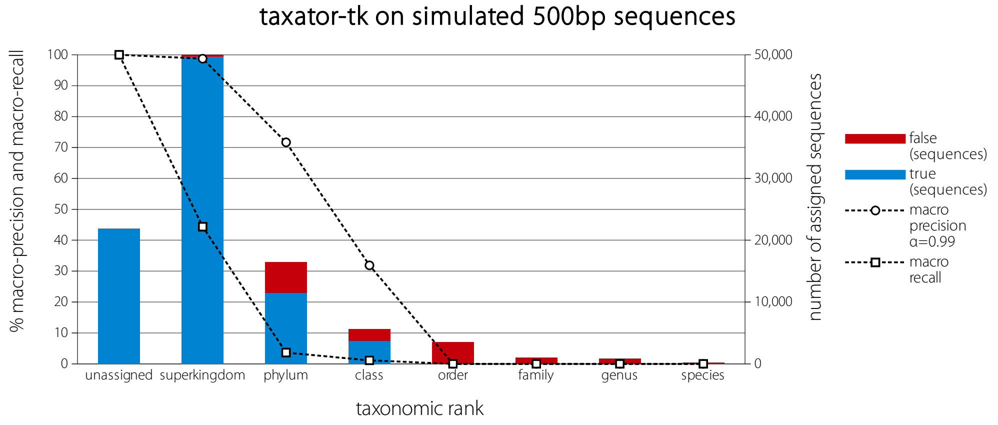

Supplementary Figure S7 - Simulated 500 bp sequence assignment with taxator-tk **(g) new class scenario**

rank	depth	true (sequences)	false (sequences)	unknown (sequences)	macro precision α=0.99	stdev	pred. bins	macro recall	stdev	real bins	sum true (sequences)	sum false (sequences)	overall prec.	description
unassigned	0	28770	0	0	100.0	0.0	1	100.0	0.0	1	127418	838	99.3	root+superkingdom
superkingdom	1	49324	838	0	98.5	0.0	1	39.9	31.6	2				
phylum	2	6263	6679	0	34.0	28.9	7	1.5	3.8	32				
class	3	0	3676	0	0.0	0.0	21	0.0	0.0	52	6263	12967	32.6	phylum+class+order
order	4	0	2612	0	0.0	0.0	52	0.0	0.0	110				
family	5	0	852	0	0.0	0.0	108	0.0	0.0	240				
genus	6	0	834	0	0.0	0.0	103	0.0	0.0	656	0	1838	0.0	family+genus+species
species	7	0	152	0	0.0	0.0	56	0.0	0.0	1693				
avg/sum	1.5	55587	15643	0	18.9	4.1	49.7	5.9	5.1	397.9			78.0	all but unassigned
avg/sum	1.1	84357	15643	0	29.1	3.6	43.6	17.7	4.4	348.3			84.4	all with unassigned

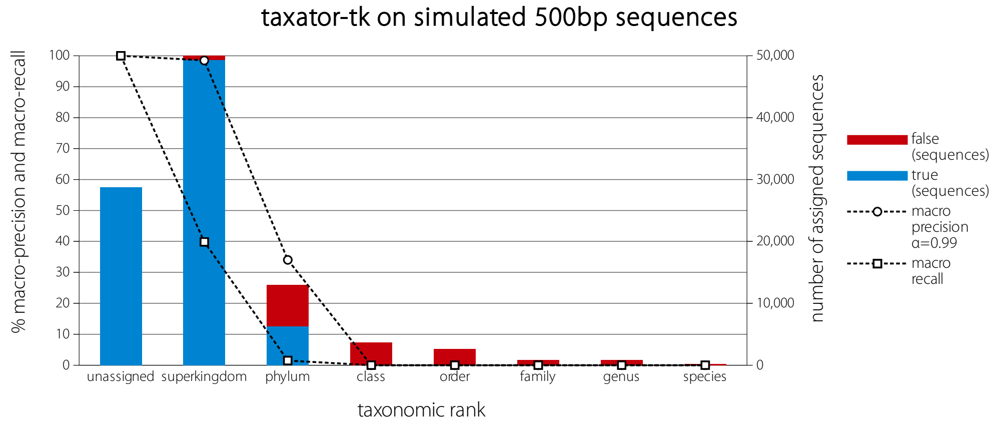

Supplementary Figure S7 - Simulated 500 bp sequence assignment with taxator-tk **(h) new phylum scenario**

rank	depth	true (sequences)	false (sequences)	unknown (sequences)	macro precision α=0.99	stdev	pred. bins	macro recall	stdev	real bins	sum true (sequences)	sum false (sequences)	overall prec.	description
unassigned	0	38223	0	0	100.0	0.0	1	100.0	0.0	1	120513	1182	99.0	root+superkingdom
superkingdom	1	41145	1182	0	97.7	0.0	1	32.6	28.9	2				
phylum	2	0	8732	0	0.0	0.0	11	0.0	0.0	32				
class	3	0	3127	0	0.0	0.0	22	0.0	0.0	52	0	17649	0.0	phylum+class+order
order	4	0	5790	0	0.0	0.0	44	0.0	0.0	110				
family	5	0	798	0	0.0	0.0	106	0.0	0.0	240				
genus	6	0	831	0	0.0	0.0	93	0.0	0.0	656	0	1801	0.0	family+genus+species
species	7	0	172	0	0.0	0.0	45	0.0	0.0	1693				
avg/sum	1.6	41145	20632	0	14.0	0.0	46.0	4.7	4.1	397.9			66.6	all but unassigned
avg/sum	1.0	79368	20632	0	24.7	0.0	40.4	16.6	3.6	348.3			79.4	all with unassigned

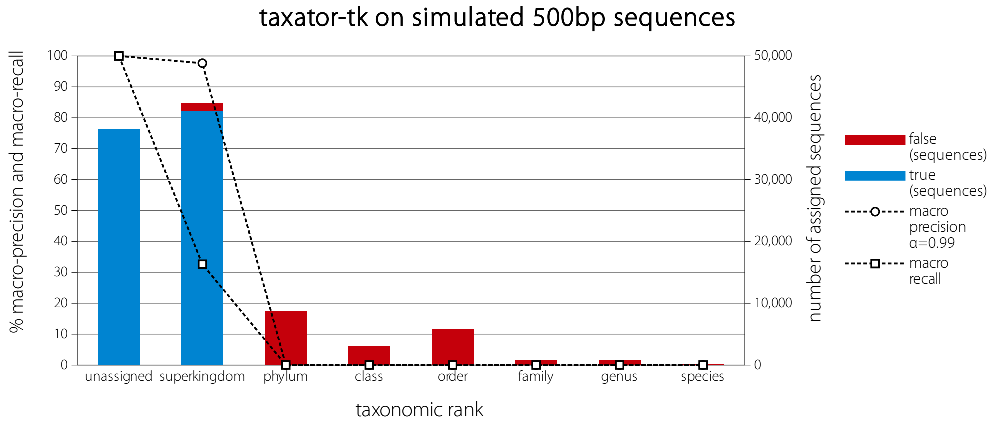

Supplementary Figure S8 - Simulated 1000 bp sequence assignment with taxator-tk **(a) summary scenario**

rank	depth	true (sequences)	false (sequences)	unknown (sequences)	macro precision α=0.99	stdev	pred. bins	macro recall	stdev	real bins	sum true (sequences)	sum false (sequences)	overall prec.	description
unassigned	0	18217.3	0.0	0	100.0	0.0	1	100.0	0.0	1	93809.9	550.7	99.4	root+superkingdom
superkingdom	1	37796.3	550.7	0	99.2	0.0	1	38.1	33.8	3				
phylum	2	9465.1	3300.7	0	87.0	12.2	12	15.2	12.7	32	18677.1	6634.6	73.8	phylum+class+order
class	3	4795.0	1367.1	0	83.2	14.7	25	13.4	10.3	52				
order	4	4417.0	1966.7	0	84.5	15.1	57	10.8	9.2	110				
family	5	3498.1	817.1	0	84.4	17.9	106	7.5	8.1	240	15169.9	2954.1	83.7	family+genus+species
genus	6	7834.3	1397.6	0	86.4	19.6	219	4.3	6.3	653				
species	7	3837.4	739.4	0	77.2	34.2	472	1.5	3.5	1690				
avg/sum	2.4	71643.3	10139.4	0	86.0	16.2	127.4	13.0	12.0	397.1			87.6	all but unassigned
avg/sum	1.9	89860.6	10139.4	0	87.7	14.2	111.6	23.8	10.5	347.6			89.9	all with unassigned

taxator-tk on simulated 1000bp sequences

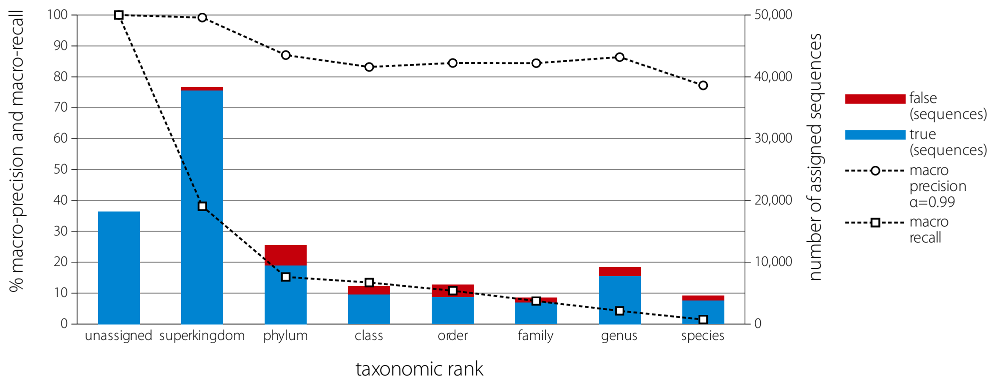

Supplementary Figure S8 - Simulated 1000 bp sequence assignment with taxator-tk **(b) all reference scenario**

rank	depth	true (sequences)	false (sequences)	unknown (sequences)	macro precision α=0.99	stdev	pred. bins	macro recall	stdev	real bins	sum true (sequences)	sum false (sequences)	overall prec.	description
unassigned	0	7256	0	0	100.0	0.0	1	100.0	0.0	1	40990	0	100.0	root+superkingdom
superkingdom	1	16867	0	0	100.0	0.0	2	55.1	39.9	3				
phylum	2	4739	0	0	100.0	0.0	14	45.2	30.5	32	12595	0	100.0	phylum+class+order
class	3	3024	0	0	100.0	0.0	27	44.7	29.7	52				
order	4	4832	0	0	100.0	0.0	59	40.6	31.1	110				
family	5	8132	0	0	100.0	0.0	112	33.1	32.0	240	63282	0	100.0	family+genus+species
genus	6	28288	0	0	100.0	0.0	221	23.3	32.2	653				
species	7	26862	0	0	100.0	0.0	402	10.1	24.8	1690				
avg/sum	4.0	92744	0	0	100.0	0.0	119.6	36.0	31.5	397.1			100.0	all but unassigned
avg/sum	3.6	100000	0	0	100.0	0.0	104.8	44.0	27.5	347.6			100.0	all with unassigned

taxator-tk on simulated 1000bp sequences

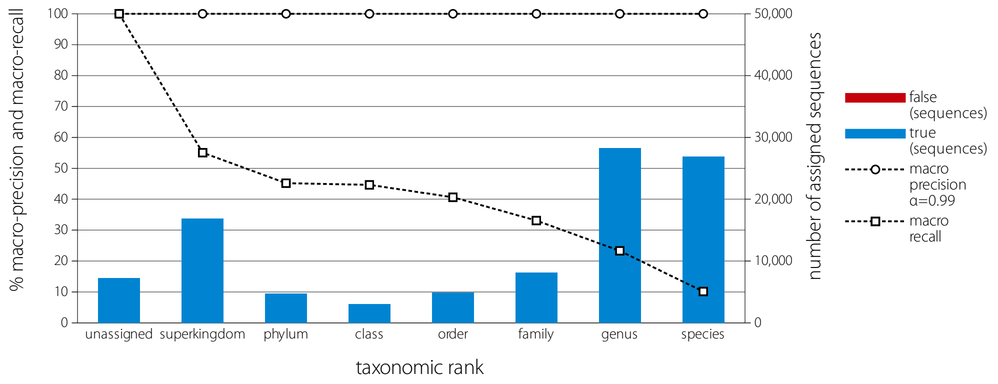

Supplementary Figure S8 - Simulated 1000 bp sequence assignment with taxator-tk **(c) new species scenario**

rank	depth	true (sequences)	false (sequences)	unknown (sequences)	macro precision α=0.99	stdev	pred. bins	macro recall	stdev	real bins	sum true (sequences)	sum false (sequences)	overall prec.	description
unassigned	0	7557	0	0	100.0	0.0	1	100.0	0.0	1	60223	191	99.7	root+superkingdom
superkingdom	1	26333	191	0	99.5	0.2	2	49.7	38.2	3				
phylum	2	9128	523	0	99.2	0.6	12	28.6	25.3	32	23034	1142	95.3	phylum+class+order
class	3	6028	250	0	98.4	2.4	24	28.3	25.2	52				
order	4	7878	369	0	97.8	4.4	52	23.0	23.6	110				
family	5	9803	325	0	96.3	9.0	83	15.4	22.6	240	36355	5388	87.1	family+genus+species
genus	6	26552	898	0	90.5	22.4	107	6.5	16.7	653				
species	7	0	4165	0	0.0	0.0	230	0.0	0.0	1690				
avg/sum	3.5	85722	6721	0	83.1	5.6	72.9	21.6	21.7	397.1			92.7	all but unassigned
avg/sum	3.3	93279	6721	0	85.2	4.9	63.9	31.4	19.0	347.6			93.3	all with unassigned

taxator-tk on simulated 1000bp sequences

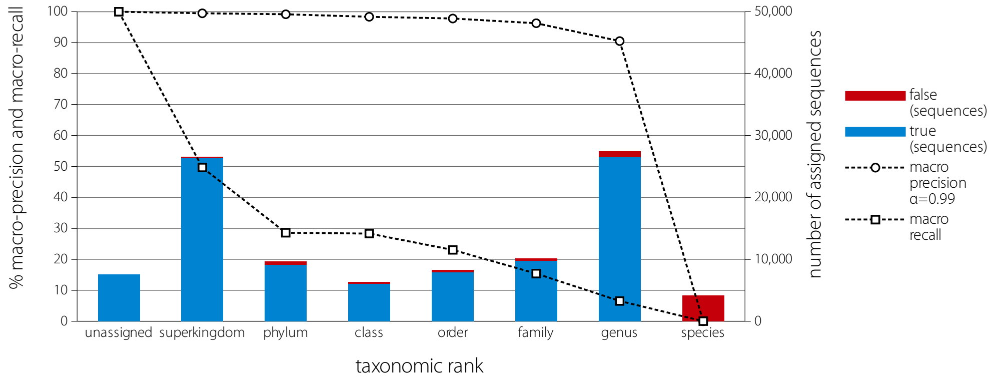

Supplementary Figure S8 - Simulated 1000 bp sequence assignment with taxator-tk **(d) new genus scenario**

rank	depth	true (sequences)	false (sequences)	unknown (sequences)	macro precision α=0.99	stdev	pred. bins	macro recall	stdev	real bins	sum true (sequences)	sum false (sequences)	overall prec.	description
unassigned	0	9974	0	0	100.0	0.0	1	100.0	0.0	1	89246	293	99.7	root+superkingdom
superkingdom	1	39636	293	0	98.8	0.8	2	44.0	37.1	3				
phylum	2	14673	1006	0	94.6	9.8	10	17.7	18.6	32	34900	2418	93.5	phylum+class+order
class	3	9782	538	0	90.4	11.6	22	14.2	15.4	52				
order	4	10445	874	0	90.2	10.8	47	9.7	13.5	110				
family	5	6552	955	0	59.0	39.9	82	3.7	9.4	240	6552	6227	51.3	family+genus+species
genus	6	0	4978	0	0.0	0.0	143	0.0	0.0	653				
species	7	0	294	0	0.0	0.0	94	0.0	0.0	1690				
avg/sum	2.4	81088	8938	0	61.9	10.4	57.1	12.8	13.4	397.1			90.1	all but unassigned
avg/sum	2.2	91062	8938	0	66.6	9.1	50.1	23.7	11.7	347.6			91.1	all with unassigned

taxator-tk on simulated 1000bp sequences

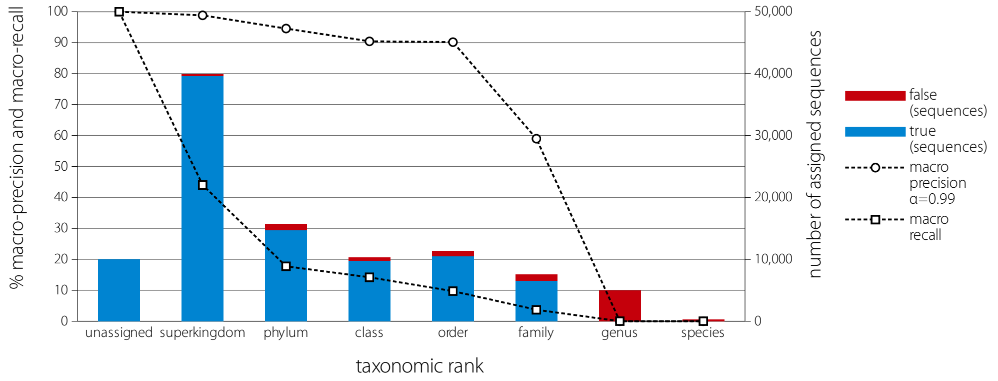

Supplementary Figure S8 - Simulated 1000 bp sequence assignment with taxator-tk **(e) new family scenario**

rank	depth	true (sequences)	false (sequences)	unknown (sequences)	macro precision α=0.99	stdev	pred. bins	macro recall	stdev	real bins	sum true (sequences)	sum false (sequences)	overall prec.	description
unassigned	0	15429	0	0	100.0	0.0	1	100.0	0.0	1	103635	644	99.4	root+superkingdom
superkingdom	1	44103	644	0	99.1	0.0	1	35.3	36.3	3				
phylum	2	15330	1458	0	94.1	2.4	6	8.0	13.7	32				
class	3	9698	849	0	79.4	24.5	15	5.4	9.1	52	32792	3711	89.8	phylum+class+order
order	4	7764	1404	0	45.8	38.6	40	2.1	5.6	110				
family	5	0	1548	0	0.0	0.0	104	0.0	0.0	240				
genus	6	0	1507	0	0.0	0.0	127	0.0	0.0	653	0	3321	0.0	family+genus+species
species	7	0	266	0	0.0	0.0	65	0.0	0.0	1690				
avg/sum	1.9	76895	7676	0	45.5	9.4	51.1	7.3	9.3	397.1			90.9	all but unassigned
avg/sum	1.6	92324	7676	0	52.3	8.2	44.9	18.9	8.1	347.6			92.3	all with unassigned

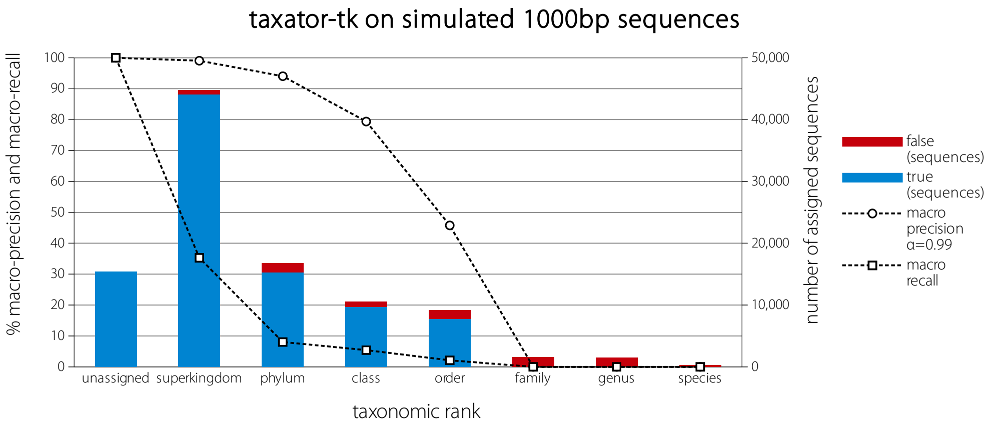

Supplementary Figure S8 - Simulated 1000 bp sequence assignment with taxator-tk **(f) new order scenario**

rank	depth	true (sequences)	false (sequences)	unknown (sequences)	macro precision α=0.99	stdev	pred. bins	macro recall	stdev	real bins	sum true (sequences)	sum false (sequences)	overall prec.	description
unassigned	0	19932	0	0	100.0	0.0	1	100.0	0.0	1	115360	725	99.4	root+superkingdom
superkingdom	1	47714	725	0	98.9	0.0	1	32.4	34.7	3				
phylum	2	14366	4764	0	81.4	11.9	6	5.0	10.2	32				
class	3	5033	1596	0	40.2	33.9	17	1.5	3.5	52	19399	9995	66.0	phylum+class+order
order	4	0	3635	0	0.0	0.0	49	0.0	0.0	110				
family	5	0	1133	0	0.0	0.0	80	0.0	0.0	240				
genus	6	0	883	0	0.0	0.0	98	0.0	0.0	653	0	2235	0.0	family+genus+species
species	7	0	219	0	0.0	0.0	50	0.0	0.0	1690				
avg/sum	1.7	67113	12955	0	31.5	6.5	43.0	5.6	6.9	397.1			83.8	all but unassigned
avg/sum	1.3	87045	12955	0	40.1	5.7	37.8	17.4	6.0	347.6			87.0	all with unassigned

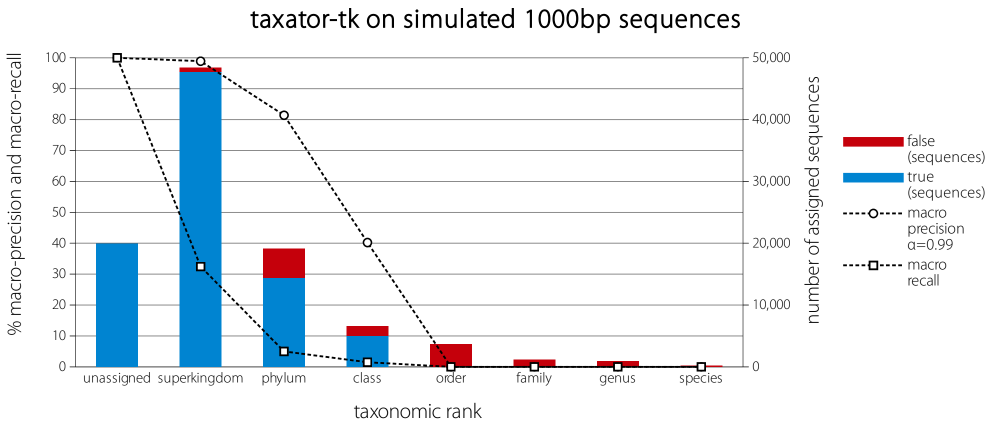

Supplementary Figure S8 - Simulated 1000 bp sequence assignment with taxator-tk **(g) new class scenario**

rank	depth	true (sequences)	false (sequences)	unknown (sequences)	macro precision α=0.99	stdev	pred. bins	macro recall	stdev	real bins	sum true (sequences)	sum false (sequences)	overall prec.	description
unassigned	0	28057	0	0	100.0	0.0	1	100.0	0.0	1	125231	824	99.3	root+superkingdom
superkingdom	1	48587	824	0	98.6	0.0	1	28.5	31.4	3				
phylum	2	8020	6696	0	39.8	34.0	7	2.2	5.1	32				
class	3	0	3649	0	0.0	0.0	22	0.0	0.0	52	8020	12645	38.8	phylum+class+order
order	4	0	2300	0	0.0	0.0	48	0.0	0.0	110				
family	5	0	939	0	0.0	0.0	94	0.0	0.0	240				
genus	6	0	835	0	0.0	0.0	94	0.0	0.0	653	0	1867	0.0	family+genus+species
species	7	0	93	0	0.0	0.0	38	0.0	0.0	1690				
avg/sum	1.5	56607	15336	0	19.8	4.9	43.4	4.4	5.2	397.1			78.7	all but unassigned
avg/sum	1.1	84664	15336	0	29.8	4.2	38.1	16.3	4.6	347.6			84.7	all with unassigned

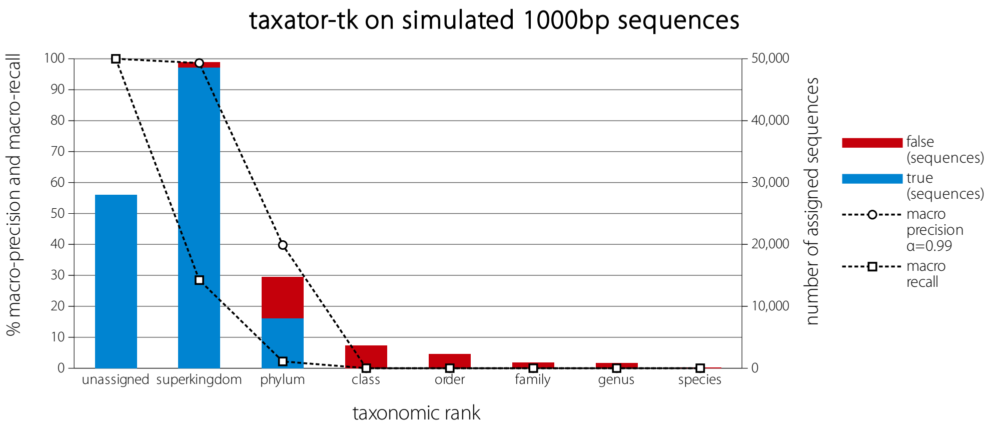

Supplementary Figure S8 - Simulated 1000 bp sequence assignment with taxator-tk **(h) new phylum scenario**

rank	depth	true (sequences)	false (sequences)	unknown (sequences)	macro precision α=0.99	stdev	pred. bins	macro recall	stdev	real bins	sum true (sequences)	sum false (sequences)	overall prec.	description
unassigned	0	39316	0	0	100.0	0.0	1	100.0	0.0	1	121984	1178	99.0	root+superkingdom
superkingdom	1	41334	1178	0	97.7	0.0	1	21.9	27.3	3				
phylum	2	0	8658	0	0.0	0.0	12	0.0	0.0	32				
class	3	0	2688	0	0.0	0.0	21	0.0	0.0	52	0	16531	0.0	phylum+class+order
order	4	0	5185	0	0.0	0.0	44	0.0	0.0	110				
family	5	0	820	0	0.0	0.0	98	0.0	0.0	240				
genus	6	0	682	0	0.0	0.0	99	0.0	0.0	653	0	1641	0.0	family+genus+species
species	7	0	139	0	0.0	0.0	41	0.0	0.0	1690				
avg/sum	1.6	41334	19350	0	14.0	0.0	45.1	3.1	3.9	397.1			68.1	all but unassigned
avg/sum	1.0	80650	19350	0	24.7	0.0	39.6	15.2	3.4	347.6			80.7	all with unassigned

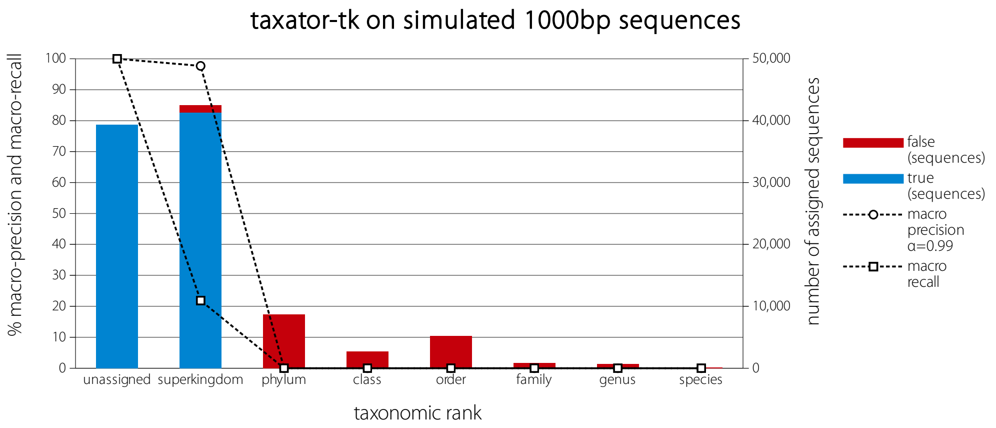

Supplementary Figure S9: Taxonomic composition of microbial *RefSeq54*

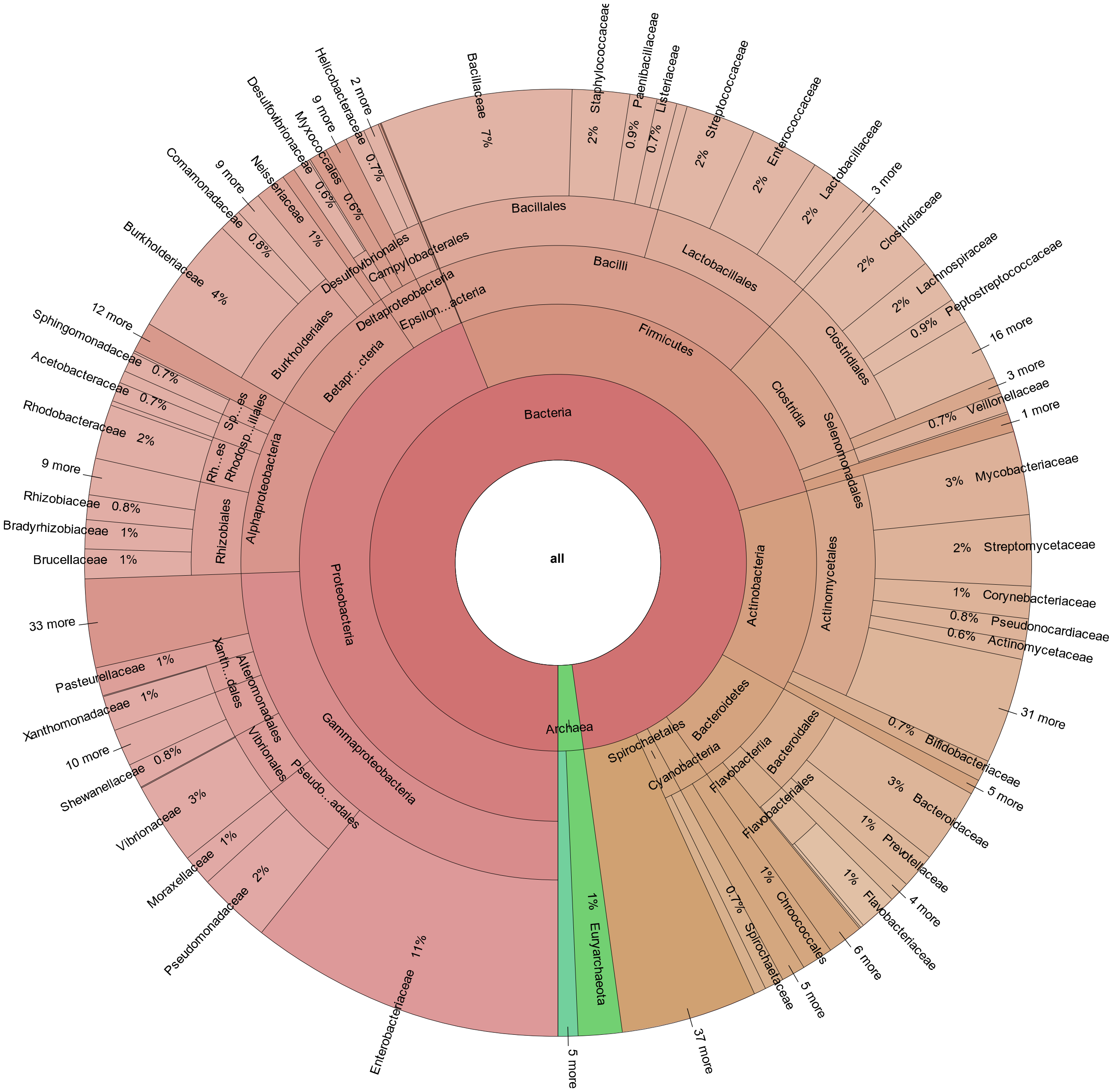

Taxonomic composition down to family level of the microbial (bacteria, archaea and viruses) portion of the *RefSeq54* sequence data collection using Krona (Ondov et *al*., 2011). An interactive version can be found in the supplementary files (RefSeq54.krona.html). Abundance is measured in terms of accumulated sequence lengths per clade.

Supplementary Figure S10: Taxonomic composition of simArt49e

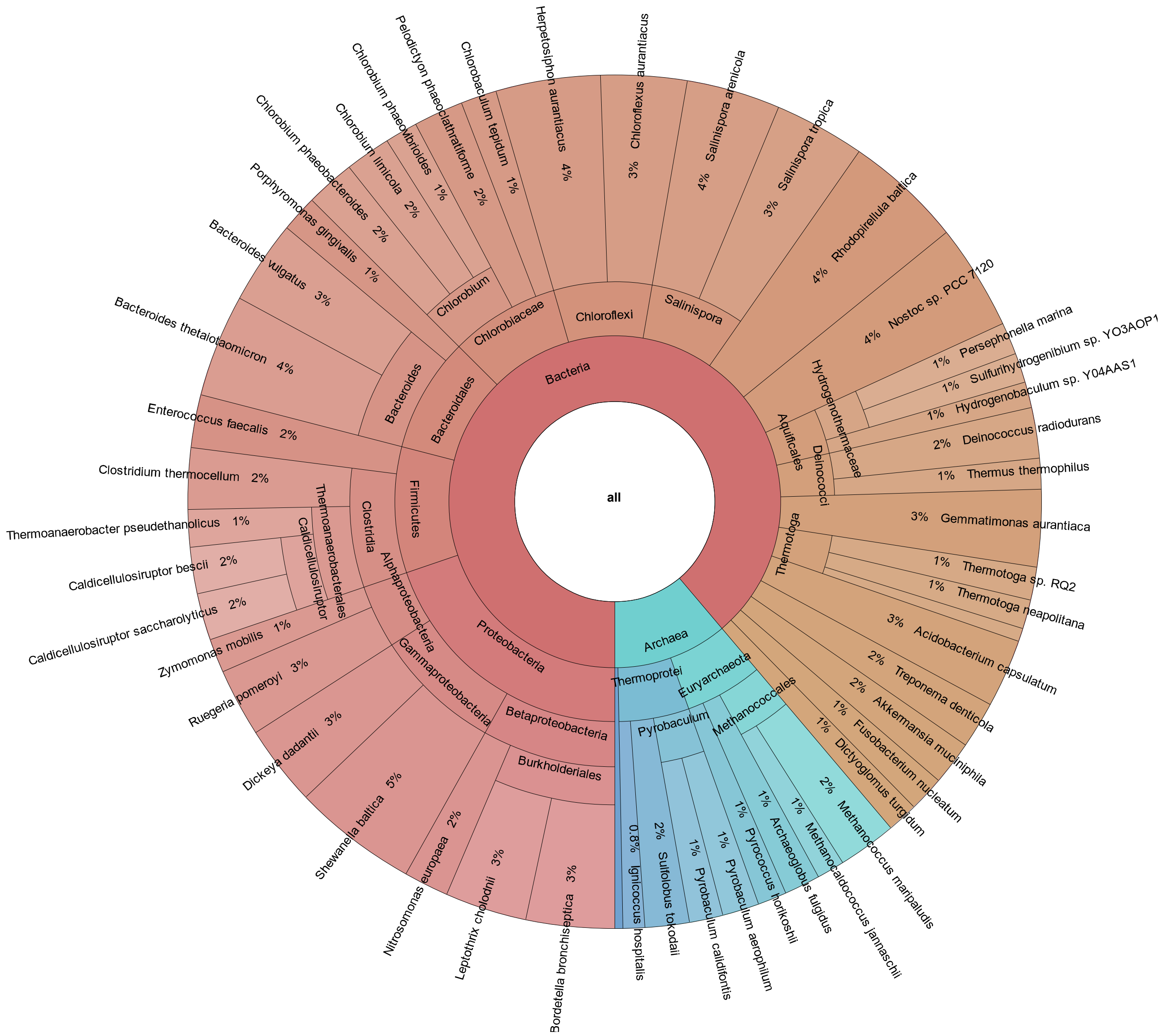

Taxonomic composition of the simulated metagenome sample simArt49e using Krona (Ondov et *al*., 2011). An interactive version can be found in the supplementary files (simArt49e.krona.html). Abundance is measured in terms of accumulated contigs lengths. The reads for this dataset were simulated using equal coverage for every strain, so differences in the data proportions result from a variable genome size and assembly bias.

Supplementary Figure S11 - CARMA binning of simulated metagenome with 49 species (simArt49e) **(a) summary scenario**

rank	depth	true (bp)	false (bp)	unknown (bp)	macro precision α=0.99	stdev	pred. bins	macro recall	stdev	real bins	sum true (bp)	sum false (bp)	overall prec.	description
unassigned	0	97460.6	0.0	0	100.0	0.0	1	100.0	0.0	1	115012.6	500.1	99.6	root+superkingdom
superkingdom	1	8776.0	500.1	0	93.6	3.0	2	64.1	17.1	2				
phylum	2	5011.0	7085.1	0	69.7	22.7	20	36.9	15.2	20				
class	3	4568.4	9153.1	0	47.0	38.5	36	33.4	11.8	23	17937.9	28325.0	38.8	phylum+class+order
order	4	8358.4	12086.7	0	31.8	39.5	78	29.2	9.5	32				
family	5	10303.4	12858.1	0	16.6	33.6	176	24.7	7.1	36				
genus	6	17193.4	13745.6	0	6.7	24.1	553	19.3	5.1	41	59286.4	54892.0	51.9	family+genus+species
species	7	31789.6	28288.3	0	2.9	16.5	1672	11.8	2.6	49				
avg/sum	4.2	86000.3	83717.1	0	38.3	25.4	362.4	31.3	9.8	29.0			50.7	all but unassigned
avg/sum	2.2	183460.9	83717.1	0	46.0	22.2	317.3	39.9	8.5	25.5			68.7	all with unassigned

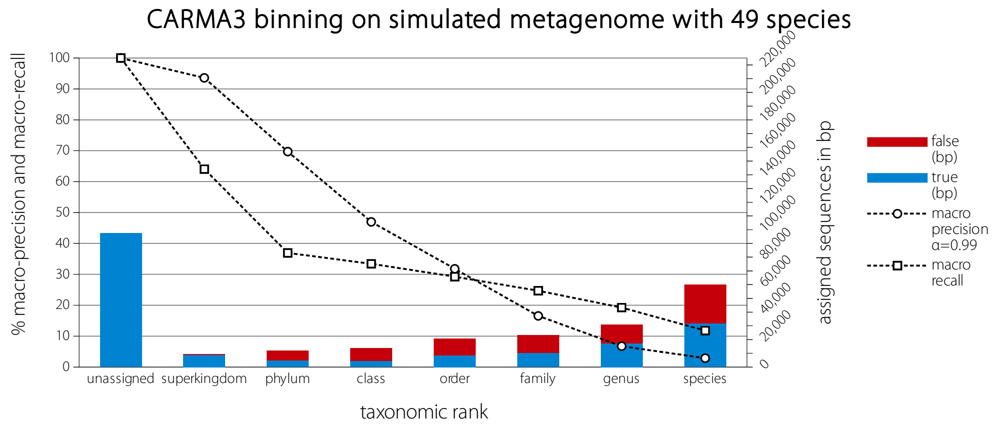

Supplementary Figure S11 - CARMA binning of simulated metagenome with 49 species (simArt49e) **(b) all reference scenario**

rank	depth	true (bp)	false (bp)	unknown (bp)	macro precision α=0.99	stdev	pred. bins	macro recall	stdev	real bins	sum true (bp)	sum false (bp)	overall prec.	description
unassigned	0	1071	0	0	100.0	0.0	1	100.0	0.0	1	1403	0	100.0	root+superkingdom
superkingdom	1	166	0	0	100.0	0.0	2	99.9	0.1	2				
phylum	2	130	1	0	100.0	0.0	19	99.9	0.2	20				
class	3	108	0	0	100.0	0.0	23	99.9	0.2	23	852	1	99.9	phylum+class+order
order	4	614	0	0	100.0	0.0	31	99.9	0.2	32				
family	5	1000	0	0	100.0	0.0	35	99.9	0.2	36				
genus	6	39774	30	0	100.0	0.0	40	99.8	0.2	41	263301	1787	99.3	family+genus+species
species	7	222527	1757	0	99.5	2.7	46	82.6	18.3	49				
avg/sum	5.9	264319	1788	0	99.9	0.4	28.0	97.4	2.8	29.0			99.3	all but unassigned
avg/sum	5.8	265390	1788	0	99.9	0.3	24.6	97.7	2.4	25.5			99.3	all with unassigned

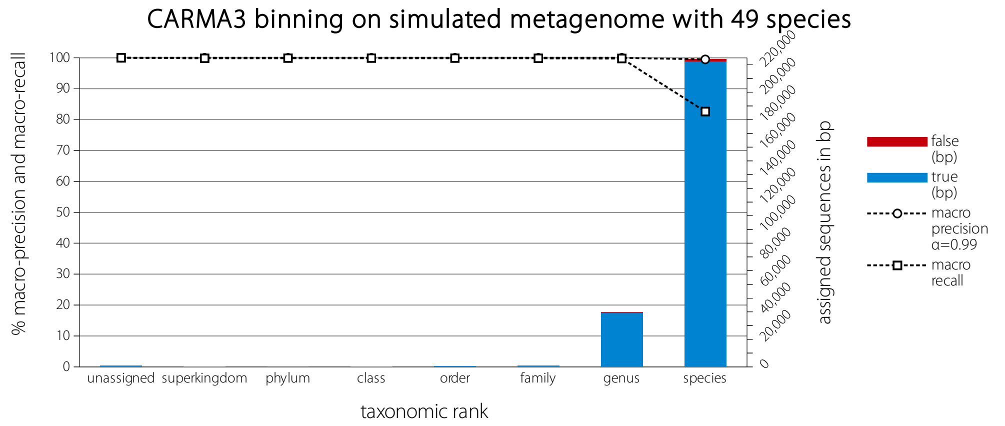

Supplementary Figure S11 - CARMA binning of simulated metagenome with 49 species (simArt49e) **(c) new species scenario**

rank	depth	true (bp)	false (bp)	unknown (bp)	macro precision α=0.99	stdev	pred. bins	macro recall	stdev	real bins	sum true (bp)	sum false (bp)	overall prec.	description
unassigned	0	48039	0	0	100.0	0.0	1	100.0	0.0	1	55507	174	99.7	root+superkingdom
superkingdom	1	3734	174	0	98.9	0.2	2	82.3	8.3	2				
phylum	2	5195	1718	0	93.8	5.8	17	65.6	31.8	20				
class	3	6657	2267	0	81.4	31.7	24	63.8	28.3	23	29216	7007	80.7	phylum+class+order
order	4	17364	3022	0	56.4	46.1	48	59.4	32.1	32				
family	5	43855	4055	0	32.0	44.7	96	53.0	33.4	36				
genus	6	80580	10693	0	12.5	32.0	216	35.0	35.5	41	124435	54573	69.5	family+genus+species
species	7	0	39825	0	0.0	0.0	1153	0.0	0.0	49				
avg/sum	5.1	157385	61754	0	53.6	22.9	222.3	51.3	24.2	29.0			71.8	all but unassigned
avg/sum	4.0	205424	61754	0	59.4	20.0	194.6	57.4	21.2	25.5			76.9	all with unassigned

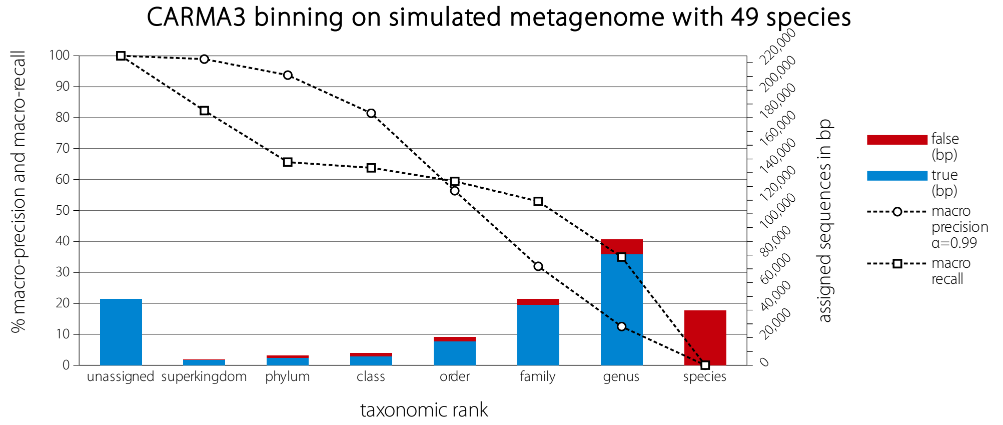

Supplementary Figure S11 - CARMA binning of simulated metagenome with 49 species (simArt49e) **(d) new genus scenario**

rank	depth	true (bp)	false (bp)	unknown (bp)	macro precision α=0.99	stdev	pred. bins	macro recall	stdev	real bins	sum true (bp)	sum false (bp)	overall prec.	description
unassigned	0	101939	0	0	100.0	0.0	1	100.0	0.0	1	116715	386	99.7	root+superkingdom
superkingdom	1	7388	386	0	96.1	1.9	2	67.2	14.9	2				
phylum	2	7629	4042	0	78.4	17.6	17	40.7	29.4	20				
class	3	8751	5633	0	53.1	39.5	31	38.3	26.6	23	36454	18595	66.2	phylum+class+order
order	4	20074	8920	0	33.8	39.9	65	32.7	27.5	32				
family	5	27269	13450	0	12.5	29.1	156	20.1	23.1	36				
genus	6	0	33904	0	0.0	0.0	535	0.0	0.0	41	27269	75147	26.6	family+genus+species
species	7	0	27793	0	0.0	0.0	1788	0.0	0.0	49				
avg/sum	4.3	71111	94128	0	39.1	18.3	370.6	28.4	17.4	29.0			43.0	all but unassigned
avg/sum	2.5	173050	94128	0	46.7	16.0	324.4	37.4	15.2	25.5			64.8	all with unassigned

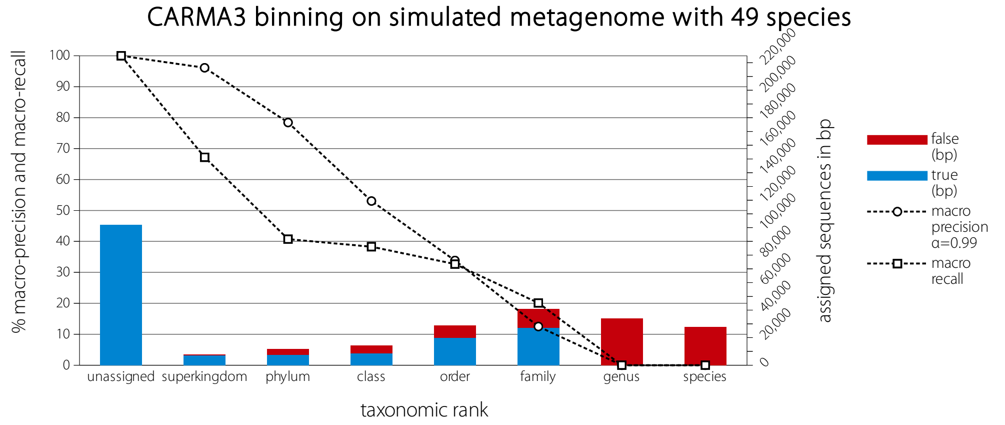

Supplementary Figure S11 - CARMA binning of simulated metagenome with 49 species (simArt49e) **(e) new family scenario**

rank	depth	true (bp)	false (bp)	unknown (bp)	macro precision α=0.99	stdev	pred. bins	macro recall	stdev	real bins	sum true (bp)	sum false (bp)	overall prec.	description
unassigned	0	114225	0	0	100.0	0.0	1	100.0	0.0	1	136949	536	99.6	root+superkingdom
superkingdom	1	11362	536	0	92.1	3.8	2	57.6	21.4	2				
phylum	2	9527	6860	0	48.3	32.1	18	26.3	27.3	20	39216	29057	57.4	phylum+class+order
class	3	9232	8904	0	23.8	32.6	36	21.8	25.3	23				
order	4	20457	13293	0	9.7	22.8	81	12.7	19.9	32				
family	5	0	24317	0	0.0	0.0	196	0.0	0.0	36	0	72782	0.0	family+genus+species
genus	6	0	18709	0	0.0	0.0	625	0.0	0.0	41				
species	7	0	29756	0	0.0	0.0	1816	0.0	0.0	49				
avg/sum	3.8	50578	102375	0	24.8	13.0	396.3	16.9	13.4	29.0			33.1	all but unassigned
avg/sum	2.0	164803	102375	0	34.2	11.4	346.9	27.3	11.7	25.5			61.7	all with unassigned

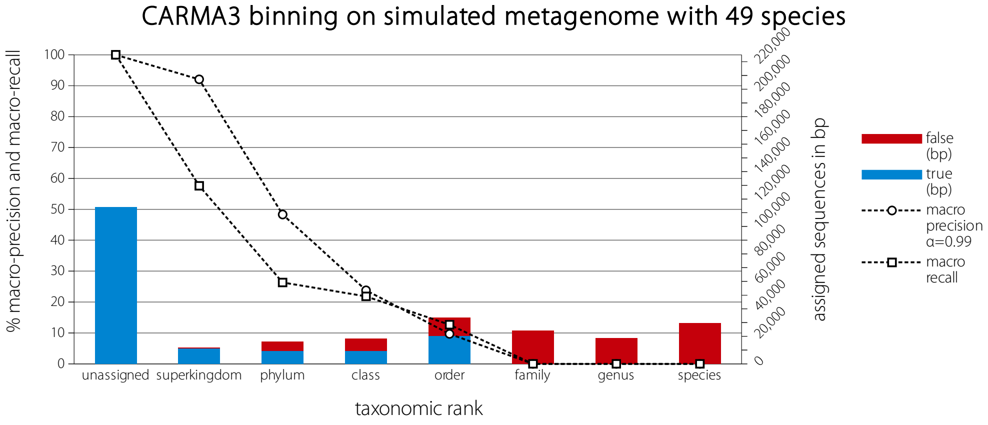

Supplementary Figure S11 - CARMA binning of simulated metagenome with 49 species (simArt49e) **(f) new order scenario**

rank	depth	true (bp)	false (bp)	unknown (bp)	macro precision α=0.99	stdev	pred. bins	macro recall	stdev	real bins	sum true (bp)	sum false (bp)	overall prec.	description
unassigned	0	130123	0	0	100.0	0.0	1	100.0	0.0	1	154807	706	99.5	root+superkingdom
superkingdom	1	12342	706	0	87.7	7.2	2	52.3	23.4	2				
phylum	2	8019	10411	0	27.9	30.5	21	17.0	23.2	20	15250	45492	25.1	phylum+class+order
class	3	7231	14435	0	10.0	21.5	39	10.1	16.8	23				
order	4	0	20646	0	0.0	0.0	90	0.0	0.0	32				
family	5	0	18233	0	0.0	0.0	203	0.0	0.0	36	0	63265	0.0	family+genus+species
genus	6	0	12779	0	0.0	0.0	652	0.0	0.0	41				
species	7	0	32253	0	0.0	0.0	1810	0.0	0.0	49				
avg/sum	3.5	27592	109463	0	17.9	8.5	402.4	11.4	9.0	29.0			20.1	all but unassigned
avg/sum	1.6	157715	109463	0	28.2	7.4	352.3	22.4	7.9	25.5			59.0	all with unassigned

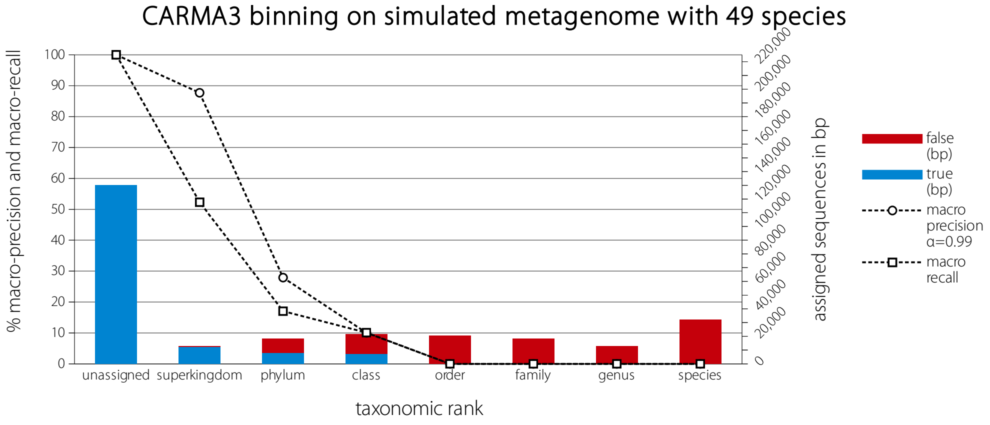

Supplementary Figure S11 - CARMA binning of simulated metagenome with 49 species (simArt49e) **(g) new class scenario**

rank	depth	true (bp)	false (bp)	unknown (bp)	macro precision α=0.99	stdev	pred. bins	macro recall	stdev	real bins	sum true (bp)	sum false (bp)	overall prec.	description
unassigned	0	139175	0	0	100.0	0.0	1	100.0	0.0	1	165277	778	99.5	root+superkingdom
superkingdom	1	13051	778	0	84.3	9.8	2	48.2	24.2	2				
phylum	2	4577	12085	0	12.9	20.1	22	8.9	15.4	20	4577	50298	8.3	phylum+class+order
class	3	0	18605	0	0.0	0.0	41	0.0	0.0	23				
order	4	0	19608	0	0.0	0.0	91	0.0	0.0	32				
family	5	0	15771	0	0.0	0.0	206	0.0	0.0	36	0	59299	0.0	family+genus+species
genus	6	0	10539	0	0.0	0.0	657	0.0	0.0	41				
species	7	0	32989	0	0.0	0.0	1814	0.0	0.0	49				
avg/sum	3.4	17628	110375	0	13.9	4.3	404.7	8.2	5.7	29.0			13.8	all but unassigned
avg/sum	1.4	156803	110375	0	24.6	3.7	354.3	19.6	5.0	25.5			58.7	all with unassigned

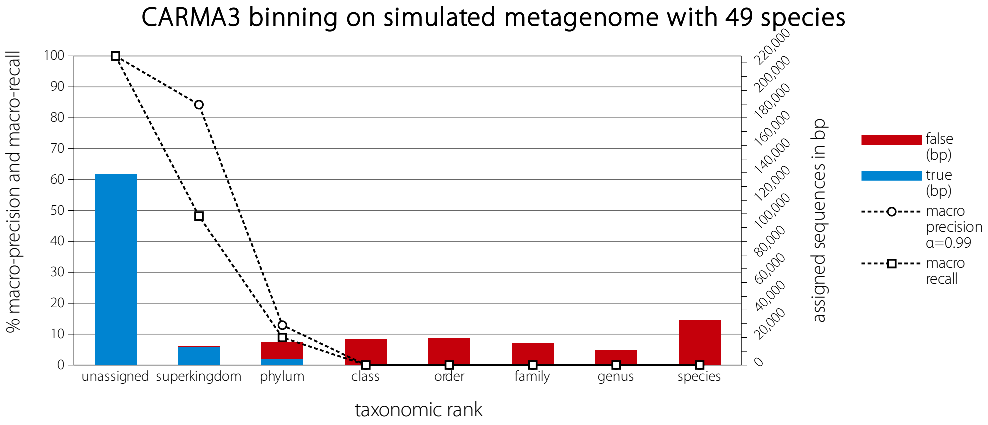

Supplementary Figure S11 - CARMA binning of simulated metagenome with 49 species (simArt49e) **(h) new phylum scenario**

rank	depth	true (bp)	false (bp)	unknown (bp)	macro precision α=0.99	stdev	pred. bins	macro recall	stdev	real bins	sum true (bp)	sum false (bp)	overall prec.	description
unassigned	0	147652	0	0	100.0	0.0	1	100.0	0.0	1	174430	921	99.5	root+superkingdom
superkingdom	1	13389	921	0	75.1	17.3	2	41.1	27.2	2				
phylum	2	0	14479	0	0.0	0.0	24	0.0	0.0	20	0	47825	0.0	phylum+class+order
class	3	0	14228	0	0.0	0.0	42	0.0	0.0	23				
order	4	0	19118	0	0.0	0.0	93	0.0	0.0	32				
family	5	0	14181	0	0.0	0.0	214	0.0	0.0	36	0	57391	0.0	family+genus+species
genus	6	0	9565	0	0.0	0.0	664	0.0	0.0	41				
species	7	0	33645	0	0.0	0.0	1820	0.0	0.0	49				
avg/sum	3.4	13389	106137	0	10.7	2.5	408.4	5.9	3.9	29.0			11.2	all but unassigned
avg/sum	1.2	161041	106137	0	21.9	2.2	357.5	17.6	3.4	25.5			60.3	all with unassigned

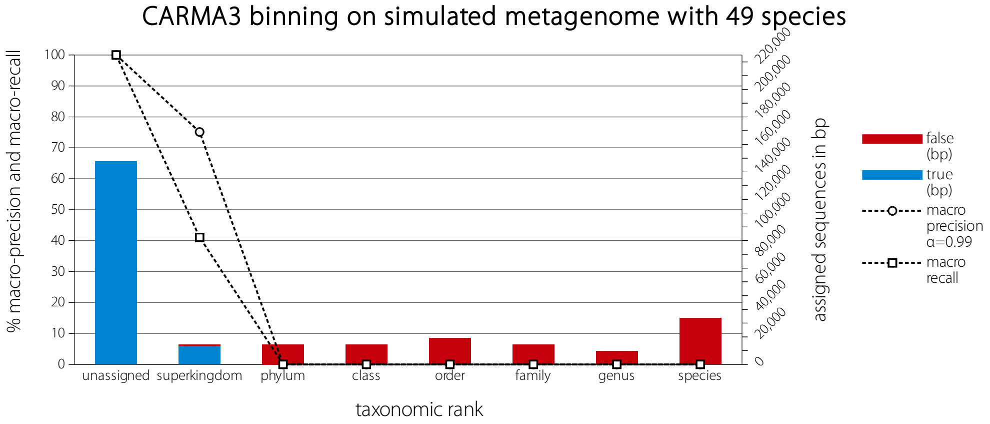

Supplementary Figure S12 - MEGAN binning of simulated metagenome with 49 species (simArt49e) **(a) summary scenario**

rank	depth	true (bp)	false (bp)	unknown (bp)	macro precision α=0.99	stdev	pred. bins	macro recall	stdev	real bins	sum true (bp)	sum false (bp)	overall prec.	description
unassigned	0	62255.4	0.0	0	100.0	0.0	1	100.0	0.0	1	232794.3	8388.1	96.5	root+superkingdom
superkingdom	1	85269.4	8388.1	0	97.8	0.1	2	64.8	21.3	2				
phylum	2	5415.0	3937.6	0	89.4	8.3	19	35.9	14.3	20	14756.4	7006.7	67.8	phylum+class+order
class	3	3302.9	1523.4	0	61.7	41.3	33	34.3	9.9	23				
order	4	6038.6	1545.7	0	43.3	45.1	66	32.3	9.3	32				
family	5	6638.4	2415.9	0	22.4	38.7	139	28.0	8.0	36	53023.9	36478.0	59.2	family+genus+species
genus	6	18552.9	6525.4	0	9.3	27.9	400	21.2	5.9	41				
species	7	27832.6	27536.7	0	5.4	21.9	824	11.8	4.5	49				
avg/sum	2.4	153049.7	51872.9	0	47.0	26.2	211.9	32.6	10.4	29.0			74.7	all but unassigned
avg/sum	1.7	215305.1	51872.9	0	53.7	22.9	185.5	41.0	9.1	25.5			80.6	all with unassigned

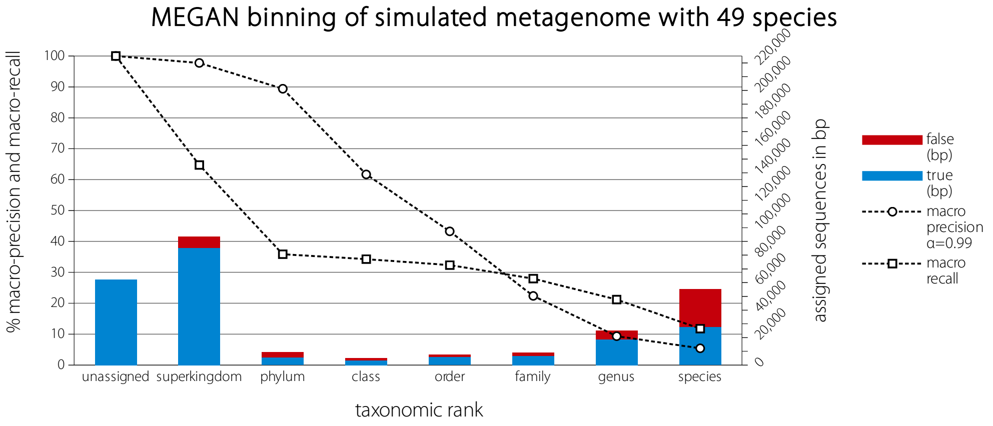

Supplementary Figure S12 - MEGAN binning of simulated metagenome with 49 species (simArt49e) **(b) all reference scenario**

rank	depth	true (bp)	false (bp)	unknown (bp)	macro precision α=0.99	stdev	pred. bins	macro recall	stdev	real bins	sum true (bp)	sum false (bp)	overall prec.	description
unassigned	0	595	0	0	100.0	0.0	1	100.0	0.0	1	2301	0	100.0	root+superkingdom
superkingdom	1	853	0	0	100.0	0.0	2	99.9	0.1	2				
phylum	2	515	1	0	100.0	0.0	19	99.9	0.1	20	2948	1	100.0	phylum+class+order
class	3	399	0	0	100.0	0.0	23	99.9	0.1	23				
order	4	2034	0	0	100.0	0.0	31	99.9	0.1	32				
family	5	5388	0	0	100.0	0.0	35	99.8	0.3	36	262771	10	100.0	family+genus+species
genus	6	62555	0	0	100.0	0.0	40	99.3	1.6	41				
species	7	194828	10	0	100.0	0.0	44	82.5	31.3	49				
avg/sum	5.8	266572	11	0	100.0	0.0	27.7	97.3	4.8	29.0			100.0	all but unassigned
avg/sum	5.7	267167	11	0	100.0	0.0	24.4	97.7	4.2	25.5			100.0	all with unassigned

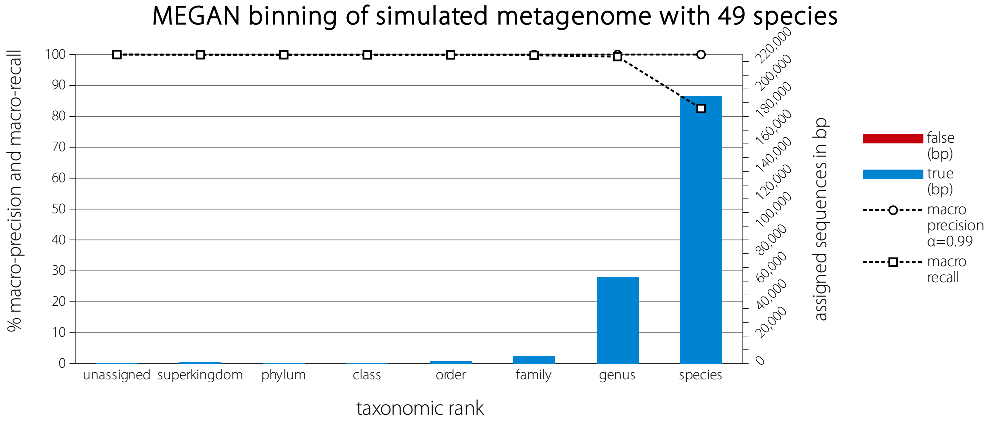

Supplementary Figure S12 - MEGAN binning of simulated metagenome with 49 species (simArt49e) **(c) new species scenario**

rank	depth	true (bp)	false (bp)	unknown (bp)	macro precision α=0.99	stdev	pred. bins	macro recall	stdev	real bins	sum true (bp)	sum false (bp)	overall prec.	description
unassigned	0	22828	0	0	100.0	0.0	1	100.0	0.0	1	91304	3128	96.7	root+superkingdom
superkingdom	1	34238	3128	0	99.6	0.1	2	87.1	9.1	2				
phylum	2	3671	867	0	98.4	1.9	17	67.3	33.8	20	15968	1537	91.2	phylum+class+order
class	3	3130	282	0	98.4	1.8	21	70.8	29.0	23				
order	4	9167	388	0	83.5	34.4	35	70.5	31.9	32				
family	5	20053	979	0	58.9	47.3	55	65.3	34.3	36	87368	102111	46.1	family+genus+species
genus	6	67315	3069	0	22.9	41.1	121	49.0	40.8	41				
species	7	0	98063	0	0.0	0.0	218	0.0	0.0	49				
avg/sum	4.3	137574	106776	0	66.0	18.1	67.0	58.6	25.6	29.0			56.3	all but unassigned
avg/sum	3.7	160402	106776	0	70.2	15.8	58.8	63.8	22.4	25.5			60.0	all with unassigned

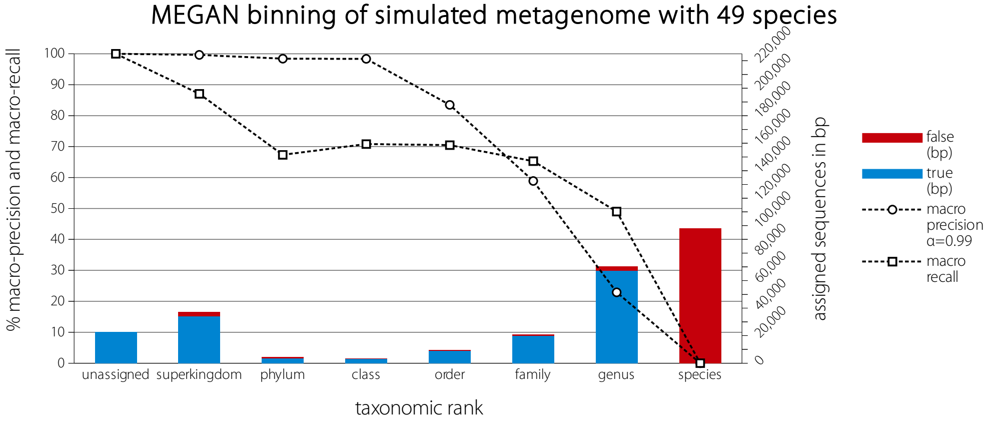

Supplementary Figure S12 - MEGAN binning of simulated metagenome with 49 species (simArt49e) **(d) new genus scenario**

rank	depth	true (bp)	false (bp)	unknown (bp)	macro precision α=0.99	stdev	pred. bins	macro recall	stdev	real bins	sum true (bp)	sum false (bp)	overall prec.	description
unassigned	0	58109	0	0	100.0	0.0	1	100.0	0.0	1	238003	6636	97.3	root+superkingdom
superkingdom	1	89947	6636	0	98.8	0.0	2	74.5	15.4	2				
phylum	2	8288	1861	0	93.1	9.2	15	40.0	29.9	20	29489	3515	89.3	phylum+class+order
class	3	6666	657	0	76.6	34.7	25	41.8	26.2	23				
order	4	14535	997	0	52.0	45.1	50	41.1	29.8	32				
family	5	21028	3013	0	22.3	38.5	105	30.6	30.6	36	21028	58454	26.5	family+genus+species
genus	6	0	20343	0	0.0	0.0	274	0.0	0.0	41				
species	7	0	35098	0	0.0	0.0	430	0.0	0.0	49				
avg/sum	2.5	140464	68605	0	49.0	18.2	128.7	32.6	18.8	29.0			67.2	all but unassigned
avg/sum	1.9	198573	68605	0	55.3	15.9	112.8	41.0	16.5	25.5			74.3	all with unassigned

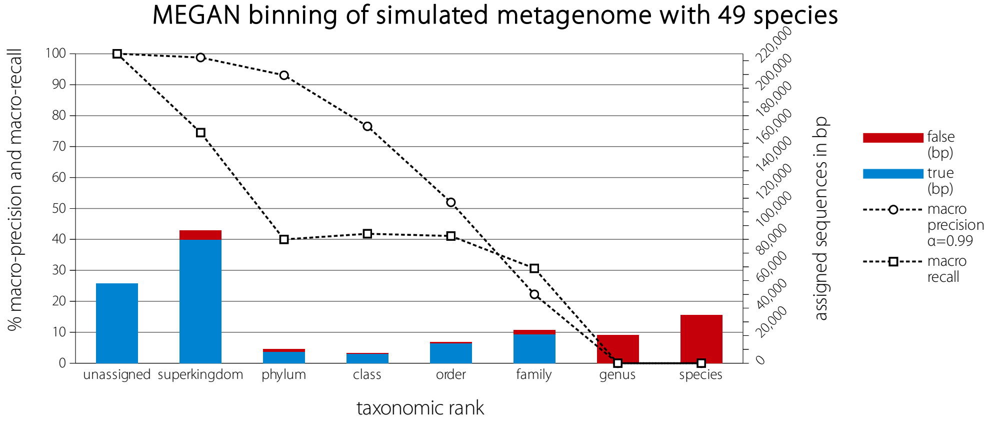

Supplementary Figure S12 - MEGAN binning of simulated metagenome with 49 species (simArt49e) **(e) new family scenario**

rank	depth	true (bp)	false (bp)	unknown (bp)	macro precision α=0.99	stdev	pred. bins	macro recall	stdev	real bins	sum true (bp)	sum false (bp)	overall prec.	description
unassigned	0	73809	0	0	100.0	0.0	1	100.0	0.0	1	296855	8005	97.4	root+superkingdom
superkingdom	1	111523	8005	0	97.2	0.4	2	56.5	28.9	2				
phylum	2	10028	2966	0	78.2	27.3	14	23.5	25.8	20				
class	3	7514	1043	0	37.5	42.3	31	20.4	21.9	23	34076	5490	86.1	phylum+class+order
order	4	16534	1481	0	17.5	32.9	69	14.9	21.1	32				
family	5	0	5906	0	0.0	0.0	161	0.0	0.0	36				
genus	6	0	8487	0	0.0	0.0	366	0.0	0.0	41	0	34275	0.0	family+genus+species
species	7	0	19882	0	0.0	0.0	580	0.0	0.0	49				
avg/sum	1.9	145599	47770	0	32.9	14.7	174.7	16.5	14.0	29.0			75.3	all but unassigned
avg/sum	1.3	219408	47770	0	41.3	12.9	153.0	26.9	12.2	25.5			82.1	all with unassigned

MEGAN binning of simulated metagenome with 49 species

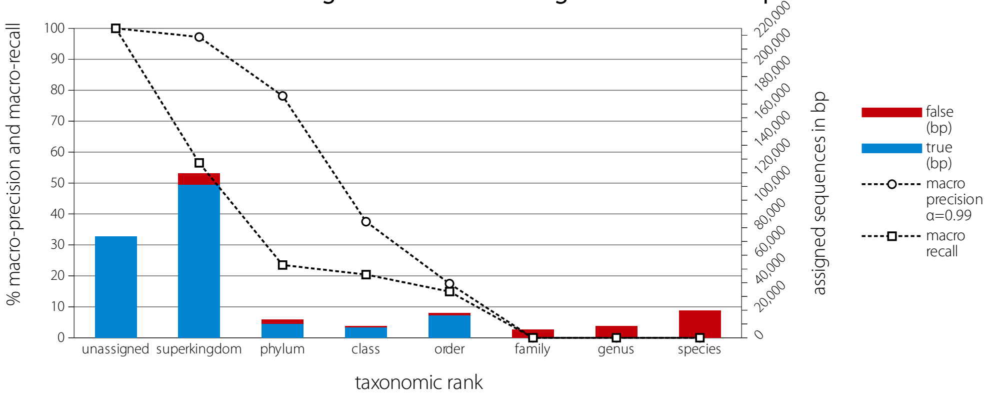

Supplementary Figure S12 - MEGAN binning of simulated metagenome with 49 species (simArt49e) **(f) new order scenario**

rank	depth	true (bp)	false (bp)	unknown (bp)	macro precision α=0.99	stdev	pred. bins	macro recall	stdev	real bins	sum true (bp)	sum false (bp)	overall prec.	description
unassigned	0	89682	0	0	100.0	0.0	1	100.0	0.0	1	326876	11005	96.7	root+superkingdom
superkingdom	1	118597	11005	0	95.5	1.3	2	49.8	30.9	2				
phylum	2	10011	5581	0	47.4	37.8	17	13.8	21.7	20				
class	3	5411	1881	0	14.4	28.3	39	7.0	11.7	23	15422	9908	60.9	phylum+class+order
order	4	0	2446	0	0.0	0.0	84	0.0	0.0	32				
family	5	0	3416	0	0.0	0.0	167	0.0	0.0	36				
genus	6	0	5382	0	0.0	0.0	401	0.0	0.0	41	0	22564	0.0	family+genus+species
species	7	0	13766	0	0.0	0.0	565	0.0	0.0	49				
avg/sum	1.5	134019	43477	0	22.5	9.6	182.1	10.1	9.2	29.0			75.5	all but unassigned
avg/sum	1.0	223701	43477	0	32.2	8.4	159.5	21.3	8.0	25.5			83.7	all with unassigned

MEGAN binning of simulated metagenome with 49 species

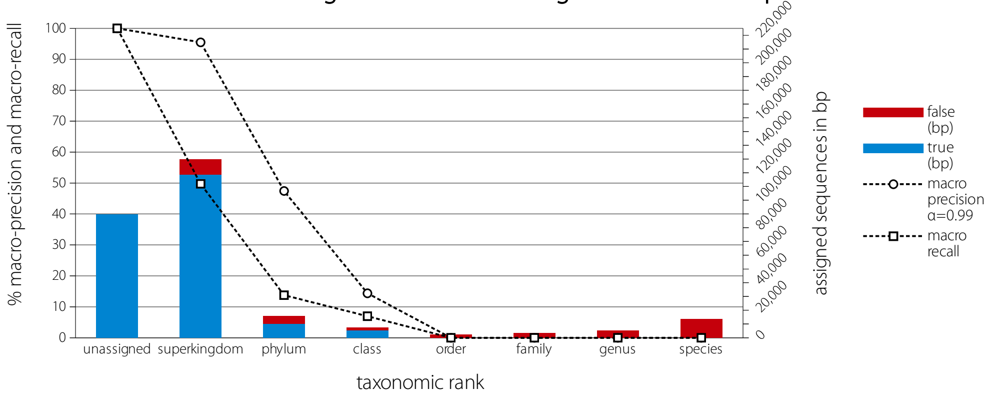

Supplementary Figure S12 - MEGAN binning of simulated metagenome with 49 species (simArt49e) **(g) new class scenario**

rank	depth	true (bp)	false (bp)	unknown (bp)	macro precision α=0.99	stdev	pred. bins	macro recall	stdev	real bins	sum true (bp)	sum false (bp)	overall prec.	description
unassigned	0	94817	0	0	100.0	0.0	1	100.0	0.0	1	340147	11467	96.7	root+superkingdom
superkingdom	1	122665	11467	0	93.2	2.8	2	45.9	31.8	2				
phylum	2	5392	7208	0	25.1	30.7	19	6.4	11.6	20				
class	3	0	4356	0	0.0	0.0	43	0.0	0.0	23	5392	13709	28.2	phylum+class+order
order	4	0	2145	0	0.0	0.0	88	0.0	0.0	32				
family	5	0	2203	0	0.0	0.0	172	0.0	0.0	36				
genus	6	0	4437	0	0.0	0.0	446	0.0	0.0	41	0	19128	0.0	family+genus+species
species	7	0	12488	0	0.0	0.0	657	0.0	0.0	49				
avg/sum	1.4	128057	44304	0	16.9	4.8	203.9	7.5	6.2	29.0			74.3	all but unassigned
avg/sum	0.9	222874	44304	0	27.3	4.2	178.5	19.0	5.4	25.5			83.4	all with unassigned

MEGAN binning of simulated metagenome with 49 species

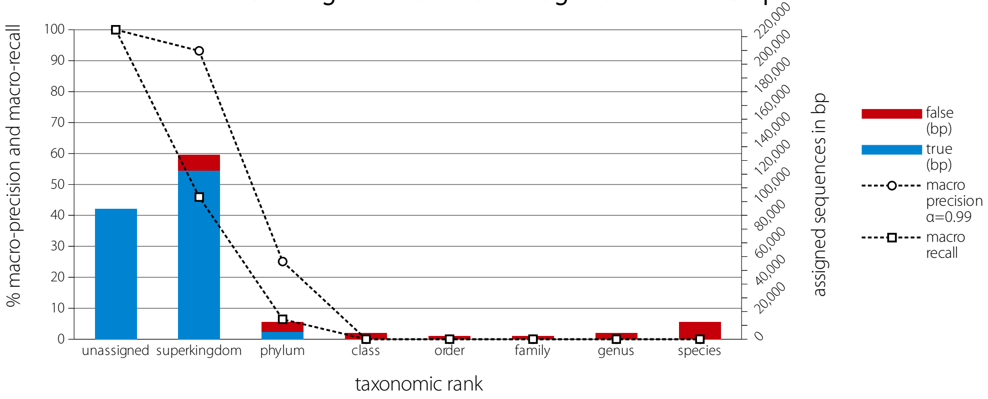

Supplementary Figure S12 - MEGAN binning of simulated metagenome with 49 species (simArt49e) **(h) new phylum scenario**

rank	depth	true (bp)	false (bp)	unknown (bp)	macro precision α=0.99	stdev	pred. bins	macro recall	stdev	real bins	sum true (bp)	sum false (bp)	overall prec.	description
unassigned	0	95948	0	0	100.0	0.0	1	100.0	0.0	1	334074	18476	94.8	root+superkingdom
superkingdom	1	119063	18476	0	84.6	8.8	2	39.5	33.1	2				
phylum	2	0	9079	0	0.0	0.0	25	0.0	0.0	20				
class	3	0	2445	0	0.0	0.0	45	0.0	0.0	23	0	14887	0.0	phylum+class+order
order	4	0	3363	0	0.0	0.0	96	0.0	0.0	32				
family	5	0	1394	0	0.0	0.0	197	0.0	0.0	36				
genus	6	0	3960	0	0.0	0.0	494	0.0	0.0	41	0	18804	0.0	family+genus+species
species	7	0	13450	0	0.0	0.0	814	0.0	0.0	49				
avg/sum	1.3	119063	52167	0	12.1	1.3	239.0	5.6	4.7	29.0			69.5	all but unassigned
avg/sum	0.8	215011	52167	0	23.1	1.1	209.3	17.4	4.1	25.5			80.5	all with unassigned

MEGAN binning of simulated metagenome with 49 species

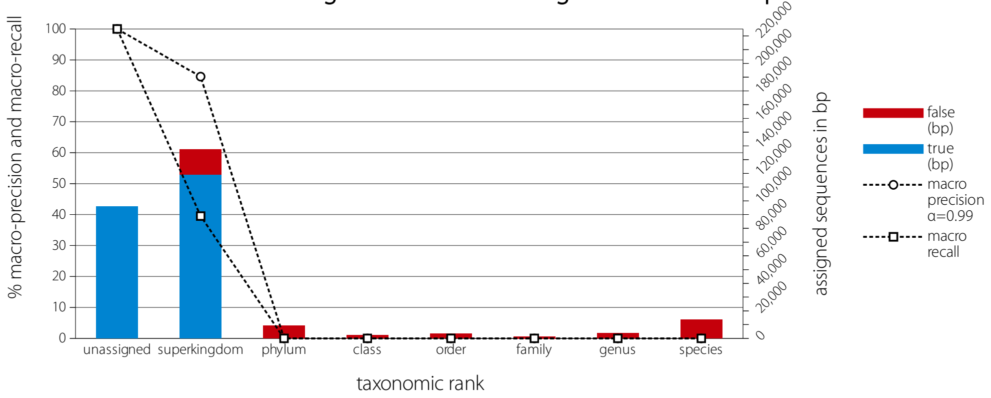

Supplementary Figure S13 - Taxator-tk binning of simulated metagenome with 49 species (simArt49e) **(a) summary scenario**

rank	depth	true (bp)	false (bp)	unknown (bp)	macro precision α=0.99	stdev	pred. bins	macro recall	stdev	real bins	sum true (bp)	sum false (bp)	overall prec.	description
unassigned	0	75644.4	0.0	0	100.0	0.0	1	100.0	0.0	1	288633.9	10293.3	96.6	root+superkingdom
superkingdom	1	106494.7	10293.3	0	96.9	2.5	2	56.8	33.5	2				
phylum	2	7691.6	9493.1	0	94.9	9.2	16	18.2	13.5	20				
class	3	3656.6	2344.0	0	91.2	21.5	21	18.3	11.6	23	17180.7	13851.4	55.4	phylum+class+order
order	4	5832.6	2014.3	0	85.9	31.8	34	16.2	9.5	32				
family	5	7550.6	1079.7	0	76.4	39.8	44	13.8	8.2	36				
genus	6	20271.9	1397.1	0	65.9	46.4	58	9.4	7.7	41	39774.7	3938.7	91.0	family+genus+species
species	7	11952.3	1461.9	0	61.1	47.2	65	2.5	4.4	49				
avg/sum	2.1	163450.1	28083.4	0	81.8	28.3	34.3	19.3	12.6	29.0			85.3	all but unassigned
avg/sum	1.5	239094.6	28083.4	0	84.0	24.8	30.1	29.4	11.0	25.5			89.5	all with unassigned

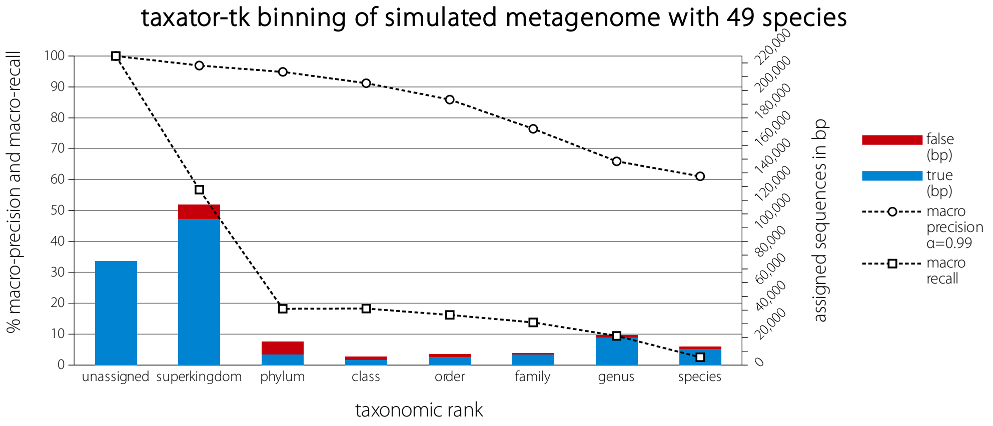

Supplementary Figure S13 - Taxator-tk binning of simulated metagenome with 49 species (simArt49e) **(b) all reference scenario**

rank	depth	true (bp)	false (bp)	unknown (bp)	macro precision α=0.99	stdev	pred. bins	macro recall	stdev	real bins	sum true (bp)	sum false (bp)	overall prec.	description
unassigned	0	34453	0	0	100.0	0.0	1	100.0	0.0	1	105775	0	100.0	root+superkingdom
superkingdom	1	35661	0	0	100.0	0.0	2	68.1	8.4	2				
phylum	2	2897	0	0	100.0	0.0	17	46.5	28.4	20				
class	3	1947	0	0	100.0	0.0	21	52.5	29.1	23	13098	0	100.0	phylum+class+order
order	4	8254	0	0	100.0	0.0	29	51.7	30.6	32				
family	5	19632	0	0	100.0	0.0	32	52.1	31.5	36				
genus	6	80667	0	0	100.0	0.0	34	43.4	34.0	41	183965	1	100.0	family+genus+species
species	7	83666	1	0	100.0	0.0	34	17.7	30.5	49				
avg/sum	4.4	232724	1	0	100.0	0.0	24.1	47.4	27.5	29.0			100.0	all but unassigned
avg/sum	3.6	267177	1	0	100.0	0.0	21.3	54.0	24.1	25.5			100.0	all with unassigned

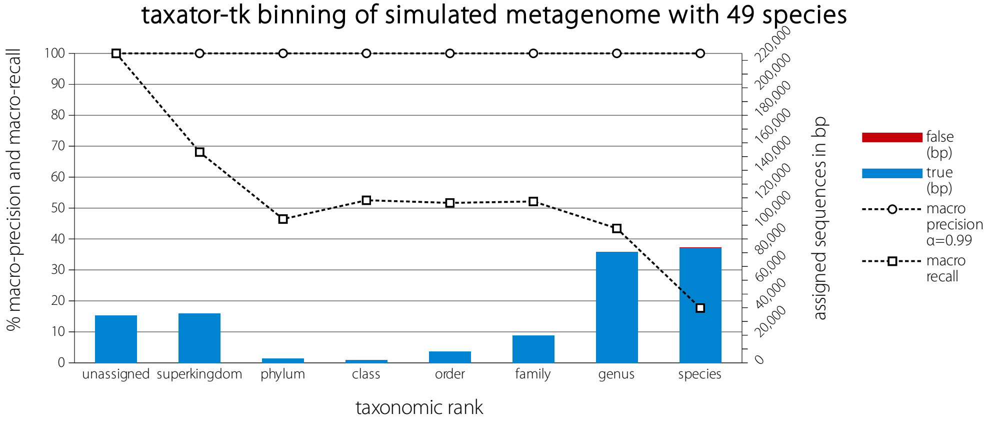

Supplementary Figure S13 - Taxator-tk binning of simulated metagenome with 49 species (simArt49e) **(c) new species scenario**

rank	depth	true (bp)	false (bp)	unknown (bp)	macro precision α=0.99	stdev	pred. bins	macro recall	stdev	real bins	sum true (bp)	sum false (bp)	overall prec.	description
unassigned	0	63523	0	0	100.0	0.0	1	100.0	0.0	1	214561	4247	98.1	root+superkingdom
superkingdom	1	75519	4247	0	99.1	0.9	2	74.7	16.4	2				
phylum	2	8526	1834	0	99.4	1.4	15	39.3	28.6	20				
class	3	6516	515	0	99.7	0.5	18	42.8	27.4	23	29377	2671	91.7	phylum+class+order
order	4	14335	322	0	99.7	0.5	25	39.9	26.9	32				
family	5	21470	246	0	99.7	0.8	28	34.9	28.6	36				
genus	6	61236	1365	0	88.7	30.3	26	22.6	28.3	41	82706	9135	90.1	family+genus+species
species	7	0	7524	0	0.0	0.0	48	0.0	0.0	49				
avg/sum	3.4	187602	16053	0	83.8	4.9	23.1	36.3	22.3	29.0			92.1	all but unassigned
avg/sum	2.6	251125	16053	0	85.8	4.3	20.4	44.3	19.5	25.5			94.0	all with unassigned

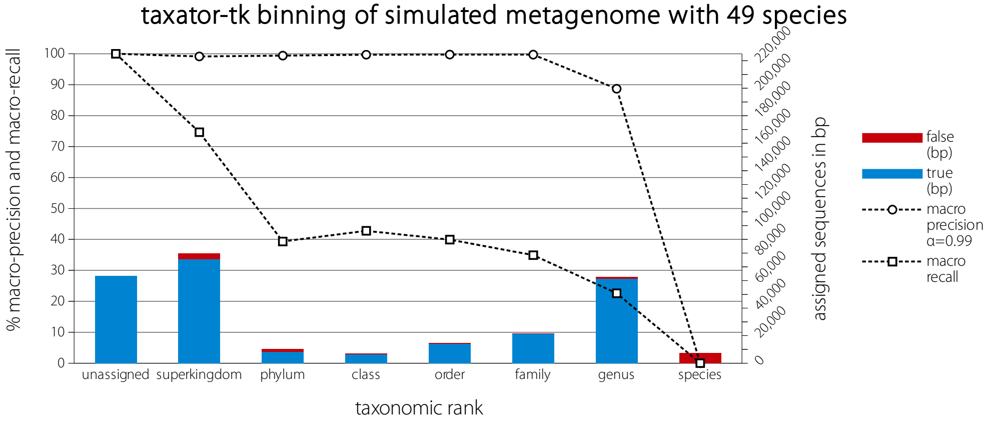

Supplementary Figure S13 - Taxator-tk binning of simulated metagenome with 49 species (simArt49e) **(d) new genus scenario**

rank	depth	true (bp)	false (bp)	unknown (bp)	macro precision α=0.99	stdev	pred. bins	macro recall	stdev	real bins	sum true (bp)	sum false (bp)	overall prec.	description
unassigned	0	82737	0	0	100.0	0.0	1	100.0	0.0	1	318017	8730	97.3	root+superkingdom
superkingdom	1	117640	8730	0	98.1	1.4	2	61.5	31.7	2				
phylum	2	12404	4567	0	98.2	3.3	13	20.1	21.3	20				
class	3	6555	1439	0	98.5	1.9	16	20.9	21.8	23	31213	7247	81.2	phylum+class+order
order	4	12254	1241	0	97.1	4.9	22	17.6	20.2	32				
family	5	11752	1508	0	56.2	46.1	33	9.5	16.7	36				
genus	6	0	4633	0	0.0	0.0	52	0.0	0.0	41	11752	7859	59.9	family+genus+species
species	7	0	1718	0	0.0	0.0	49	0.0	0.0	49				
avg/sum	1.8	160605	23836	0	64.0	8.2	26.7	18.5	16.0	29.0			87.1	all but unassigned
avg/sum	1.3	243342	23836	0	68.5	7.2	23.5	28.7	14.0	25.5			91.1	all with unassigned

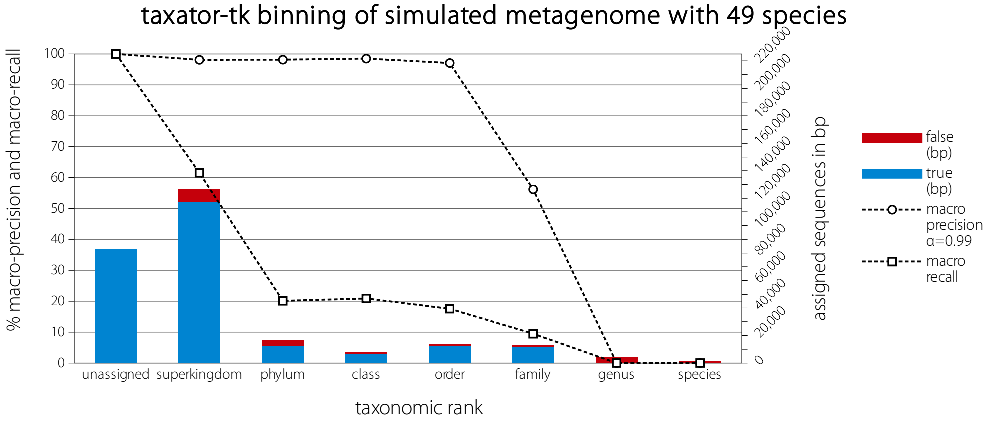

Supplementary Figure S13 - Taxator-tk binning of simulated metagenome with 49 species (simArt49e) **(e) new family scenario**

rank	depth	true (bp)	false (bp)	unknown (bp)	macro precision α=0.99	stdev	pred. bins	macro recall	stdev	real bins	sum true (bp)	sum false (bp)	overall prec.	description
unassigned	0	86887	0	0	100.0	0.0	1	100.0	0.0	1	345265	10849	97.0	root+superkingdom
superkingdom	1	129189	10849	0	95.9	2.0	2	50.8	42.7	2				
phylum	2	13258	6399	0	95.0	6.2	8	12.1	19.5	20	25853	9802	72.5	phylum+class+order
class	3	6610	1866	0	96.0	4.4	12	8.9	14.0	23				
order	4	5985	1537	0	38.6	46.6	27	4.3	8.3	32				
family	5	0	2768	0	0.0	0.0	48	0.0	0.0	36	0	4598	0.0	family+genus+species
genus	6	0	1474	0	0.0	0.0	85	0.0	0.0	41				
species	7	0	356	0	0.0	0.0	81	0.0	0.0	49				
avg/sum	1.4	155042	25249	0	46.5	8.5	37.6	10.9	12.1	29.0			86.0	all but unassigned
avg/sum	1.0	241929	25249	0	53.2	7.4	33.0	22.0	10.6	25.5			90.5	all with unassigned

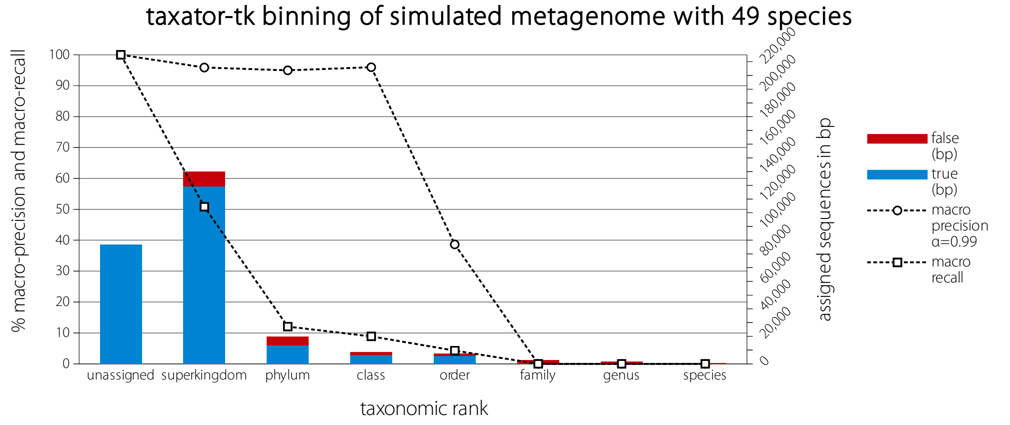

Supplementary Figure S13 - Taxator-tk binning of simulated metagenome with 49 species (simArt49e) **(f) new order scenario**

rank	depth	true (bp)	false (bp)	unknown (bp)	macro precision α=0.99	stdev	pred. bins	macro recall	stdev	real bins	sum true (bp)	sum false (bp)	overall prec.	description
unassigned	0	83674	0	0	100.0	0.0	1	100.0	0.0	1	345694	14172	96.1	root+superkingdom
superkingdom	1	131010	14172	0	92.7	0.0	1	48.7	45.1	2				
phylum	2	11454	14889	0	73.6	25.5	6	7.2	16.0	20	15422	20404	43.0	phylum+class+order
class	3	3968	3071	0	36.7	40.1	16	2.9	7.2	23				
order	4	0	2444	0	0.0	0.0	38	0.0	0.0	32				
family	5	0	1364	0	0.0	0.0	70	0.0	0.0	36	0	2496	0.0	family+genus+species
genus	6	0	901	0	0.0	0.0	103	0.0	0.0	41				
species	7	0	231	0	0.0	0.0	83	0.0	0.0	49				
avg/sum	1.3	146432	37072	0	29.0	9.4	45.3	8.4	9.7	29.0			79.8	all but unassigned
avg/sum	0.9	230106	37072	0	37.9	8.2	39.8	19.8	8.5	25.5			86.1	all with unassigned

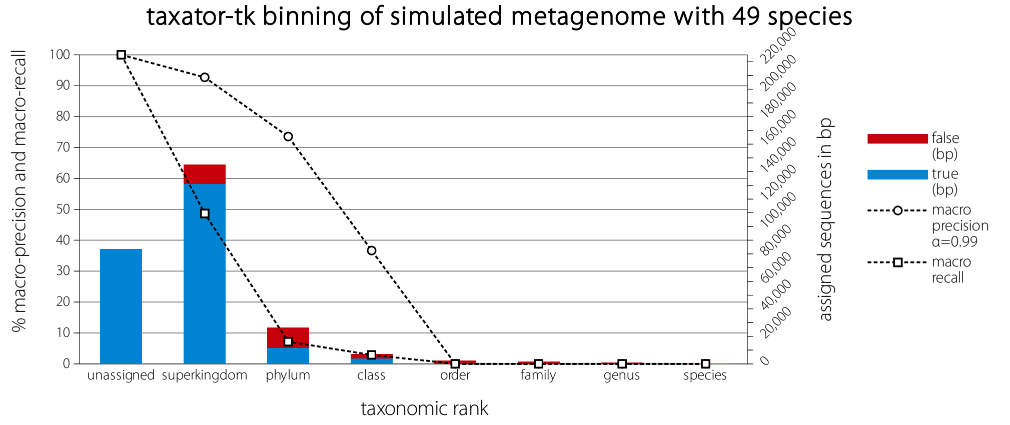

Supplementary Figure S13 - Taxator-tk binning of simulated metagenome with 49 species (simArt49e) **(g) new class scenario**

rank	depth	true (bp)	false (bp)	unknown (bp)	macro precision α=0.99	stdev	pred. bins	macro recall	stdev	real bins	sum true (bp)	sum false (bp)	overall prec.	description
unassigned	0	88280	0	0	100.0	0.0	1	100.0	0.0	1	352286	14601	96.0	root+superkingdom
superkingdom	1	132003	14601	0	91.8	0.0	1	47.9	44.8	2				
phylum	2	5302	17992	0	51.5	32.3	5	2.3	5.5	20	5302	25163	17.4	phylum+class+order
class	3	0	4839	0	0.0	0.0	17	0.0	0.0	23				
order	4	0	2332	0	0.0	0.0	41	0.0	0.0	32				
family	5	0	961	0	0.0	0.0	73	0.0	0.0	36	0	1829	0.0	family+genus+species
genus	6	0	672	0	0.0	0.0	107	0.0	0.0	41				
species	7	0	196	0	0.0	0.0	74	0.0	0.0	49				
avg/sum	1.3	137305	41593	0	20.5	4.6	45.4	7.2	7.2	29.0			76.8	all but unassigned
avg/sum	0.8	225585	41593	0	30.4	4.0	39.9	18.8	6.3	25.5			84.4	all with unassigned

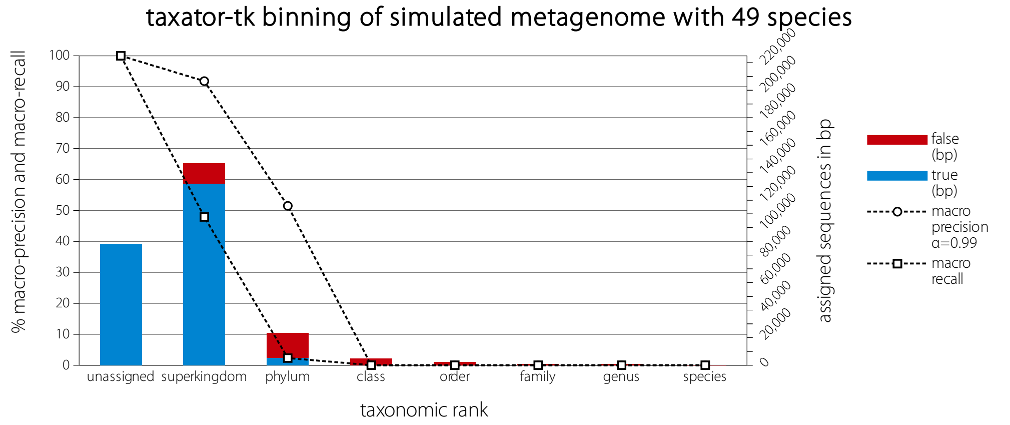

Supplementary Figure S13 - Taxator-tk binning of simulated metagenome with 49 species (simArt49e) **(h) new phylum scenario**

rank	depth	true (bp)	false (bp)	unknown (bp)	macro precision α=0.99	stdev	pred. bins	macro recall	stdev	real bins	sum true (bp)	sum false (bp)	overall prec.	description
unassigned	0	89957	0	0	100.0	0.0	1	100.0	0.0	1	338839	19454	94.6	root+superkingdom
superkingdom	1	124441	19454	0	89.7	0.0	1	45.6	45.0	2				
phylum	2	0	20771	0	0.0	0.0	6	0.0	0.0	20	0	31673	0.0	phylum+class+order
class	3	0	4678	0	0.0	0.0	15	0.0	0.0	23				
order	4	0	6224	0	0.0	0.0	27	0.0	0.0	32				
family	5	0	711	0	0.0	0.0	94	0.0	0.0	36	0	1653	0.0	family+genus+species
genus	6	0	735	0	0.0	0.0	141	0.0	0.0	41				
species	7	0	207	0	0.0	0.0	100	0.0	0.0	49				
avg/sum	1.3	124441	52780	0	12.8	0.0	54.9	6.5	6.4	29.0			70.2	all but unassigned
avg/sum	0.9	214398	52780	0	23.7	0.0	48.1	18.2	5.6	25.5			80.2	all with unassigned

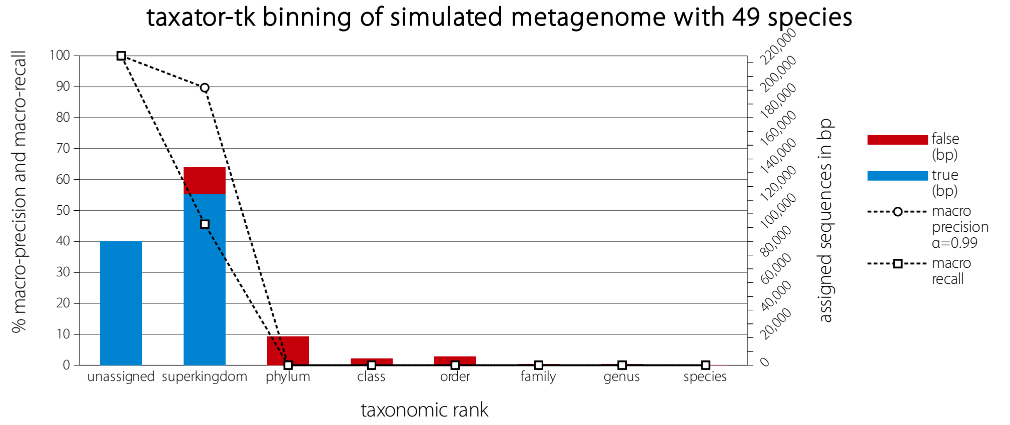

Supplementary Figure S14: Bin precision plots for 49 species simulated metagenomic sample (simArt49e)

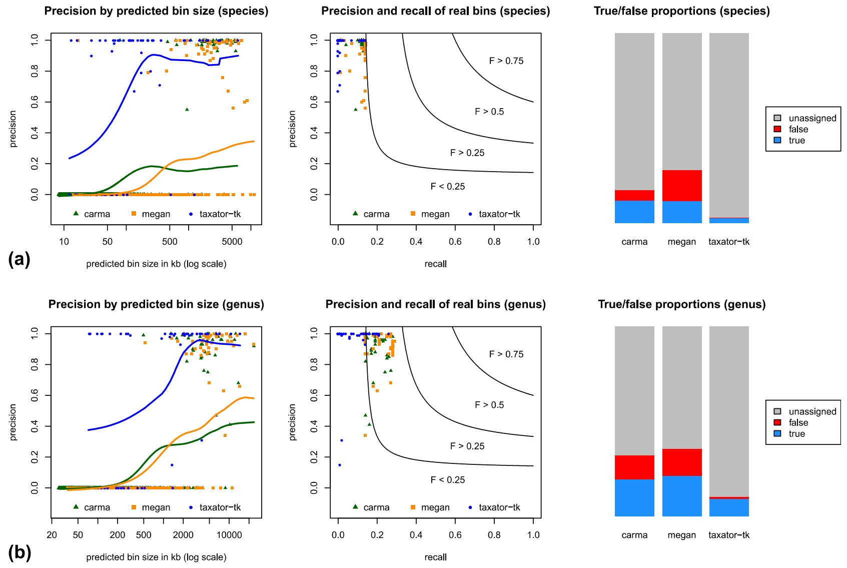

Supplementary Figure S14: Bin precision plots for 49 species simulated metagenomic sample (simArt49e)

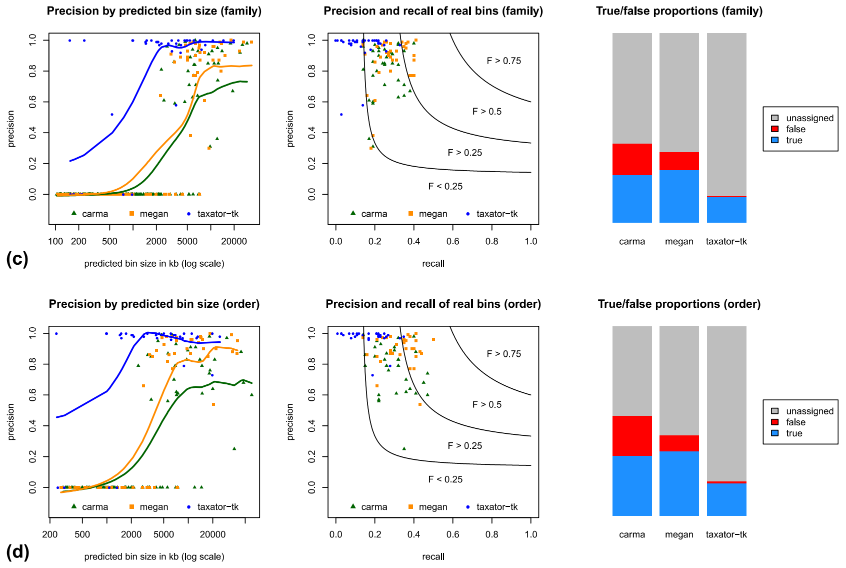

Supplementary Figure S14: Bin precision plots for 49 species simulated metagenomic sample (simArt49e)

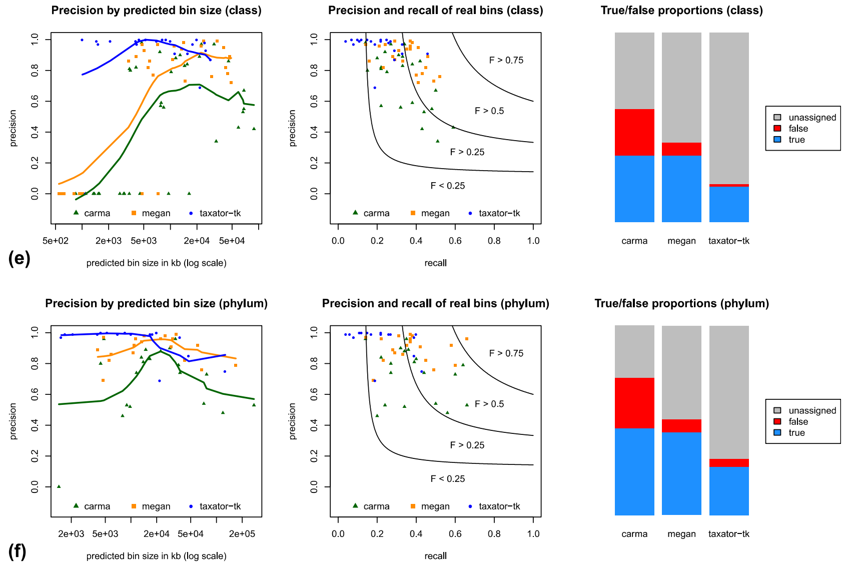

Supplementary Figure S14: Bin precision plots for 49 species simulated metagenomic sample (simArt49e)

Comparison of assignment quality of *CARMA3*, *MEGAN4* and *taxator-tk* for a simulated metagenome sample from a 49 species microbial community. Values are shown for the summary scenario (sum of all seven cross-validation scenarios), for assignments to the (a) species, (b) genus, (c) family, (d) order, (e) class and (f) phylum ranks, respectively. The first of each panels shows the precision and size for every predicted bin (after removing low abundance bins). The colored line shows a smoothed k-nearest-neighbor estimate of the mean precision as a function of predicted bin size using the R function *wapply* (width=0.3) followed by smooth.spline (df=10). The second panel for each rank shows bin precisions relative to recall. The F-score partitioning helps to identify similar quality bins if precision and recall are equally weighted, however we consider precision more important than recall. The third panel illustrates the total number of true (blue) and false (red) and unassigned (gray) portion of assignments at the respective ranks. Note that partially incorrect assignments are considered incorrect for the low ranking false part of the assignment and correct for the higher ranks.

Supplementary Figure S15: Taxonomic composition of SimMC/AMD

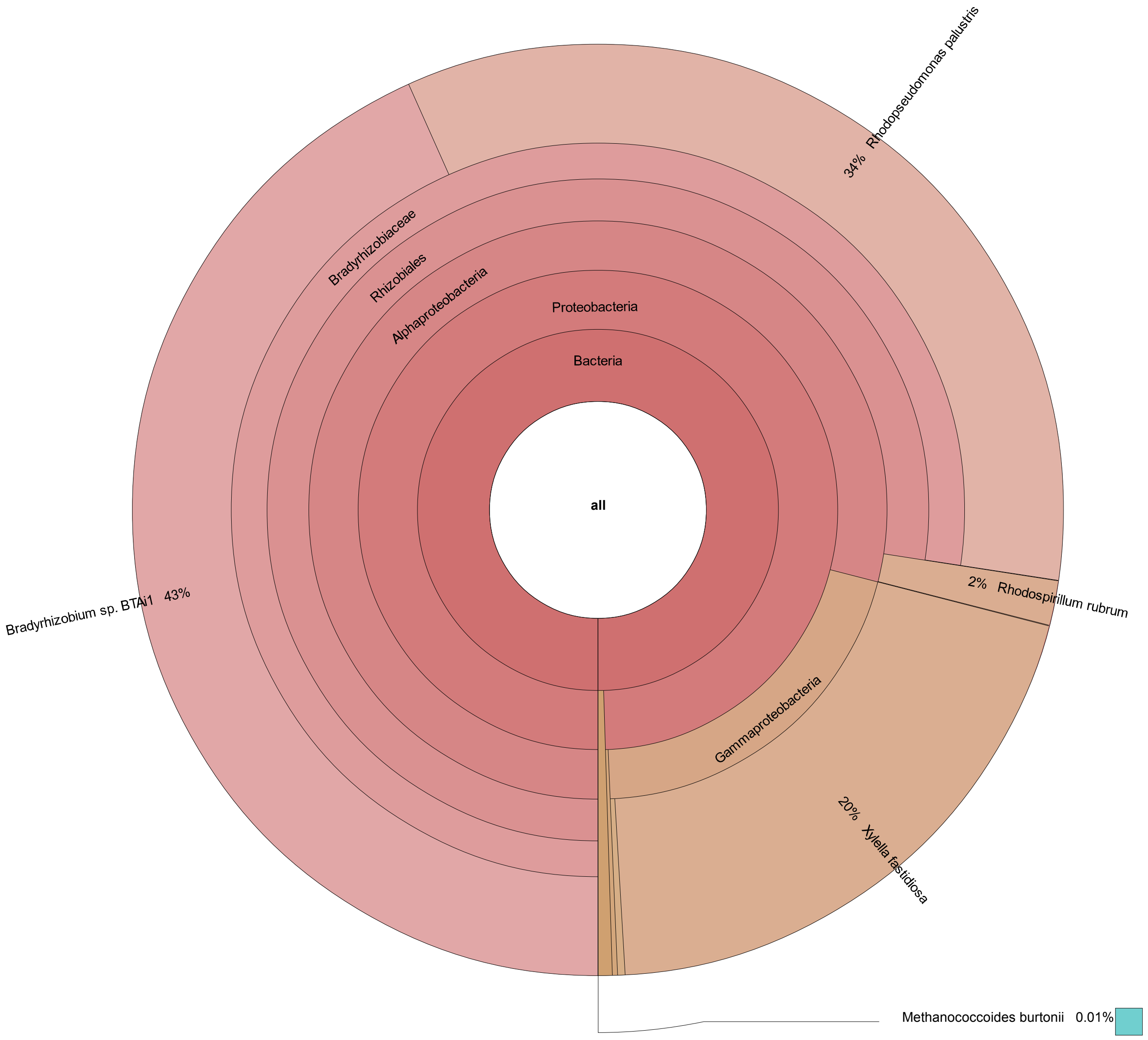

Taxonomic composition of the FAMeS simulated metagenome sample SimMC/AMD using Krona (Ondov et *al*., 2011). An interactive version can be found in the supplementary files (SimMC.krona.html). Abundance is measured in terms of accumulated contigs lengths.

Supplementary Figure S16: Taxonomic composition of SimHC/soil

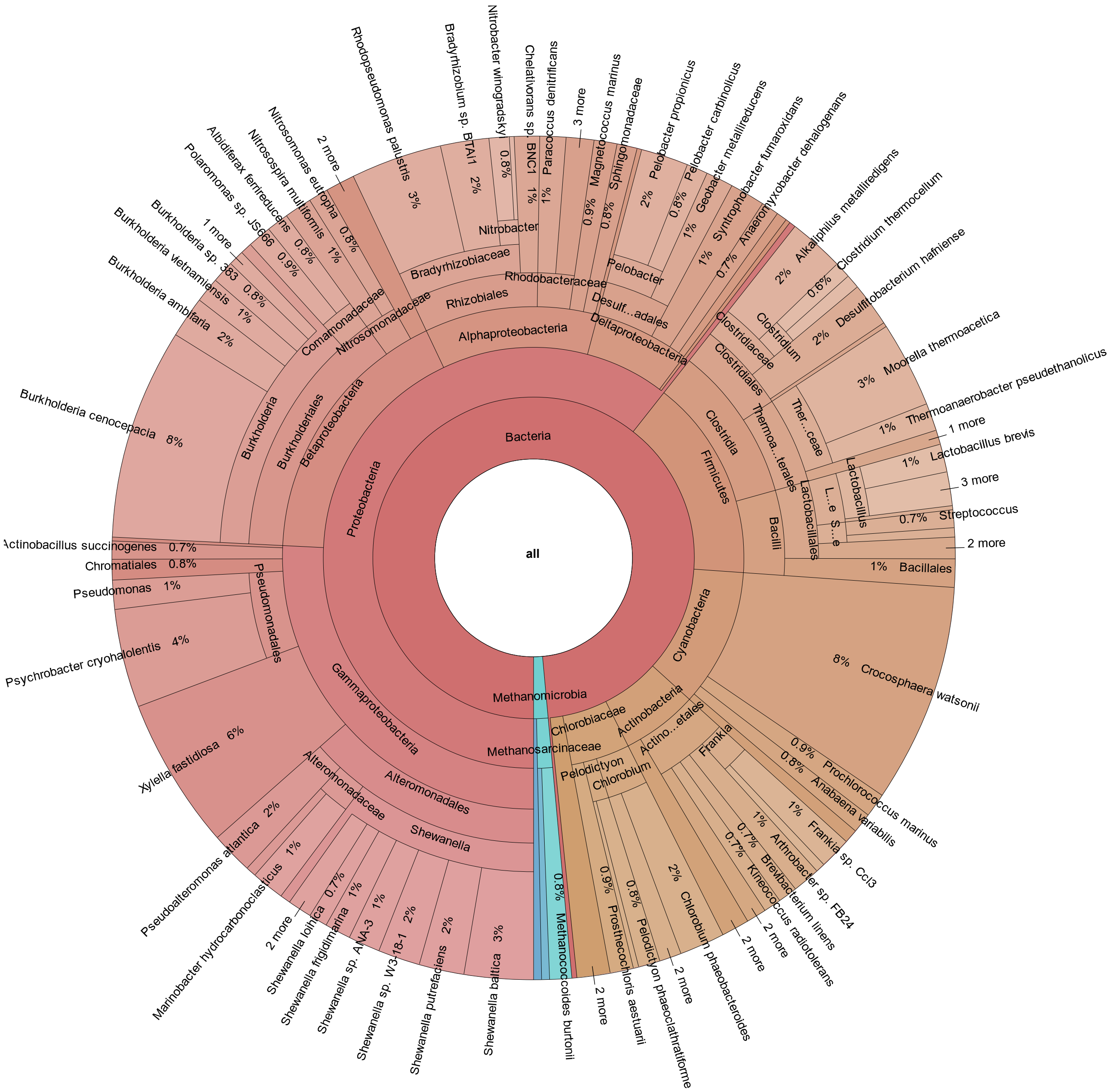

Taxonomic composition of the FAMeS simulated metagenome sample SimHC/soil using Krona (Ondov et *al*., 2011). An interactive version can be found in the supplementary files (SimHC.krona.html). Abundance is measured in terms of accumulated contigs lengths.

Supplementary Figure S17 - MEGAN binning for FAMeS SimMC **(a) summary scenario**

rank	depth	true (kb)	false (kb)	unknown (kb)	macro precision α=0.99	stdev	pred. bins	macro recall	stdev	real bins	sum true (kb)	sum false (kb)	overall prec.	description
unassigned	0	877.9	0.0	0	100.0	0.0	1	100.0	0.0	1	5735.3	7.5	99.9	root+superkingdom
superkingdom	1	2428.7	7.5	0	100.0	0.0	1	45.0	45.0	2				
phylum	2	2508.3	60.0	0	18.7	32.6	8	35.4	23.9	8	4604.0	1095.1	80.8	phylum+class+order
class	3	1611.6	389.1	0	14.8	29.4	17	24.3	19.7	12				
order	4	484.1	646.1	0	9.8	23.8	39	15.6	16.5	23				
family	5	1590.7	617.3	0	6.1	21.5	69	7.5	13.0	30	4734.4	3292.7	59.0	family+genus+species
genus	6	811.4	1102.6	0	3.9	18.0	131	3.5	7.2	37				
species	7	2332.3	1572.8	0	3.0	16.5	188	1.8	4.7	47				
avg/sum	3.3	11767.1	4395.3	0	22.3	20.2	64.7	19.0	18.6	22.7			72.8	all but unassigned
avg/sum	3.1	12645.0	4395.3	0	32.0	17.7	56.8	29.1	16.2	20.0			74.2	all with unassigned

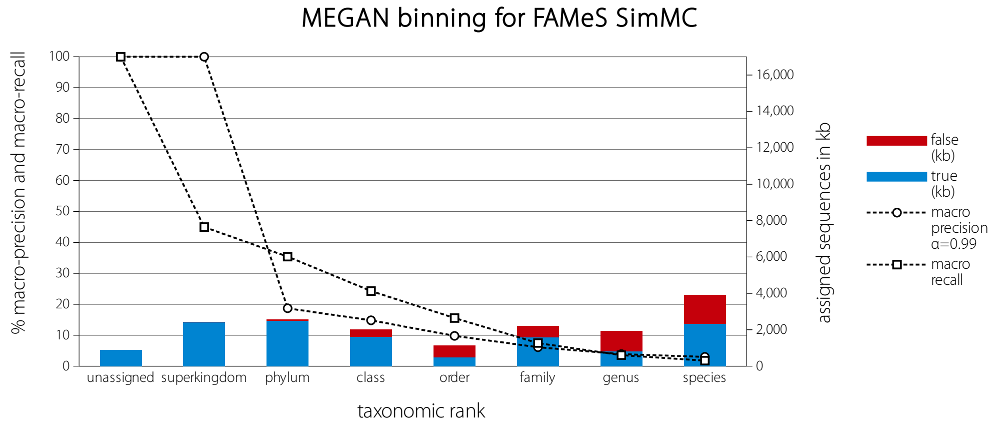

Supplementary Figure S17 - MEGAN binning for FAMeS SimMC **(b) all reference scenario**

rank	depth	true (kb)	false (kb)	unknown (kb)	macro precision α=0.99	stdev	pred. bins	macro recall	stdev	real bins	sum true (kb)	sum false (kb)	overall prec.	description
unassigned	0	2.03	0	0	100.0	0.0	1	100.0	0.0	1	20.91	0	100.0	root+superkingdom
superkingdom	1	9.44	0	0	100.0	0.0	1	50.0	50.0	2				
phylum	2	21.2	0	0	100.0	0.0	1	49.3	49.3	8	72.15	0	100.0	phylum+class+order
class	3	34.76	0	0	100.0	0.0	2	49.2	49.2	12				
order	4	16.19	0	0	100.0	0.0	3	33.9	46.5	23				
family	5	28.28	0	0	100.0	0.0	3	19.7	39.5	30	16956.66	0	100.0	family+genus+species
genus	6	602.48	0	0	100.0	0.0	4	18.7	38.7	37				
species	7	16325.9	0	0	100.0	0.0	4	12.5	32.6	47				
avg/sum	5.6	17038.25	0	0	100.0	0.0	2.6	33.3	43.7	22.7			100.0	all but unassigned
avg/sum	5.6	17040.28	0	0	100.0	0.0	2.4	41.7	38.2	20.0			100.0	all with unassigned

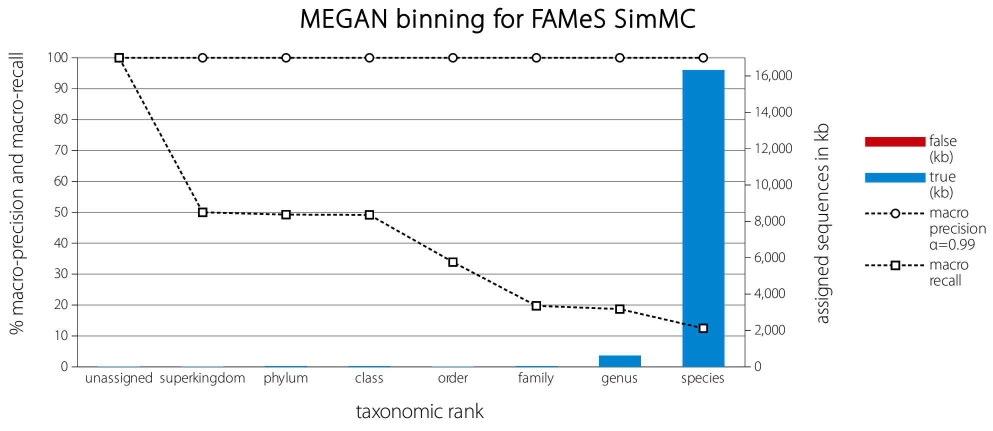

Supplementary Figure S17 - MEGAN binning for FAMeS SimMC **(c) new species scenario**

rank	depth	true (kb)	false (kb)	unknown (kb)	macro precision α=0.99	stdev	pred. bins	macro recall	stdev	real bins	sum true (kb)	sum false (kb)	overall prec.	description
unassigned	0	234.56	0	0	100.0	0.0	1	100.0	0.0	1	781.74	0	100.0	root+superkingdom
superkingdom	1	273.59	0	0	100.0	0.0	1	48.4	48.4	2				
phylum	2	1162.26	2.62	0	100.0	0.0	1	55.4	44.1	8	2423.08	125.55	95.1	phylum+class+order
class	3	683.93	59.82	0	66.8	44.7	3	45.6	37.9	12				
order	4	576.89	63.11	0	36.1	40.3	10	36.2	39.5	23				
family	5	4640.58	256.49	0	21.0	38.4	18	21.3	36.6	30	9717.71	4265.77	69.5	family+genus+species
genus	6	5077.13	1966.57	0	11.0	28.8	24	6.1	18.7	37				
species	7	0	2042.71	0	0.0	0.0	32	0.0	0.0	47				
avg/sum	5.0	12414.38	4391.32	0	47.8	21.7	12.7	30.4	32.2	22.7			73.9	all but unassigned
avg/sum	4.9	12648.94	4391.32	0	54.4	19.0	11.3	39.1	28.2	20.0			74.2	all with unassigned

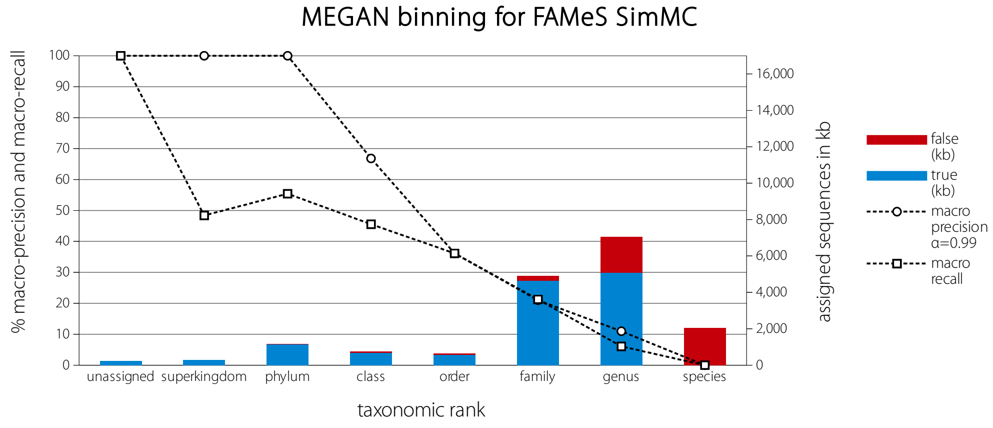

Supplementary Figure S17 - MEGAN binning for FAMeS SimMC **(d) new genus scenario**

rank	depth	true (kb)	false (kb)	unknown (kb)	macro precision α=0.99	stdev	pred. bins	macro recall	stdev	real bins	sum true (kb)	sum false (kb)	overall prec.	description
unassigned	0	358.62	0	0	100.0	0.0	1	100.0	0.0	1	1411.58	0	100.0	root+superkingdom
superkingdom	1	526.48	0	0	100.0	0.0	1	47.7	47.7	2				
phylum	2	1889.35	2.62	0	100.0	0.0	1	54.3	43.3	8	4564.44	220.44	95.4	phylum+class+order
class	3	1360.44	89.37	0	65.6	45.6	3	40.3	35.8	12				
order	4	1314.65	128.45	0	31.8	40.5	11	26.2	31.9	23				
family	5	6466.24	303.88	0	13.9	32.2	17	11.2	28.1	30	6466.24	4904.05	56.9	family+genus+species
genus	6	0	2126.87	0	0.0	0.0	39	0.0	0.0	37				
species	7	0	2473.3	0	0.0	0.0	45	0.0	0.0	47				
avg/sum	4.3	11557.16	5124.49	0	44.5	16.9	16.7	25.7	26.7	22.7			69.3	all but unassigned
avg/sum	4.2	11915.78	5124.49	0	51.4	14.8	14.8	34.9	23.3	20.0			69.9	all with unassigned

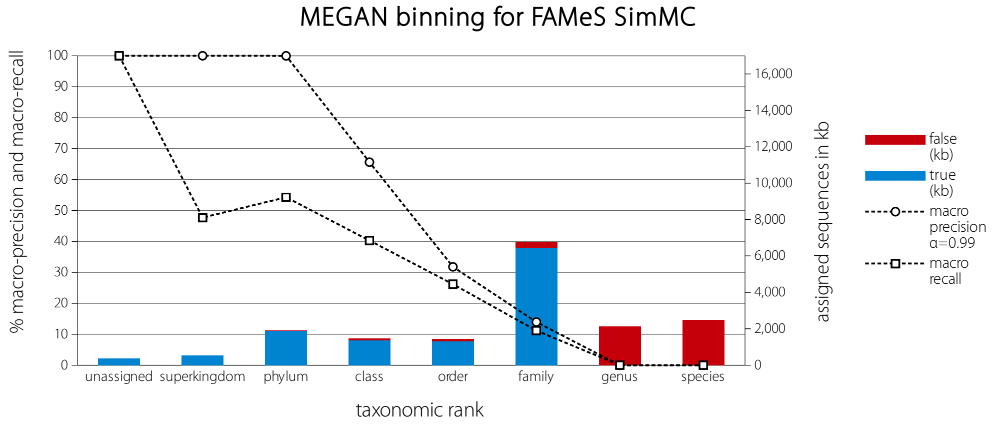

Supplementary Figure S17 - MEGAN binning for FAMeS SimMC **(e) new family scenario**

rank	depth	true (kb)	false (kb)	unknown (kb)	macro precision α=0.99	stdev	pred. bins	macro recall	stdev	real bins	sum true (kb)	sum false (kb)	overall prec.	description
unassigned	0	663.34	0	0	100.0	0.0	1	100.0	0.0	1	4214.02	0	100.0	root+superkingdom
superkingdom	1	1775.34	0	0	100.0	0.0	1	100.0	0.0	2				
phylum	2	4398.98	13.81	0	52.5	47.5	2	25.8	33.8	8	10748.33	470.17	95.8	phylum+class+order
class	3	4868.55	130.75	0	38.0	42.5	5	23.4	24.1	12				
order	4	1480.8	325.61	0	9.8	23.2	18	12.7	21.0	23				
family	5	0	1031.59	0	0.0	0.0	28	0.0	0.0	30	0	3383.11	0.0	family+genus+species
genus	6	0	839.84	0	0.0	0.0	47	0.0	0.0	37				
species	7	0	1511.68	0	0.0	0.0	47	0.0	0.0	47				
avg/sum	2.9	12523.67	3853.28	0	28.6	16.2	21.1	15.4	17.8	22.7			76.5	all but unassigned
avg/sum	2.8	13187.01	3853.28	0	37.5	14.1	18.6	26.0	15.6	20.0			77.4	all with unassigned

MEGAN binning for FAMeS SimMC

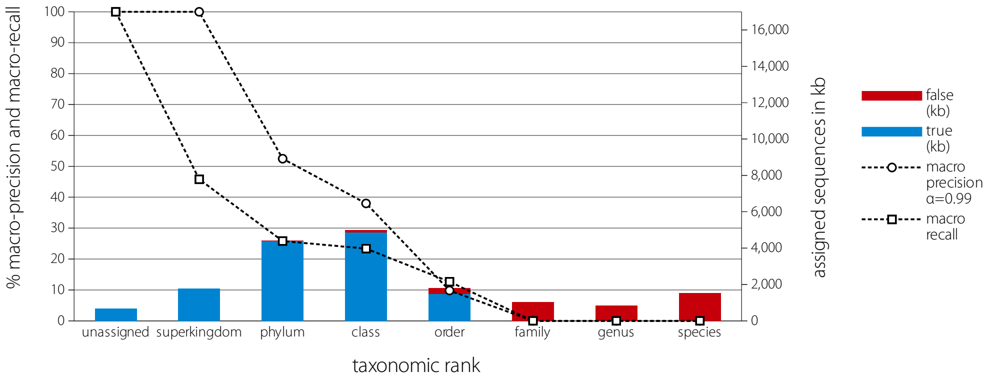

Supplementary Figure S17 - MEGAN binning for FAMeS SimMC **(f) new order scenario**

rank	depth	true (kb)	false (kb)	unknown (kb)	macro precision α=0.99	stdev	pred. bins	macro recall	stdev	real bins	sum true (kb)	sum false (kb)	overall prec.	description
unassigned	0	767.74	0	0	100.0	0.0	1	100.0	0.0	1	5311.66	0	100.0	root+superkingdom
superkingdom	1	2271.96	0	0	100.0	0.0	1	45.1	45.1	2				
phylum	2	5432.33	39.29	0	38.6	43.7	3	41.6	40.0	8	9765.97	793.72	92.5	phylum+class+order
class	3	4333.64	193	0	25.7	36.9	7	11.5	13.9	12				
order	4	0	561.43	0	0.0	0.0	21	0.0	0.0	23				
family	5	0	1006.4	0	0.0	0.0	30	0.0	0.0	30	0	3440.89	0.0	family+genus+species
genus	6	0	817.67	0	0.0	0.0	43	0.0	0.0	37				
species	7	0	1616.82	0	0.0	0.0	39	0.0	0.0	47				
avg/sum	2.7	12037.93	4234.61	0	23.5	11.5	20.6	14.0	14.1	22.7			74.0	all but unassigned
avg/sum	2.5	12805.67	4234.61	0	33.0	10.1	18.1	24.8	12.4	20.0			75.1	all with unassigned

MEGAN binning for FAMeS SimMC

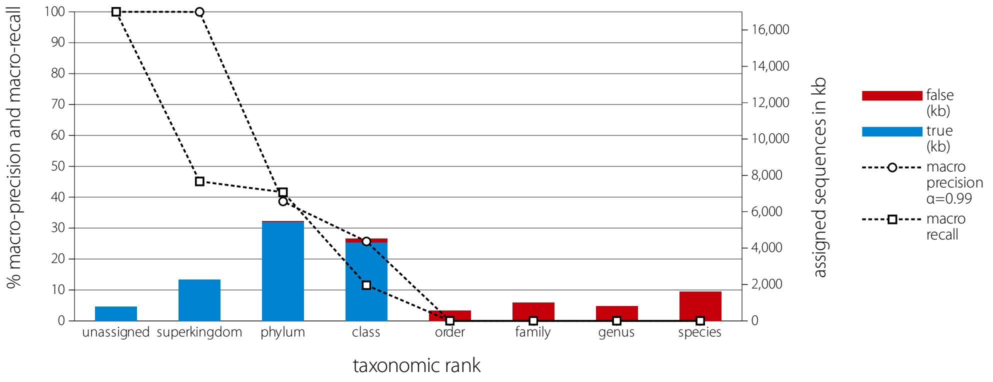

Supplementary Figure S17 - MEGAN binning for FAMeS SimMC **(g) new class scenario**

rank	depth	true (kb)	false (kb)	unknown (kb)	macro precision α=0.99	stdev	pred. bins	macro recall	stdev	real bins	sum true (kb)	sum false (kb)	overall prec.	description
unassigned	0	1274.66	0	0	100.0	0.0	1	100.0	0.0	1	11193.42	0	100.0	root+superkingdom
superkingdom	1	4959.38	0	0	100.0	0.0	1	42.4	42.4	2				
phylum	2	4654.01	87.88	0	15.0	34.7	7	21.4	34.5	8	4654.01	3194.98	59.3	phylum+class+order
class	3	0	1661.84	0	0.0	0.0	12	0.0	0.0	12				
order	4	0	1445.26	0	0.0	0.0	31	0.0	0.0	23				
family	5	0	960.19	0	0.0	0.0	40	0.0	0.0	30	0	2957.25	0.0	family+genus+species
genus	6	0	1106.84	0	0.0	0.0	57	0.0	0.0	37				
species	7	0	890.22	0	0.0	0.0	46	0.0	0.0	47				
avg/sum	2.5	9613.39	6152.23	0	16.4	5.0	27.7	9.1	11.0	22.7			61.0	all but unassigned
avg/sum	2.3	10888.05	6152.23	0	26.9	4.3	24.4	20.5	9.6	20.0			63.9	all with unassigned

MEGAN binning for FAMeS SimMC

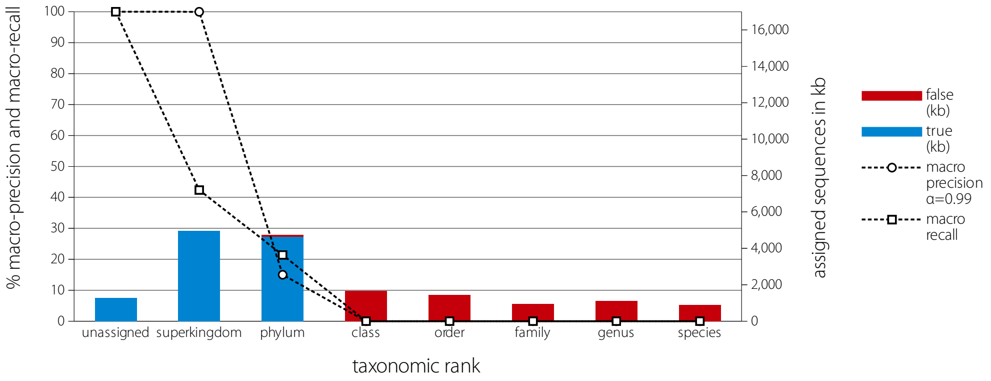

Supplementary Figure S17 - MEGAN binning for FAMeS SimMC **(h) new phylum scenario**

rank	depth	true (kb)	false (kb)	unknown (kb)	macro precision α=0.99	stdev	pred. bins	macro recall	stdev	real bins	sum true (kb)	sum false (kb)	overall prec.	description
unassigned	0	2844.15	0	0	100.0	0.0	1	100.0	0.0	1	17213.77	52.43	99.7	root+superkingdom
superkingdom	1	7184.81	52.43	0	100.0	0.0	1	35.3	35.3	2				
phylum	2	0	273.68	0	0.0	0.0	14	0.0	0.0	8	0	2861.02	0.0	phylum+class+order
class	3	0	588.78	0	0.0	0.0	26	0.0	0.0	12				
order	4	0	1998.56	0	0.0	0.0	40	0.0	0.0	23				
family	5	0	762.4	0	0.0	0.0	61	0.0	0.0	30	0	4097.87	0.0	family+genus+species
genus	6	0	860.34	0	0.0	0.0	72	0.0	0.0	37				
species	7	0	2475.13	0	0.0	0.0	69	0.0	0.0	47				
avg/sum	2.3	7184.81	7011.32	0	14.3	0.0	40.4	5.0	5.0	22.7			50.6	all but unassigned
avg/sum	1.8	10028.96	7011.32	0	25.0	0.0	35.5	16.9	4.4	20.0			58.9	all with unassigned

MEGAN binning for FAMeS SimMC

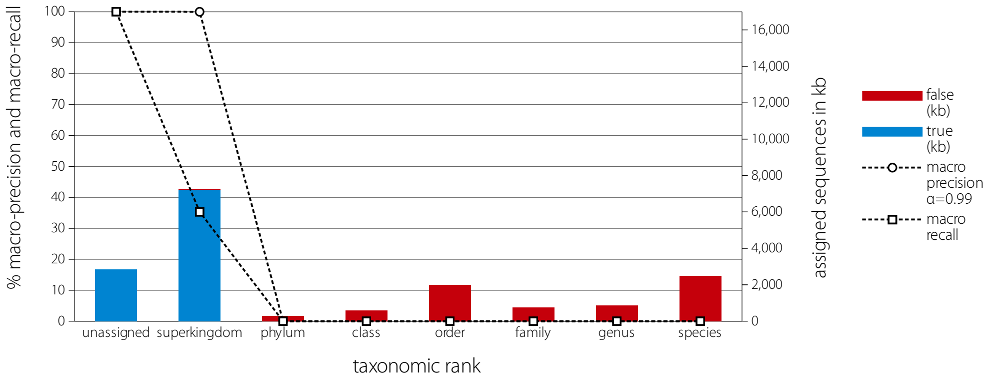

Supplementary Figure S18 - Taxator-tk binning for FAMeS SimMC

(a) summary scenario

rank	depth	true (kb)	false (kb)	unknown (kb)	macro precision α=0.99	stdev	pred. bins	macro recall	stdev	real bins	sum true (kb)	sum false (kb)	overall prec.	description
unassigned	0	2083.8	0.0	0	100.0	0.0	1	100.0	0.0	1	11492.5	0.6	100.0	root+superkingdom
superkingdom	1	4704.3	0.6	0	100.0	0.0	1	62.5	19.6	2				
phylum	2	3460.1	26.9	0	52.0	36.2	4	38.1	13.3	8	5881.5	466.4	92.7	phylum+class+order
class	3	1860.6	182.2	0	49.0	47.6	4	24.0	14.2	12				
order	4	560.8	257.2	0	40.7	41.6	15	18.6	12.0	23				
family	5	1573.3	89.4	0	22.8	38.3	19	12.8	10.8	30	3564.0	339.7	91.3	family+genus+species
genus	6	1012.7	196.9	0	37.5	45.7	19	8.0	7.9	37				
species	7	978.0	53.3	0	39.2	48.4	54	5.0	6.3	47				
avg/sum	2.6	14149.8	806.6	0	48.7	36.8	16.6	24.1	12.0	22.7			94.6	all but unassigned
avg/sum	2.3	16233.6	806.6	0	55.2	32.2	14.6	33.6	10.5	20.0			95.3	all with unassigned

taxator-tk binning for FAMeS SimMC

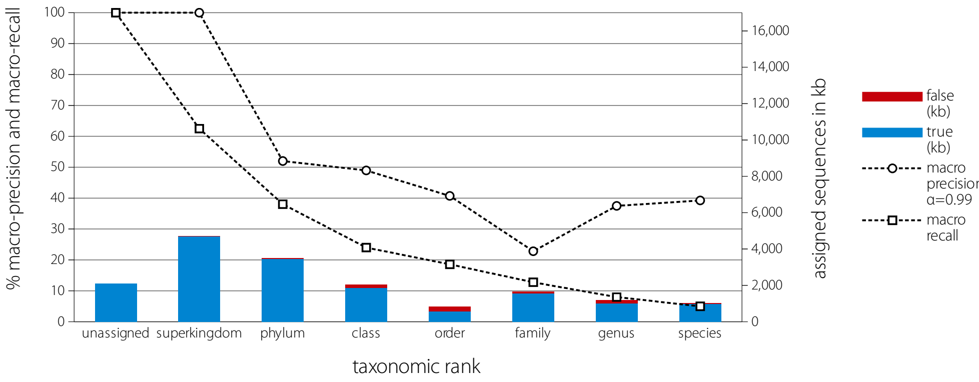

Supplementary Figure S18 - Taxator-tk binning for FAMeS SimMC

(b) all reference scenario

rank	depth	true (kb)	false (kb)	unknown (kb)	macro precision α=0.99	stdev	pred. bins	macro recall	stdev	real bins	sum true (kb)	sum false (kb)	overall prec.	description
unassigned	0	251.07	0	0	100.0	0.0	1	100.0	0.0	1	2857.85	0	100.0	root+superkingdom
superkingdom	1	1303.39	0	0	100.0	0.0	1	98.8	1.2	2				
phylum	2	1673.66	0	0	100.0	0.0	1	85.8	15.8	8	3450.59	0	100.0	phylum+class+order
class	3	1129.62	0	0	100.0	0.0	2	73.6	22.1	12				
order	4	647.31	0	0	100.0	0.0	4	68.7	29.4	23				
family	5	1728.38	0	0	100.0	0.0	3	59.0	42.0	30	12035.22	0	100.0	family+genus+species
genus	6	3460.93	0	0	100.0	0.0	5	47.1	43.6	37				
species	7	6845.91	0	0	100.0	0.0	5	34.9	44.2	47				
avg/sum	4.0	16789.2	0	0	100.0	0.0	3.0	66.8	28.3	22.7			100.0	all but unassigned
avg/sum	3.9	17040.27	0	0	100.0	0.0	2.8	71.0	24.8	20.0			100.0	all with unassigned

taxator-tk binning for FAMeS SimMC

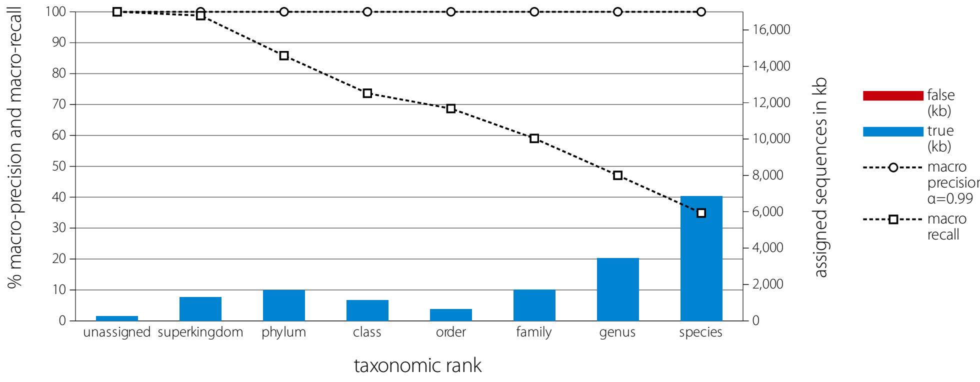

Supplementary Figure S18 - Taxator-tk binning for FAMeS SimMC

(c) new species scenario

rank	depth	true (kb)	false (kb)	unknown (kb)	macro precision α=0.99	stdev	pred. bins	macro recall	stdev	real bins	sum true (kb)	sum false (kb)	overall prec.	description
unassigned	0	1558.82	0	0	100.0	0.0	1	100.0	0.0	1	4785.94	0	100.0	root+superkingdom
superkingdom	1	1613.56	0	0	100.0	0.0	1	95.8	4.2	2				
phylum	2	2761.05	2.62	0	100.0	0.0	1	69.6	30.2	8	5592.14	50.24	99.1	phylum+class+order
class	3	1806.58	27.87	0	69.1	42.1	3	39.9	35.3	12				
order	4	1024.51	19.75	0	75.1	35.6	7	32.4	34.2	23				
family	5	3915.66	33.96	0	81.0	36.6	6	22.0	34.7	30	7543.8	681.73	91.7	family+genus+species
genus	6	3628.14	630.72	0	63.1	44.8	3	8.8	24.1	37				
species	7	0	17.05	0	0.0	0.0	11	0.0	0.0	47				
avg/sum	4.0	14749.5	731.97	0	69.7	22.7	4.6	38.4	23.2	22.7			95.3	all but unassigned
avg/sum	3.6	16308.32	731.97	0	73.5	19.9	4.1	46.1	20.3	20.0			95.7	all with unassigned

taxator-tk binning for FAMeS SimMC

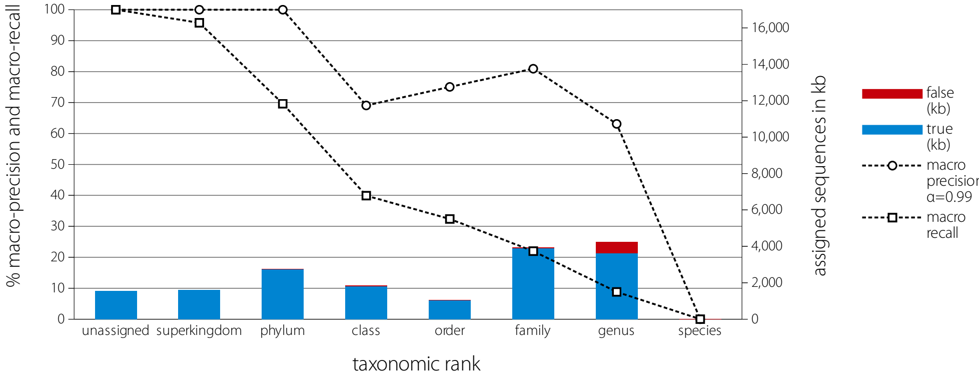

Supplementary Figure S18 - Taxator-tk binning for FAMeS SimMC

(d) new genus scenario

rank	depth	true (kb)	false (kb)	unknown (kb)	macro precision α=0.99	stdev	pred. bins	macro recall	stdev	real bins	sum true (kb)	sum false (kb)	overall prec.	description
unassigned	0	1745.53	0	0	100.0	0.0	1	100.0	0.0	1	5698.63	0	100.0	root+superkingdom
superkingdom	1	1976.55	0	0	100.0	0.0	1	94.6	5.4	2				
phylum	2	3398.87	2.62	0	100.0	0.0	1	63.2	32.7	8	6900.35	75.58	98.9	phylum+class+order
class	3	2312.46	32.37	0	66.9	44.9	3	30.2	32.2	12				
order	4	1189.02	40.59	0	55.5	42.8	7	22.2	32.0	23				
family	5	5368.74	54.74	0	50.0	46.3	7	8.3	22.3	30	5368.74	973.52	84.7	family+genus+species
genus	6	0	636.48	0	0.0	0.0	15	0.0	0.0	37				
species	7	0	282.3	0	0.0	0.0	9	0.0	0.0	47				
avg/sum	3.4	14245.64	1049.1	0	53.2	19.2	6.1	31.2	17.8	22.7			93.1	all but unassigned
avg/sum	3.1	15991.17	1049.1	0	59.0	16.8	5.5	39.8	15.6	20.0			93.8	all with unassigned

taxator-tk binning for FAMeS SimMC

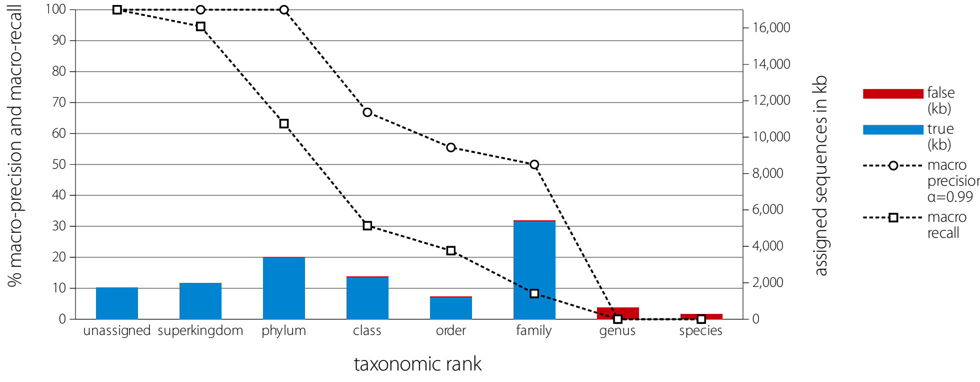

Supplementary Figure S18 - Taxator-tk binning for FAMeS SimMC **(e) new family scenario**

rank	depth	true (kb)	false (kb)	unknown (kb)	macro precision α=0.99	stdev	pred. bins	macro recall	stdev	real bins	sum true (kb)	sum false (kb)	overall prec.	description
unassigned	0	1444.21	0	0	100.0	0.0	1	100.0	0.0	1	9543.73	0	100.0	root+superkingdom
superkingdom	1	4049.76	0	0	100.0	0.0	1	42.8	42.8	2				
phylum	2	5463.82	11.04	0	100.0	0.0	1	31.1	36.5	8				
class	3	4607.11	80.28	0	61.6	43.9	3	21.0	31.0	12	11135.95	187.88	98.3	phylum+class+order
order	4	1065.02	96.56	0	20.6	37.4	18	6.8	17.0	23				
family	5	0	179.84	0	0.0	0.0	21	0.0	0.0	30				
genus	6	0	32.8	0	0.0	0.0	14	0.0	0.0	37	0	222.49	0.0	family+genus+species
species	7	0	9.85	0	0.0	0.0	7	0.0	0.0	47				
avg/sum	2.2	15185.71	410.37	0	40.3	11.6	9.3	14.5	18.2	22.7			97.4	all but unassigned
avg/sum	2.0	16629.92	410.37	0	47.8	10.2	8.3	25.2	15.9	20.0			97.6	all with unassigned

taxator-tk binning for FAMeS SimMC

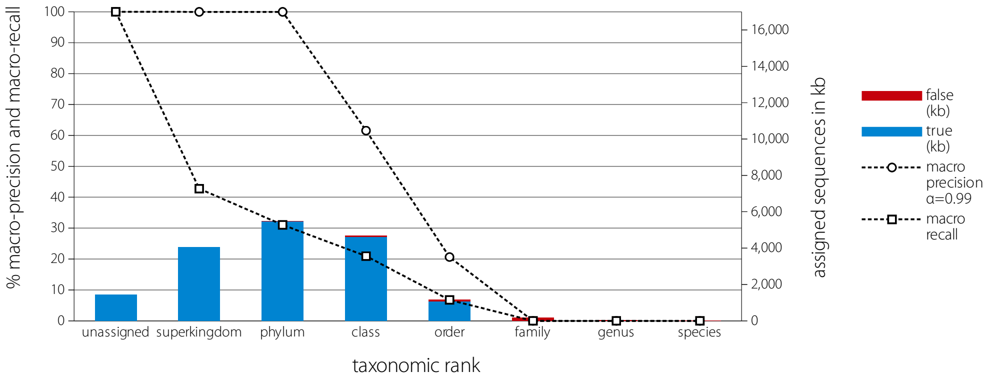

Supplementary Figure S18 - Taxator-tk binning for FAMeS SimMC **(f) new order scenario**

rank	depth	true (kb)	false (kb)	unknown (kb)	macro precision α=0.99	stdev	pred. bins	macro recall	stdev	real bins	sum true (kb)	sum false (kb)	overall prec.	description
unassigned	0	1665.24	0	0	100.0	0.0	1	100.0	0.0	1	11800.28	0	100.0	root+superkingdom
superkingdom	1	5067.52	0	0	100.0	0.0	1	41.4	41.4	2				
phylum	2	6525.5	14.12	0	100.0	0.0	1	13.5	20.3	8				
class	3	3168.64	86.66	0	45.2	45.6	4	3.3	6.6	12	9694.14	388.89	96.1	phylum+class+order
order	4	0	288.11	0	0.0	0.0	19	0.0	0.0	23				
family	5	0	169.88	0	0.0	0.0	17	0.0	0.0	30				
genus	6	0	40.01	0	0.0	0.0	14	0.0	0.0	37	0	224.49	0.0	family+genus+species
species	7	0	14.6	0	0.0	0.0	9	0.0	0.0	47				
avg/sum	2.0	14761.66	613.38	0	35.0	6.5	9.3	8.3	9.8	22.7			96.0	all but unassigned
avg/sum	1.8	16426.9	613.38	0	43.1	5.7	8.3	19.8	8.5	20.0			96.4	all with unassigned

taxator-tk binning for FAMeS SimMC

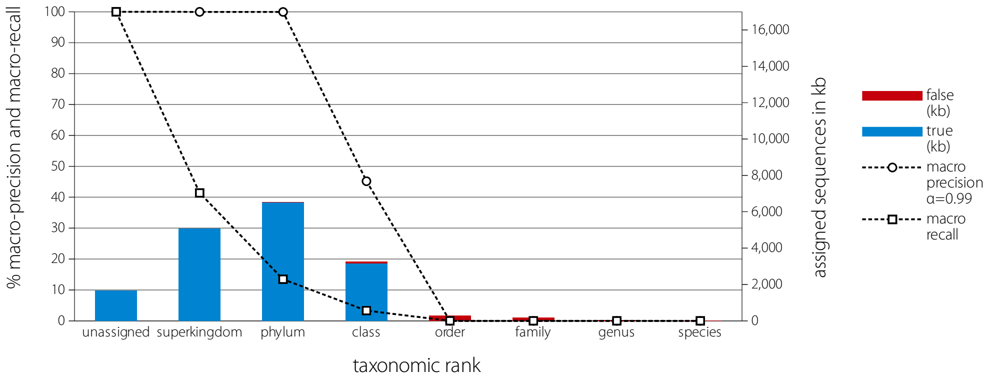

Supplementary Figure S18 - Taxator-tk binning for FAMeS SimMC **(g) new class scenario**

rank	depth	true (kb)	false (kb)	unknown (kb)	macro precision α=0.99	stdev	pred. bins	macro recall	stdev	real bins	sum true (kb)	sum false (kb)	overall prec.	description
unassigned	0	2853.36	0	0	100.0	0.0	1	100.0	0.0	1	19566.78	0	100.0	root+superkingdom
superkingdom	1	8356.71	0	0	100.0	0.0	1	36.9	36.9	2				
phylum	2	4397.58	25.84	0	33.3	47.1	3	3.2	8.5	8				
class	3	0	659.76	0	0.0	0.0	11	0.0	0.0	12	4397.58	1282.66	77.4	phylum+class+order
order	4	0	597.06	0	0.0	0.0	18	0.0	0.0	23				
family	5	0	108.29	0	0.0	0.0	21	0.0	0.0	30				
genus	6	0	23.82	0	0.0	0.0	14	0.0	0.0	37	0	149.96	0.0	family+genus+species
species	7	0	17.85	0	0.0	0.0	9	0.0	0.0	47				
avg/sum	1.6	12754.29	1432.62	0	19.0	6.7	11.0	5.7	6.5	22.7			89.9	all but unassigned
avg/sum	1.3	15607.65	1432.62	0	29.2	5.9	9.8	17.5	5.7	20.0			91.6	all with unassigned

taxator-tk binning for FAMeS SimMC

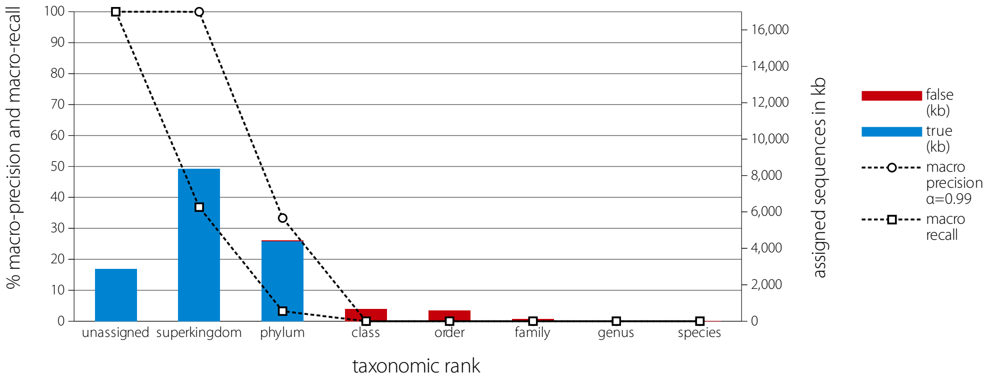

Supplementary Figure S18 - Taxator-tk binning for FAMeS SimMC **(h) new phylum scenario**

rank	depth	true (kb)	false (kb)	unknown (kb)	macro precision α=0.99	stdev	pred. bins	macro recall	stdev	real bins	sum true (kb)	sum false (kb)	overall prec.	description
unassigned	0	5068.27	0	0	100.0	0.0	1	100.0	0.0	1	26194.15	4.05	100.0	root+superkingdom
superkingdom	1	10562.94	4.05	0	100.0	0.0	1	27.3	27.3	2				
phylum	2	0	132.26	0	0.0	0.0	10	0.0	0.0	8				
class	3	0	388.44	0	0.0	0.0	18	0.0	0.0	12	0	1279.35	0.0	phylum+class+order
order	4	0	758.65	0	0.0	0.0	19	0.0	0.0	23				
family	5	0	79.35	0	0.0	0.0	24	0.0	0.0	30				
genus	6	0	14.55	0	0.0	0.0	17	0.0	0.0	37	0	125.67	0.0	family+genus+species
species	7	0	31.77	0	0.0	0.0	14	0.0	0.0	47				
avg/sum	1.3	10562.94	1409.07	0	14.3	0.0	14.7	3.9	3.9	22.7			88.2	all but unassigned
avg/sum	0.9	15631.21	1409.07	0	25.0	0.0	13.0	15.9	3.4	20.0			91.7	all with unassigned

taxator-tk binning for FAMeS SimMC

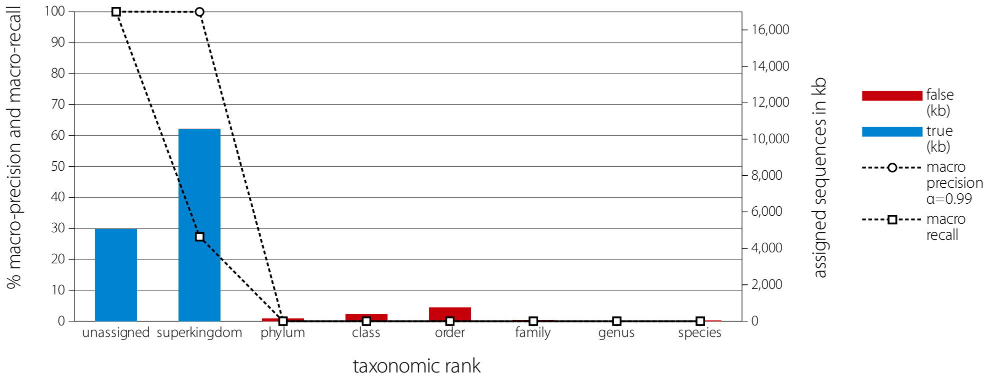

Supplementary Figure S19 - MEGAN binning for FAMeS SimHC **(a) summary scenario**

rank	depth	true (bp)	false (bp)	unknown (bp)	macro precision α=0.99	stdev	pred. bins	macro recall	stdev	real bins	sum true (bp)	sum false (bp)	overall prec	description
unassigned	0	135097.7	0.0	0.0	100.0	0.0	1	100.0	0.0	1	504932.0	1240.4	99.8	root+superkingdom
superkingdom	1	184917.1	1240.4	0.0	99.5	0.0	1	65.7	21.3	2				
phylum	2	126186.3	17996.0	0.0	54.3	45.2	10	39.8	24.2	8	297237.9	124491.1	70.5	phylum+class+order
class	3	105637.4	52554.0	2704.9	61.0	36.9	12	32.6	19.7	12				
order	4	65414.1	53941.1	0.0	60.5	41.5	27	17.4	17.2	36				
family	5	47775.7	34408.6	0.0	68.1	38.6	36	13.1	13.3	52	228155.2	135050.2	62.8	family+genus+species
genus	6	70368.1	42132.0	382.3	65.3	43.0	47	8.4	9.4	72				
species	7	110011.3	58509.7	356.5	69.9	45.6	47	4.7	6.5	96				
avg/sum	3.1	710310.2	260781.8	3443.6	68.4	35.8	25.7	25.9	15.9	39.7			73.1	all but unassigned
avg/sum	2.7	845407.9	260781.8	3443.6	72.3	31.3	22.6	35.2	14.0	34.9			76.4	all with unassigned

MEGAN binning for FAMeS SimHC

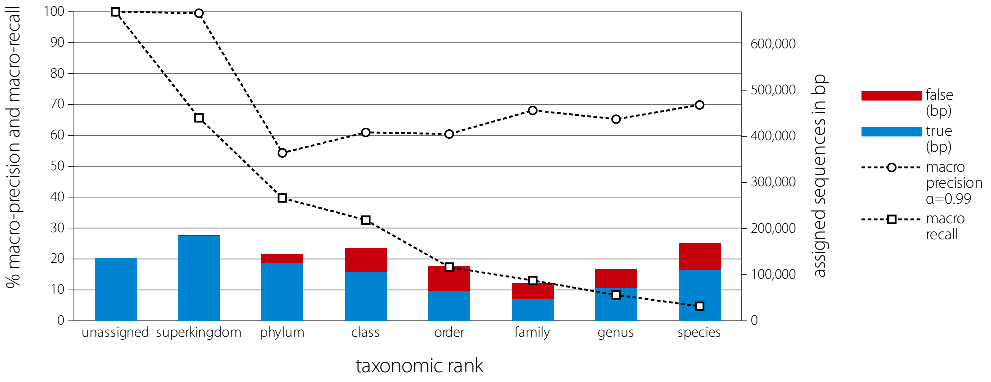

Supplementary Figure S19 - MEGAN binning for FAMeS SimHC **(b) all reference scenario**

rank	depth	true (bp)	false (bp)	unknown (bp)	macro precision α=0.99	stdev	pred. bins	macro recall	stdev	real bins	sum true (bp)	sum false (bp)	overall prec	description
unassigned	0	0	0	0	100.0	0.0	1	100.0	0.0	1	29660	0	100.0	root+superkingdom
superkingdom	1	14830	0	0	100.0	0.0	2	100.0	0.0	2				
phylum	2	27071	0	0	99.6	1.0	6	74.9	43.2	8	123849	0	100.0	phylum+class+order
class	3	58344	0	0	99.6	0.8	9	74.0	42.8	12				
order	4	38434	0	0	99.8	0.7	20	55.0	49.3	36				
family	5	58139	0	0	99.4	3.1	29	54.8	49.1	52	942014	0	100.0	family+genus+species
genus	6	223807	0	2676	99.5	2.8	34	46.7	49.5	72				
species	7	660068	0	2139	99.5	2.9	33	32.8	45.8	96				
avg/sum	4.8	1080693	0	4815	99.6	1.6	19.0	62.6	39.9	39.7			100.0	all but unassigned
avg/sum	4.8	1080693	0	4815	99.7	1.4	16.8	67.3	35.0	34.9			100.0	all with unassigned

MEGAN binning for FAMeS SimHC

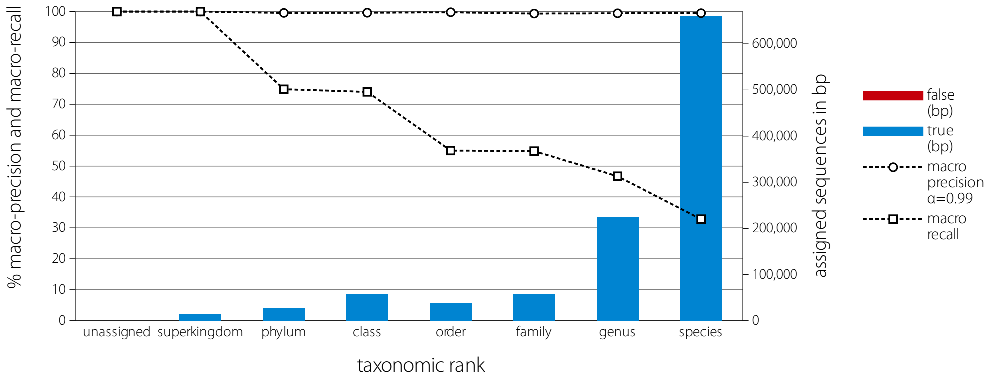

Supplementary Figure S19 - MEGAN binning for FAMeS SimHC **(c) new species scenario**

rank	depth	true (bp)	false (bp)	unknown (bp)	macro precision α=0.99	stdev	pred. bins	macro recall	stdev	real bins	sum true (bp)	sum false (bp)	overall prec	description
unassigned	0	53833	0	0	100.0	0.0	1	100.0	0.0	1	196059	0	100.0	root+superkingdom
superkingdom	1	71113	0	0	99.9	0.1	2	86.2	8.4	2				
phylum	2	85694	0	0	99.7	0.5	6	65.7	38.2	8	311019	25259	92.5	phylum+class+order
class	3	111601	14697	0	96.4	4.6	9	57.5	34.8	12				
order	4	113724	10562	0	86.9	29.7	18	33.0	38.4	36				
family	5	140308	25633	0	83.4	27.0	20	24.5	35.6	52	409078	215206	65.5	family+genus+species
genus	6	268770	68288	0	56.8	44.8	17	11.9	29.1	72				
species	7	0	121285	0	0.0	0.0	8	0.0	0.0	96				
avg/sum	4.4	791210	240465	0	74.7	15.2	11.4	39.8	26.4	39.7			76.7	all but unassigned
avg/sum	4.1	845043	240465	0	77.9	13.3	10.1	47.3	23.1	34.9			77.8	all with unassigned

MEGAN binning for FAMeS SimHC

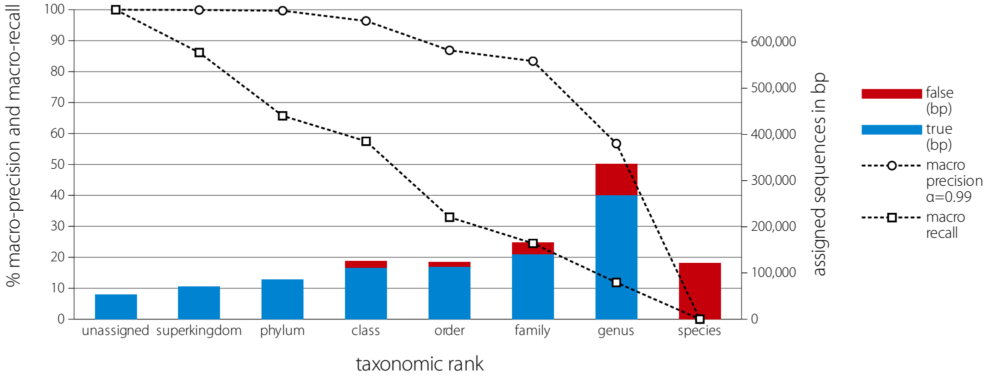

Supplementary Figure S19 - MEGAN binning for FAMeS SimHC **(d) new genus scenario**

rank	depth	true (bp)	false (bp)	unknown (bp)	macro precision α=0.99	stdev	pred. bins	macro recall	stdev	real bins	sum true (bp)	sum false (bp)	overall prec	description
unassigned	0	60008	0	0	100.0	0.0	1	100.0	0.0	1	266134	0	100.0	root+superkingdom
superkingdom	1	103063	0	0	99.9	0.1	2	85.7	8.0	2				
phylum	2	142075	0	0	98.3	2.3	6	62.6	36.7	8	481779	57207	89.4	phylum+class+order
class	3	171151	27602	0	92.0	6.7	9	49.7	31.2	12				
order	4	168553	29605	0	73.6	34.2	18	25.2	32.4	36				
family	5	135983	53126	0	55.4	39.5	16	12.0	25.2	52	135983	247468	35.5	family+genus+species
genus	6	0	123176	0	0.0	0.0	12	0.0	0.0	72				
species	7	0	71166	0	0.0	0.0	5	0.0	0.0	96				
avg/sum	3.6	720825	304675	0	59.9	11.8	9.7	33.6	19.1	39.7			70.3	all but unassigned
avg/sum	3.4	780833	304675	0	64.9	10.4	8.6	41.9	16.7	34.9			71.9	all with unassigned

MEGAN binning for FAMeS SimHC

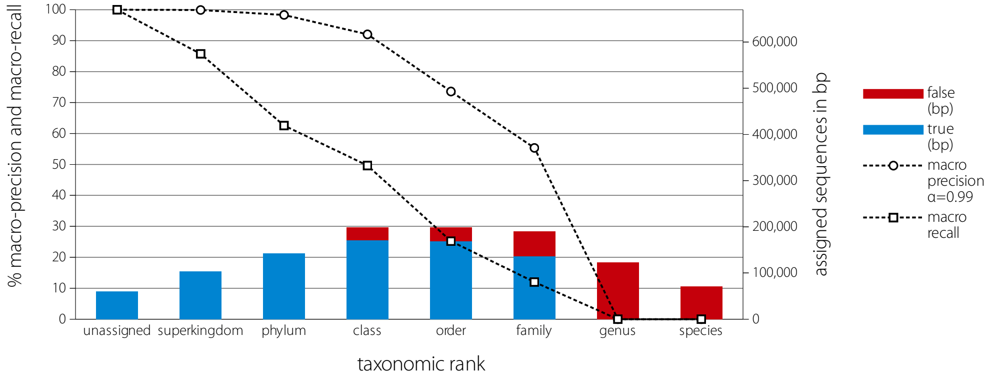

Supplementary Figure S19 - MEGAN binning for FAMeS SimHC **(e) new family scenario**

rank	depth	true (bp)	false (bp)	unknown (bp)	macro precision α=0.99	stdev	pred. bins	macro recall	stdev	real bins	sum true (bp)	sum false (bp)	overall prec	description
unassigned	0	180596	0	0	100.0	0.0	1	100.0	0.0	1	498492	1776	99.6	root+superkingdom
superkingdom	1	158948	1776	0	99.7	0.3	2	69.3	13.7	2				
phylum	2	202392	3264	0	95.4	4.8	5	35.9	33.9	8	540433	103443	83.9	phylum+class+order
class	3	200853	52078	0	82.6	9.8	7	30.3	27.9	12				
order	4	137188	48101	0	56.7	33.0	11	8.4	17.2	36				
family	5	0	57702	0	0.0	0.0	8	0.0	0.0	52	0	100312	0.0	family+genus+species
genus	6	0	31996	0	0.0	0.0	3	0.0	0.0	72				
species	7	0	10614	0	0.0	0.0	1	0.0	0.0	96				
avg/sum	2.9	699381	205531	0	47.8	6.8	5.3	20.5	13.2	39.7			77.3	all but unassigned
avg/sum	2.4	879977	205531	0	54.3	6.0	4.8	30.5	11.6	34.9			81.1	all with unassigned

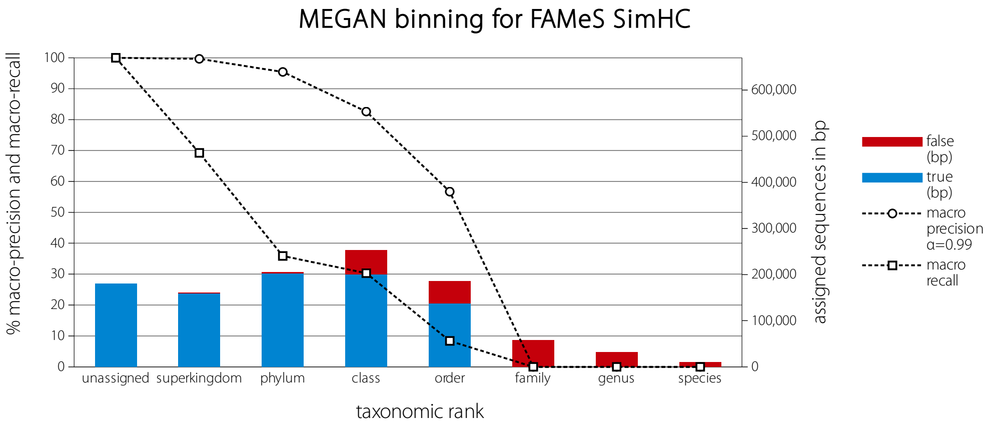

Supplementary Figure S19 - MEGAN binning for FAMeS SimHC **(f) new order scenario**

rank	depth	true (bp)	false (bp)	unknown (bp)	macro precision α=0.99	stdev	pred. bins	macro recall	stdev	real bins	sum true (bp)	sum false (bp)	overall prec	description
unassigned	0	115421	0	0	100.0	0.0	1	100.0	0.0	1	558349	1776	99.7	root+superkingdom
superkingdom	1	221464	1776	0	99.4	0.0	1	44.3	44.3	2				
phylum	2	250454	8635	0	84.2	18.2	4	29.2	34.1	8	447967	169429	72.6	phylum+class+order
class	3	197513	58615	1390	62.1	16.6	7	16.7	18.1	12				
order	4	0	102179	0	0.0	0.0	12	0.0	0.0	36				
family	5	0	40254	0	0.0	0.0	6	0.0	0.0	52	0	128061	0.0	family+genus+species
genus	6	0	35656	0	0.0	0.0	5	0.0	0.0	72				
species	7	0	52151	0	0.0	0.0	2	0.0	0.0	96				
avg/sum	2.5	669431	299266	1390	35.1	5.0	5.3	12.9	13.8	39.7			69.1	all but unassigned
avg/sum	2.3	784852	299266	1390	43.2	4.3	4.8	23.8	12.1	34.9			72.4	all with unassigned

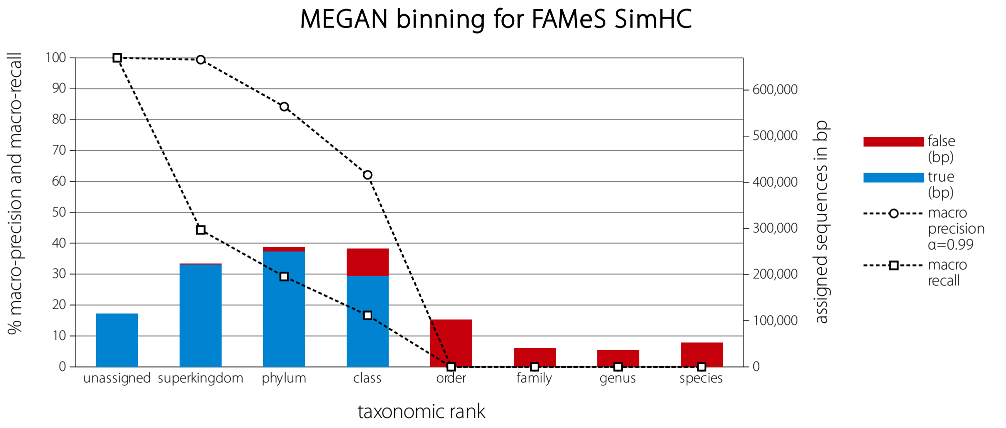

Supplementary Figure S19 - MEGAN binning for FAMeS SimHC **(g) new class scenario**

rank	depth	true (bp)	false (bp)	unknown (bp)	macro precision α=0.99	stdev	pred. bins	macro recall	stdev	real bins	sum true (bp)	sum false (bp)	overall prec	description
unassigned	0	271642	0	0	100.0	0.0	1	100.0	0.0	1	958974	1776	99.8	root+superkingdom
superkingdom	1	343666	1776	0	99.3	0.0	1	37.7	37.7	2				
phylum	2	175618	16711	0	53.5	38.2	3	10.0	18.1	8	175618	235397	42.7	phylum+class+order
class	3	0	146037	0	0.0	0.0	7	0.0	0.0	12				
order	4	0	72649	0	0.0	0.0	9	0.0	0.0	36				
family	5	0	34445	0	0.0	0.0	5	0.0	0.0	52	0	57409	0.0	family+genus+species
genus	6	0	22964	0	0.0	0.0	2	0.0	0.0	72				
species	7							0.0	0.0	96				
avg/sum	2.2	519284	294582	0	25.5	6.4	4.5	6.8	8.0	39.7			63.8	all but unassigned
avg/sum	1.6	790926	294582	0	36.1	5.5	4.0	18.5	7.0	34.9			72.9	all with unassigned

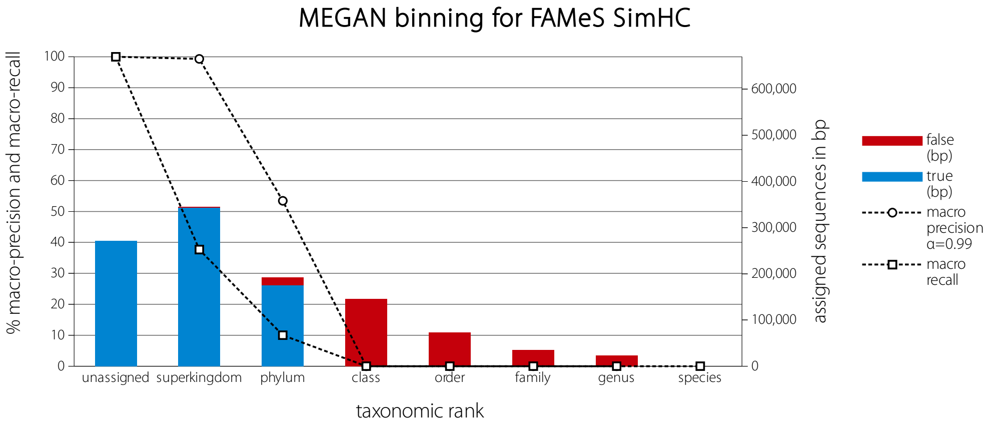

Supplementary Figure S19 - MEGAN binning for FAMeS SimHC **(h) new phylum scenario**

rank	depth	true (bp)	false (bp)	unknown (bp)	macro precision α=0.99	stdev	pred. bins	macro recall	stdev	real bins	sum true (bp)	sum false (bp)	overall prec	description
unassigned	0	264184	0	0	100.0	0.0	1	100.0	0.0	1	1026856	3355	99.7	root+superkingdom
superkingdom	1	381336	3355	0	98.8	0.0	1	36.7	36.7	2				
phylum	2	0	97362	0	0.0	0.0	12	0.0	0.0	8	0	280703	0.0	phylum+class+order
class	3	0	68849	17544	0.0	0.0	11	0.0	0.0	12				
order	4	0	114492	0	0.0	0.0	10	0.0	0.0	36				
family	5	0	29700	0	0.0	0.0	6	0.0	0.0	52	0	138386	0.0	family+genus+species
genus	6	0	12844	0	0.0	0.0	4	0.0	0.0	72				
species	7	0	95842	0	0.0	0.0	5	0.0	0.0	96				
avg/sum	2.1	381336	422444	17544	14.1	0.0	7.0	5.2	5.2	39.7			47.4	all but unassigned
avg/sum	1.5	645520	422444	17544	24.9	0.0	6.3	17.1	4.6	34.9			60.4	all with unassigned

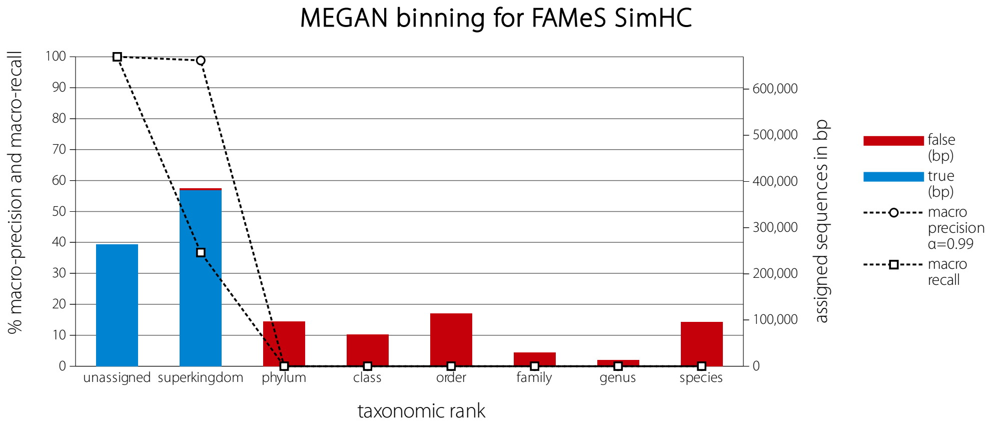

Supplementary Figure S20 - Taxator-tk binning for FAMeS SimHC **(a) summary scenario**

rank	depth	true (bp)	false (bp)	unknown (bp)	macro precision α=0.99	stdev	pred. bins	macro recall	stdev	real bins	sum true (bp)	sum false (bp)	overall prec.	description
unassigned	0	213863.4	0.0	0.0	100.0	0.0	1	100.0	0.0	1	993072.9	1240.4	99.9	root+superkingdom
superkingdom	1	389604.7	1240.4	0.0	99.9	0.1	2	64.1	14.9	2				
phylum	2	144608.1	5934.9	0.0	96.5	5.1	7	34.9	7.9	8				
class	3	74893.3	12043.4	757.0	92.7	8.4	11	24.4	10.3	12	259408.7	32094.3	89.0	phylum+class+order
order	4	39907.3	14116.0	0.0	65.6	44.8	47	15.3	10.0	36				
family	5	31822.9	11110.7	0.0	68.9	43.0	58	11.7	9.7	52				
genus	6	59831.9	13687.3	382.3	75.1	40.3	68	8.2	7.5	72	153059.7	34715.1	81.5	family+genus+species
species	7	61405.0	9917.1	382.3	76.6	41.5	66	4.1	4.8	96				
avg/sum	2.3	802073.1	68049.9	1521.6	82.2	26.2	37.0	23.2	9.3	39.7			92.2	all but unassigned
avg/sum	1.8	1015936.6	68049.9	1521.6	84.4	22.9	32.5	32.8	8.1	34.9			93.7	all with unassigned

taxator-tk binning for FAMeS SimHC

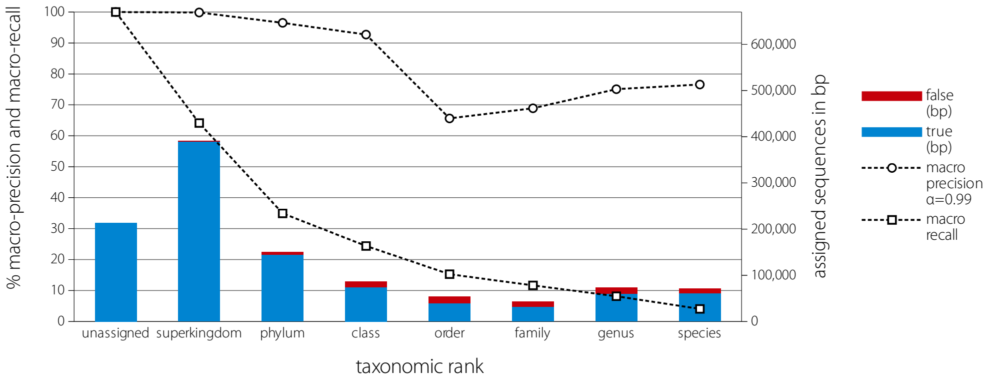

Supplementary Figure S20 - Taxator-tk binning for FAMeS SimHC **(b) all reference scenario**

rank	depth	true (bp)	false (bp)	unknown (bp)	macro precision α=0.99	stdev	pred. bins	macro recall	stdev	real bins	sum true (bp)	sum false (bp)	overall prec.	description
unassigned	0	47885		0	100.0	0.0	1	100.0	0.0	1	375435	0	100.0	root+superkingdom
superkingdom	1	163775	(bp)	0	100.0	0.0	2	97.6	2.4	2				
phylum	2	70870	0	0	99.4	1.2	6	70.3	28.5	8				
class	3	65908	0	2139	99.5	1.1	9	59.3	28.9	12	185117	0	100.0	phylum+class+order
order	4	48339	0	0	99.9	0.4	32	55.5	30.6	36				
family	5	72448	0	0	99.9	0.4	43	49.9	34.0	52				
genus	6	179113	0	2676	100.0	0.3	55	43.1	35.7	72	681396	2520	99.6	family+genus+species
species	7	429835	2520	0	100.0	0.3	52	28.5	33.5	96				
avg/sum	3.6	1030288	2520	4815	99.8	0.5	28.4	57.8	27.7	39.7			99.8	all but unassigned
avg/sum	3.3	1078173	2520	4815	99.8	0.5	25.0	63.0	24.2	34.9			99.8	all with unassigned

taxator-tk binning for FAMeS SimHC

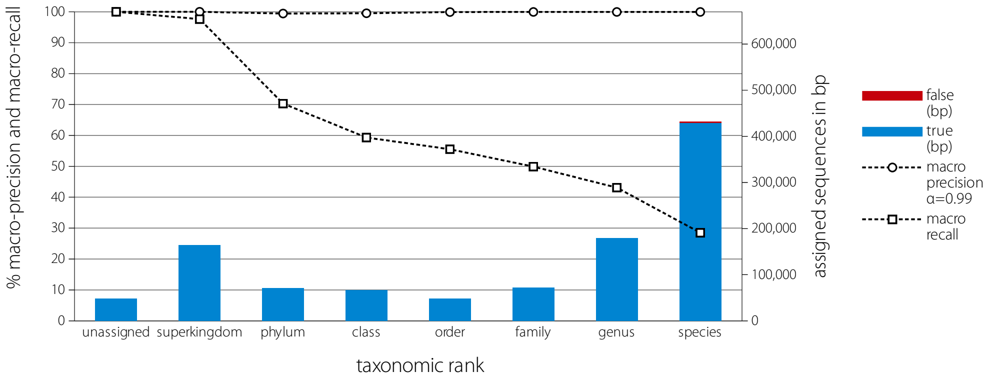

Supplementary Figure S20 - Taxator-tk binning for FAMeS SimHC **(c) new species scenario**

rank	depth	true (bp)	false (bp)	unknown (bp)	macro precision α=0.99	stdev	pred. bins	macro recall	stdev	real bins	sum true (bp)	sum false (bp)	overall prec.	description
unassigned	0	92565		0	100.0	0.0	1	100.0	0.0	1	608487	0	100.0	root+superkingdom
superkingdom	1	257961	(bp)	0	100.0	0.0	2	84.1	6.4	2				
phylum	2	152494	0	0	100.0	0.0	7	62.6	26.6	8				
class	3	94075	0	0	100.0	0.0	10	48.6	28.5	12	343148	6930	98.0	phylum+class+order
order	4	96579	6930	0	88.6	28.8	26	30.0	30.1	36				
family	5	80651	10331	0	86.9	30.4	30	23.7	31.8	52				
genus	6	239710	34526	0	65.5	46.1	31	13.9	26.1	72	320361	64543	83.2	family+genus+species
species	7	0	19686	0	0.0	0.0	9	0.0	0.0	96				
avg/sum	3.5	921470	71473	0	77.3	15.0	16.4	37.6	21.3	39.7			92.8	all but unassigned
avg/sum	3.2	1014035	71473	0	80.1	13.2	14.5	45.4	18.7	34.9			93.4	all with unassigned

taxator-tk binning for FAMeS SimHC

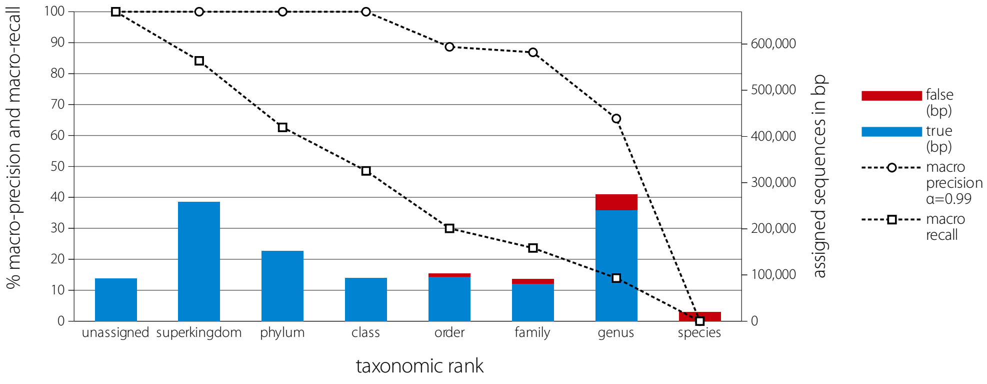

Supplementary Figure S20 - Taxator-tk binning for FAMeS SimHC **(d) new genus scenario**

rank	depth	true (bp)	false (bp)	unknown (bp)	macro precision α=0.99	stdev	pred. bins	macro recall	stdev	real bins	sum true (bp)	sum false (bp)	overall prec.	description
unassigned	0	121975		0	100.0	0.0	1	100.0	0.0	1	804007	0	100.0	root+superkingdom
superkingdom	1	341016	(bp)	0	100.0	0.0	2	82.7	4.9	2				
phylum	2	232318	0	0	100.0	0.0	7	57.5	14.0	8				
class	3	148392	5857	0	98.6	2.6	11	37.0	20.9	12	468720	17552	96.4	phylum+class+order
order	4	88010	11695	0	77.2	38.5	23	17.8	24.3	36				
family	5	69661	14936	0	58.7	46.8	20	8.1	19.0	52				
genus	6	0	39229	0	0.0	0.0	14	0.0	0.0	72	69661	66584	51.1	family+genus+species
species	7	0	12419	0	0.0	0.0	7	0.0	0.0	96				
avg/sum	2.4	879397	84136	0	62.1	12.6	12.0	29.0	11.9	39.7			91.3	all but unassigned
avg/sum	2.2	1001372	84136	0	66.8	11.0	10.6	37.9	10.4	34.9			92.2	all with unassigned

taxator-tk binning for FAMeS SimHC

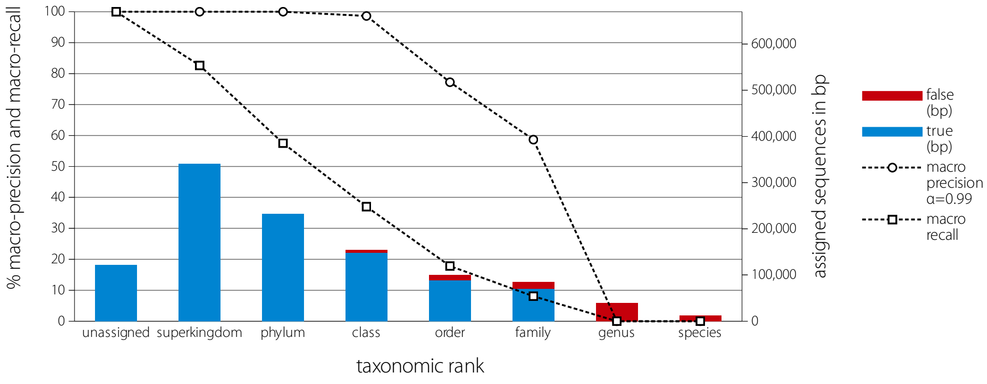

Supplementary Figure S20 - Taxator-tk binning for FAMeS SimHC

(e) new family scenario

rank	depth	true (bp)	false (bp)	unknown (bp)	macro precision α=0.99	stdev	pred. bins	macro recall	stdev	real bins	sum true (bp)	sum false (bp)	overall prec.	description
unassigned	0	243406		0	100.0	0.0	1	100.0	0.0	1	1010134	0	100.0	root+superkingdom
superkingdom	1	383364	(bp)	0	99.6	0.0	1	55.2	21.8	2				
phylum	2	215052	1898	0	98.5	1.9	7	28.4	17.2	8				
class	3	139146	11207	0	92.4	11.7	10	20.6	14.9	12	400621	21498	94.9	phylum+class+order
order	4	46423	8393	0	57.8	44.5	12	3.6	9.5	36				
family	5	0	19955	0	0.0	0.0	11	0.0	0.0	52				
genus	6	0	7389	0	0.0	0.0	6	0.0	0.0	72	0	34843	0.0	family+genus+species
species	7	0	7499	0	0.0	0.0	3	0.0	0.0	96				
avg/sum	2.0	783985	56341	0	49.8	8.3	7.1	15.4	9.1	39.7			93.3	all but unassigned
avg/sum	1.5	1027391	56341	0	56.0	7.3	6.4	26.0	7.9	34.9			94.8	all with unassigned

taxator-tk binning for FAMeS SimHC

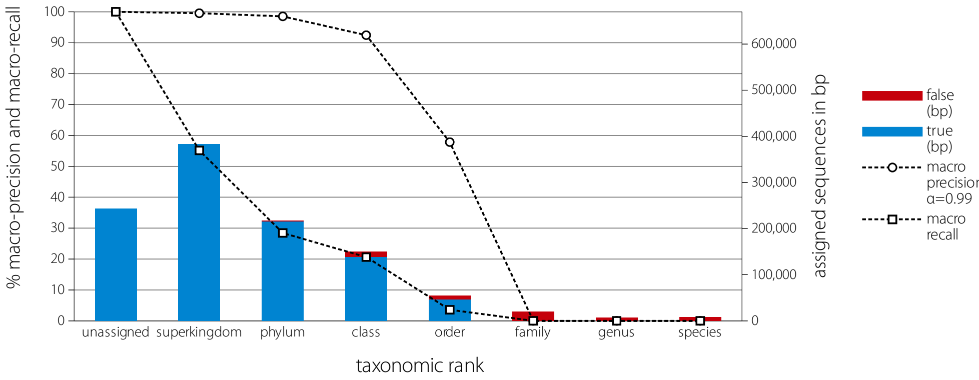

Supplementary Figure S20 - Taxator-tk binning for FAMeS SimHC

(f) new order scenario

rank	depth	true (bp)	false (bp)	unknown (bp)	macro precision α=0.99	stdev	pred. bins	macro recall	stdev	real bins	sum true (bp)	sum false (bp)	overall prec.	description
unassigned	0	197736		0	100.0	0.0	1	100.0	0.0	1	1157742	0	100.0	root+superkingdom
superkingdom	1	480003	(bp)	0	99.6	0.0	1	56.7	23.4	2				
phylum	2	238652	3399	0	91.7	12.7	5	21.9	17.7	8				
class	3	76732	11575	0	70.7	25.1	7	5.3	5.9	12	315384	53711	85.4	phylum+class+order
order	4	0	38737	0	0.0	0.0	13	0.0	0.0	36				
family	5	0	16635	0	0.0	0.0	9	0.0	0.0	52				
genus	6	0	11103	0	0.0	0.0	6	0.0	0.0	72	0	36898	0.0	family+genus+species
species	7	0	9160	0	0.0	0.0	3	0.0	0.0	96				
avg/sum	1.7	795387	90609	0	37.4	5.4	6.3	12.0	6.7	39.7			89.8	all but unassigned
avg/sum	1.4	993123	90609	0	45.2	4.7	5.6	23.0	5.9	34.9			91.6	all with unassigned

taxator-tk binning for FAMeS SimHC

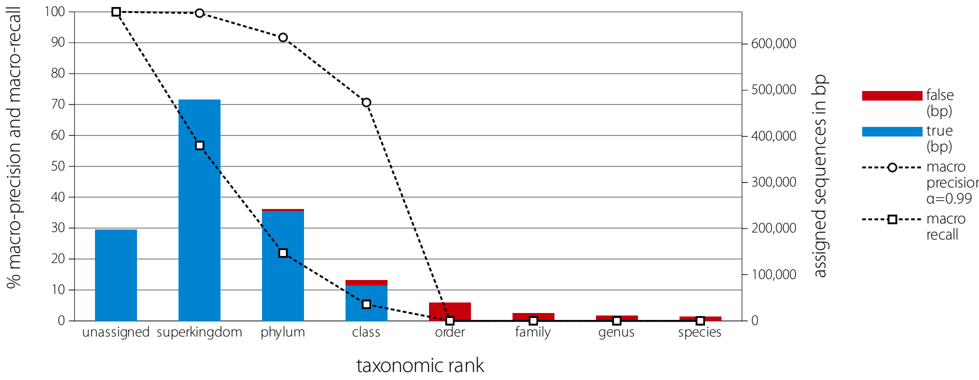

Supplementary Figure S20 - Taxator-tk binning for FAMeS SimHC

(g) new class scenario

rank	depth	true (bp)	false (bp)	unknown (bp)	macro precision α=0.99	stdev	pred. bins	macro recall	stdev	real bins	sum true (bp)	sum false (bp)	overall prec.	description
unassigned	0	366106		0	100.0	0.0	1	100.0	0.0	1	1442954	0	100.0	root+superkingdom
superkingdom	1	538424	(bp)	0	99.5	0.0	1	38.5	27.4	2				
phylum	2	102871	9200	0	42.6	42.8	4	3.8	7.3	8				
class	3	0	39497	0	0.0	0.0	9	0.0	0.0	12	102871	60229	63.1	phylum+class+order
order	4	0	11532	0	0.0	0.0	8	0.0	0.0	36				
family	5	0	7485	0	0.0	0.0	6	0.0	0.0	52				
genus	6	0	3564	0	0.0	0.0	2	0.0	0.0	72	0	16102	0.0	family+genus+species
species	7	0	5053	0	0.0	0.0	2	0.0	0.0	96				
avg/sum	1.4	641295	76331	0	20.3	6.1	4.6	6.0	5.0	39.7			89.4	all but unassigned
avg/sum	0.9	1007401	76331	0	30.3	5.3	4.1	17.8	4.3	34.9			93.0	all with unassigned

taxator-tk binning for FAMeS SimHC

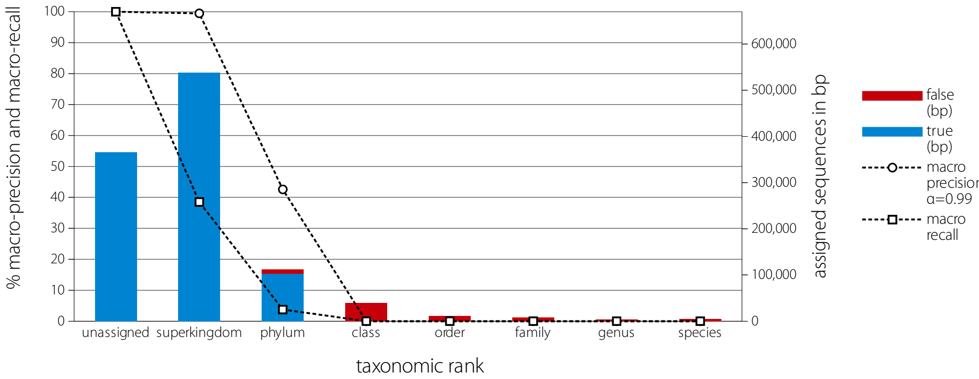

Supplementary Figure S20 - Taxator-tk binning for FAMeS SimHC

(h)new phylum scenario

rank	depth	true (bp)	false (bp)	unknown (bp)	macro precision α=0.99	stdev	pred. bins	macro recall	stdev	real bins	sum true (bp)	sum false (bp)	overall prec.	description
unassigned	0	427371		0	100.0	0.0	1	100.0	0.0	1	1552751	0	100.0	root+superkingdom
superkingdom	1	562690	(bp)	0	99.1	0.0	1	34.1	23.0	2				
phylum	2	0	27047	0	0.0	0.0	11	0.0	0.0	8				
class	3	0	16168	3160	0.0	0.0	11	0.0	0.0	12	0	64740	0.0	phylum+class+order
order	4	0	21525	0	0.0	0.0	10	0.0	0.0	36				
family	5	0	8433	0	0.0	0.0	7	0.0	0.0	52				
genus	6	0	0	0	0.0	0.0	3	0.0	0.0	72	0	21516	0.0	family+genus+species
species	7	0	13083	2676	0.0	0.0	5	0.0	0.0	96				
avg/sum	1.2	562690	86256	5836	14.2	0.0	6.9	4.9	3.3	39.7			86.7	all but unassigned
avg/sum	0.7	990061	86256	5836	24.9	0.0	6.1	16.8	2.9	34.9			92.0	all with unassigned

taxator-tk binning for FAMeS SimHC

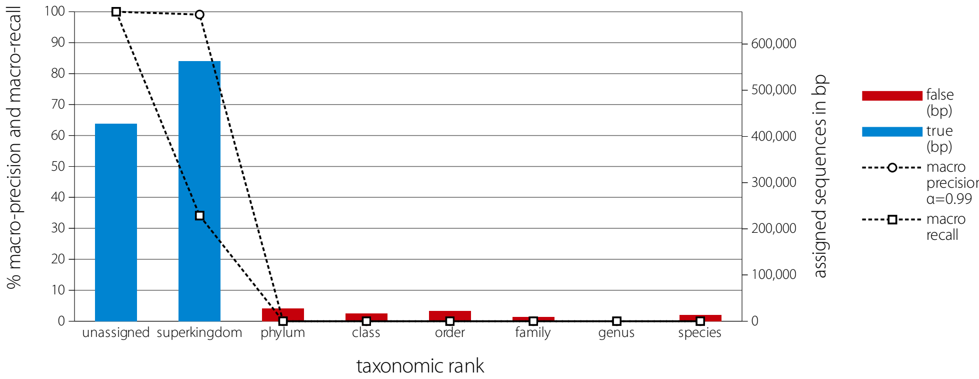

Supplementary Figure S21 - Binning for FAMeS SimMC scenario (Nature Methods 2011) **(a) taxator-tk (nucleotide)**

rank	depth	true (kb)	false (kb)	unknown (kb)	macro precision α=0.95	stdev	pred. bins	macro recall	stdev	real bins	sum true (kb)	sum false (kb)	overall prec.	description
unassigned	0	199.2	0	0	100.0	0.0	1	100.0	0.0	1	9526.04	2.03	100.0	root+superkingdom
superkingdom	1	4663.42	2.03	0	100.0	0.0	1	49.4	49.4	2				
phylum	2	7936.17	2.11	0	100.0	0.0	1	29.9	36.1	8	11881.62	56.97	99.5	phylum+class+order
class	3	2215.89	25.89	0	69.5	42.7	3	18.9	28.7	12				
order	4	1729.56	28.97	0	68.1	45.1	6	20.1	32.0	22				
family	5	191.38	13.42	0	60.1	47.2	14	17.1	33.0	29	212.11	24.95	89.5	family+genus+species
genus	6	19	11.53	0	50.0	50.0	10	9.1	25.3	37				
species	7	1.73	0	0	100.0	0.0	1	2.1	14.4	47				
avg/sum	2.1	16757.15	83.95	0	78.2	26.4	5.1	20.9	31.3	22.4			99.5	all but unassigned
avg/sum	2.1	16956.35	83.95	0	81.0	23.1	4.6	30.8	27.4	19.8			99.5	all with unassigned

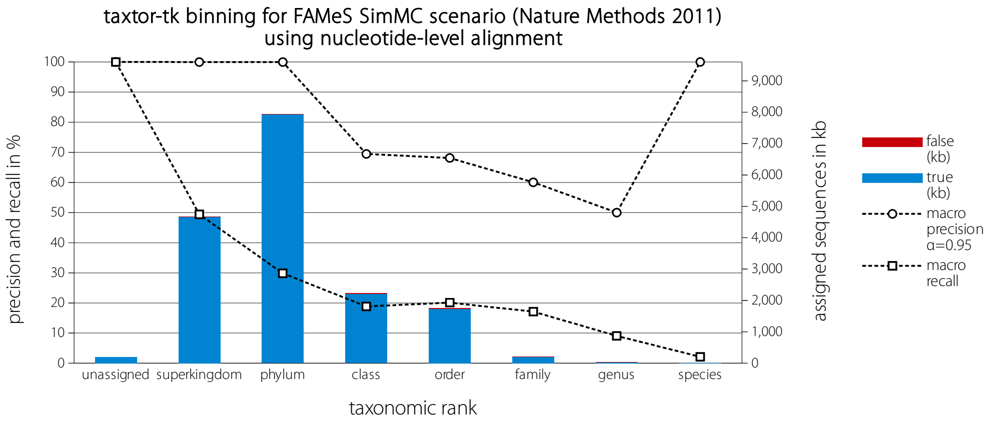

Supplementary Figure S21 - Binning for FAMeS SimMC scenario (Nature Methods 2011) **(b) taxator-tk (amino acid)**

rank	depth	true (kb)	false (kb)	unknown (kb)	macro precision α=0.99	stdev	pred. bins	macro recall	stdev	real bins	sum true (kb)	sum false (kb)	overall prec.	description
unassigned	0	1461.8	0	0	100.0	0.0	1	100.0	0.0	1	9038.52	0	100.0	root+superkingdom
superkingdom	1	3788.36	0	0	100.0	0.0	1	95.7	4.3	2				
phylum	2	4989.57	0	0	100.0	0.0	1	59.9	33.3	8	11175.42	93.58	99.2	phylum+class+order
class	3	3035.46	58.8	0	65.8	46.5	3	27.7	30.1	12				
order	4	3150.39	34.78	0	59.1	44.7	7	22.5	32.4	22				
family	5	347.7	56.72	0	54.6	49.0	18	19.2	35.2	29	377.28	143.85	72.4	family+genus+species
genus	6	17.35	31.58	0	32.1	45.7	18	10.9	28.9	37				
species	7	12.23	55.55	0	29.1	44.0	13	6.4	24.4	47				
avg/sum	2.5	15341.06	237.43	0	63.0	32.8	8.7	34.6	26.9	22.4			98.5	all but unassigned
avg/sum	2.2	16802.86	237.43	0	67.6	28.7	7.8	42.8	23.6	19.8			98.6	all with unassigned

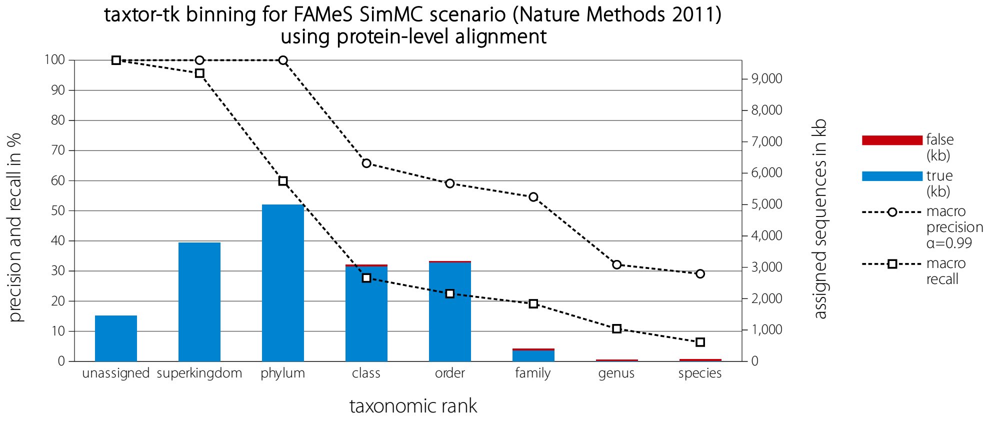

Supplementary Figure S21 - Binning for FAMeS SimMC scenario (Nature Methods 2011) **(c) MEGAN4 (nucleotide)**

rank	depth	true (kb)	false (kb)	unknown (kb)	macro precision α=0.99	stdev	pred. bins	macro recall	stdev	real bins	sum true (kb)	sum false (kb)	overall prec.	description
unassigned	0	1014.03	0	0	100.0	0.0	1	100.0	0.0	1	4082.47	0	100.0	root+superkingdom
superkingdom	1	1534.22	0	0	100.0	0.0	1	47.0	47.0	2				
phylum	2	1896.2	1.83	0	100.0	0.0	1	34.5	38.5	8	4643.84	228.86	95.3	phylum+class+order
class	3	1021.99	108.21	0	65.6	45.6	3	29.4	31.7	12				
order	4	1725.65	118.82	0	29.6	40.6	11	30.1	35.2	22				
family	5	935.47	266.12	0	13.6	32.0	17	15.3	30.8	29	1411.9	8207.44	14.7	family+genus+species
genus	6	18.97	1684.92	0	9.2	25.9	36	3.8	14.5	37				
species	7	457.46	6256.4	0	6.1	21.2	38	0.2	1.0	47				
avg/sum	3.5	7589.96	8436.3	0	46.3	23.6	15.3	22.9	28.4	22.4			47.4	all but unassigned
avg/sum	3.1	8603.99	8436.3	0	53.0	20.7	13.5	32.5	24.8	19.8			50.5	all with unassigned

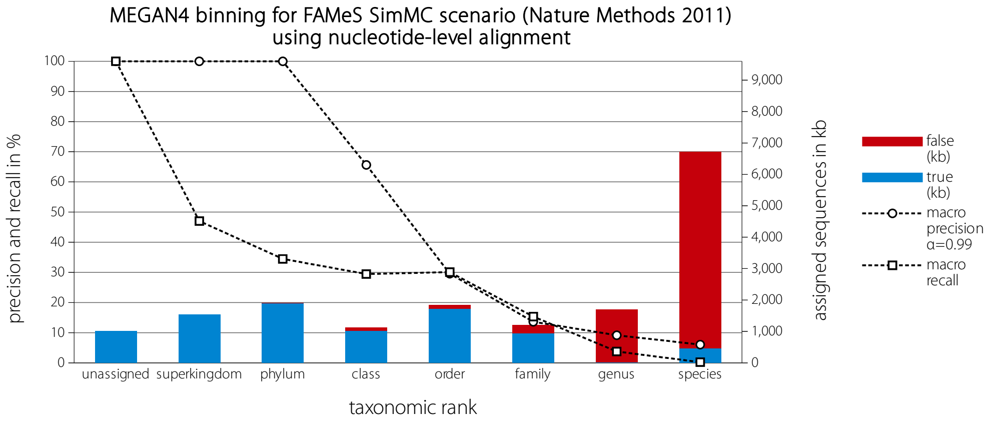

Supplementary Figure S21 - Binning for FAMeS SimMC scenario (Nature Methods 2011) **(d) MEGAN5 (nucleotide)**

rank	depth	true (kb)	false (kb)	unknown (kb)	macro precision α=0.99	stdev	pred. bins	macro recall	stdev	real bins	sum true (kb)	sum false (kb)	overall prec.	description
unassigned	0	1198.19	0	0	100.0	0.0	1	100.0	0.0	1	2696.73	0	100.0	root+superkingdom
superkingdom	1	749.27	0	0	100.0	0.0	1	46.5	46.5	2				
phylum	2	1920.91	19.56	0	50.6	49.3	2	35.1	39.8	8	4741.87	344.53	93.2	phylum+class+order
class	3	1049.16	158.44	0	49.0	47.9	4	27.6	33.1	12				
order	4	1771.8	166.53	0	23.1	38.4	10	25.3	34.2	22				
family	5	961.81	367.01	0	13.5	32.0	17	13.2	29.5	29	1421.6	8584.84	14.2	family+genus+species
genus	6	2.33	1871.14	0	7.5	22.8	32	2.0	10.2	37				
species	7	457.46	6346.69	0	7.4	23.2	31	0.2	1.0	47				
avg/sum	3.7	6912.74	8929.37	0	35.9	30.5	13.9	21.4	27.7	22.4			43.6	all but unassigned
avg/sum	3.3	8110.93	8929.37	0	43.9	26.7	12.3	31.2	24.3	19.8			47.6	all with unassigned

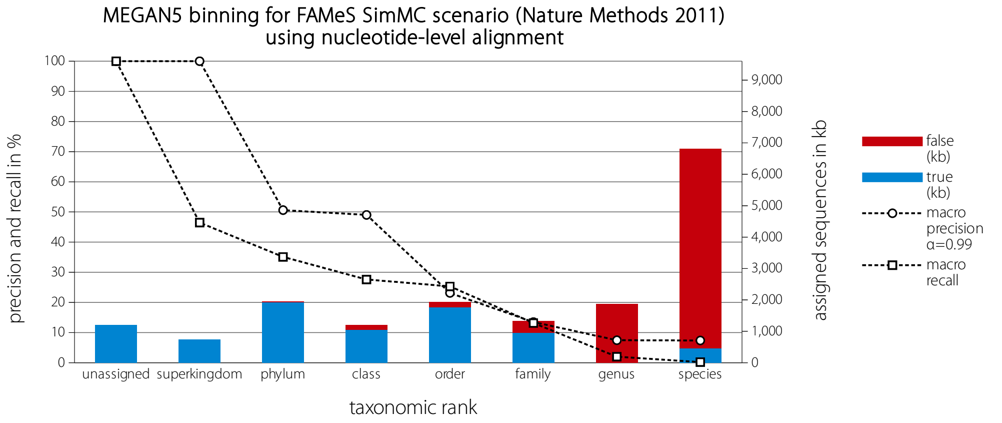

Supplementary Figure S21 - Binning for FAMeS SimMC scenario (Nature Methods 2011) **(e) MEGAN5 (amino acid)**

rank	depth	true (kb)	false (kb)	unknown (kb)	macro precision α=0.99	stdev	pred. bins	macro recall	stdev	real bins	sum true (kb)	sum false (kb)	overall prec.	description
unassigned	0	232.06	0	0	100.0	0.0	1	100.0	0.0	1	3526.24	0	100.0	root+superkingdom
superkingdom	1	1647.09	0	0	100.0	0.0	1	49.3	49.3	2				
phylum	2	3471.29	14	0	51.0	49.0	2	38.7	43.2	8				
class	3	2273.77	182.52	0	49.1	47.6	4	32.8	35.3	12	8695.34	423.54	95.4	phylum+class+order
order	4	2950.28	227.02	0	31.1	37.3	11	25.4	35.4	22				
family	5	1710.01	284.1	0	15.6	32.3	18	7.6	20.1	29				
genus	6	18.97	1024.71	0	12.3	29.1	29	3.8	14.5	37	2114.54	3927.71	35.0	family+genus+species
species	7	385.56	2618.9	0	11.1	30.2	25	0.2	0.8	47				
avg/sum	3.3	12456.97	4351.25	0	38.6	32.2	12.9	22.6	28.4	22.4			74.1	all but unassigned
avg/sum	3.2	12689.03	4351.25	0	46.3	28.2	11.4	32.2	24.8	19.8			74.5	all with unassigned

MEGAN5 binning for FAMeS SimMC scenario (Nature Methods 2011)
using protein-level alignment

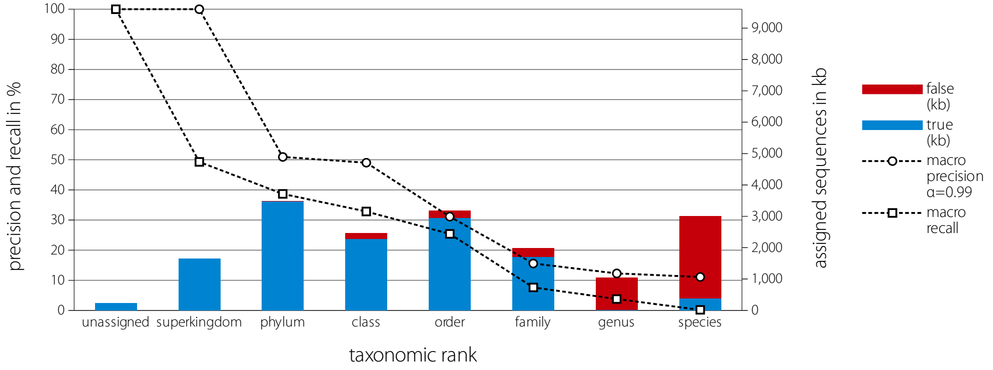

Supplementary Figure S21 - Binning for FAMeS SimMC scenario (Nature Methods 2011) **(f) CARMA (nucleotide)**

rank	depth	true (kb)	false (kb)	unknown (kb)	macro precision α=0.99	stdev	pred. bins	macro recall	stdev	real bins	sum true (kb)	sum false (kb)	overall prec.	description
unassigned	0	1788.99	0	0	100.0	0.0	1	100.0	0.0	1	2125.95	0	100.0	root+superkingdom
superkingdom	1	168.48	0	0	100.0	0.0	1	44.7	44.7	2				
phylum	2	1071.86	49.59	0	48.1	40.4	3	45.9	36.4	8				
class	3	2853.36	446.35	0	38.2	46.0	5	37.3	34.0	12	9749.83	1265.46	88.5	phylum+class+order
order	4	5824.61	769.52	0	23.6	35.1	19	39.7	37.0	22				
family	5	1266.67	1107.7	0	13.7	28.0	50	30.3	41.8	29				
genus	6	364.14	796.45	0	6.8	23.0	93	13.5	32.1	37	1719.92	2347.61	42.3	family+genus+species
species	7	89.11	443.46	0	2.5	15.1	135	2.2	14.4	47				
avg/sum	3.9	11638.23	3613.07	0	33.3	26.8	43.7	30.5	34.4	22.4			76.3	all but unassigned
avg/sum	3.5	13427.22	3613.07	0	41.6	23.4	38.4	39.2	30.1	19.8			78.8	all with unassigned

CARMA binning for FAMeS SimMC scenario (Nature Methods 2011)
using nucleotide-level alignment

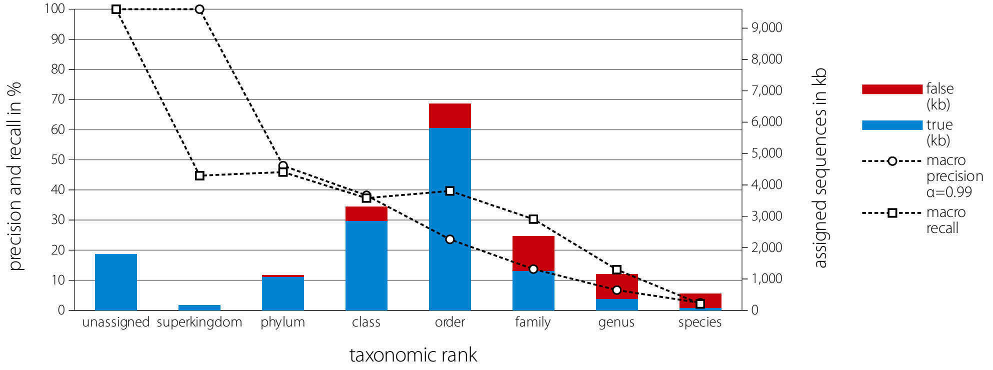

Supplementary Figure S21 - Binning for FAMeS SimMC scenario (Nature Methods 2011) **(g) CARMA (amino acid)**

rank	depth	true (kb)	false (kb)	unknown (kb)	macro precision α=0.99	stdev	pred. bins	macro recall	stdev	real bins	sum true (kb)	sum false (kb)	overall prec.	description
unassigned	0	843.44	0	0	100.0	0.0	1	100.0	0.0	1	3809.88	0	100.0	root+superkingdom
superkingdom	1	1483.22	0	0	100.0	0.0	1	47.5	47.5	2				
phylum	2	3695.28	28.78	0	100.0	0.0	1	46.0	38.1	8				
class	3	3671.01	275.38	0	65.5	44.9	3	35.4	32.6	12	12906.86	538.72	96.0	phylum+class+order
order	4	5540.57	234.56	0	48.0	43.3	10	32.1	33.8	22				
family	5	345.44	315.09	0	28.6	41.3	32	26.3	39.1	29				
genus	6	237.82	174.47	0	21.7	39.9	36	12.4	29.7	37	753.71	514.34	59.4	family+genus+species
species	7	170.45	24.78	0	19.0	38.2	25	4.3	20.2	47				
avg/sum	3.1	15143.79	1053.06	0	54.7	29.7	15.4	29.2	34.4	22.4			93.5	all but unassigned
avg/sum	2.9	15987.23	1053.06	0	60.3	26.0	13.6	38.0	30.1	19.8			93.8	all with unassigned

CARMA binning for FAMeS SimMC scenario (Nature Methods 2011)
using protein-level alignment

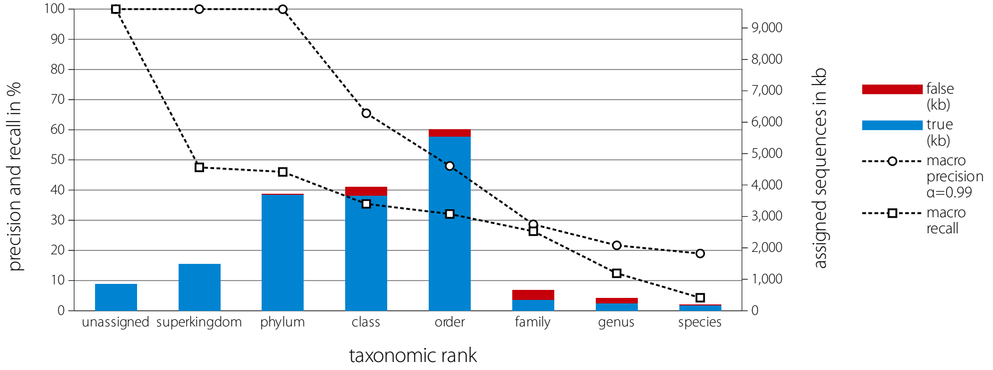

Supplementary Figure S21 - Binning for FAMeS SimMC scenario (Nature Methods 2011) **(h) PhyloPythiaS**

rank	depth	true (kb)	false (kb)	unknown (kb)	macro precision α=0.99	stdev	pred. bins	macro recall	stdev	real bins	sum true (kb)	sum false (kb)	overall prec.	description
unassigned	0	0	0	0	100.0	0.0	1	100.0	0.0	1	1664.72	0	100.0	root+superkingdom
superkingdom	1	832.36	0	0	100.0	0.0	1	49.9	49.9	2				
phylum	2	517.19	25.6	0	100.0	0.0	1	50.8	41.1	8				
class	3	1297.42	52.21	0	67.6	45.5	3	54.5	40.0	12	2474.3	154.19	94.1	phylum+class+order
order	4	659.69	76.38	0	49.6	44.9	6	37.4	34.2	22				
family	5	5715.02	272.58	0	49.3	48.2	6	33.8	40.7	29				
genus	6	7116.9	474.94	0	49.1	46.0	6	23.3	37.4	37	12831.92	747.52	94.5	family+genus+species
species	7													
avg/sum	5.0	16138.58	901.71	0	69.2	30.8	3.8	41.6	40.6	18.3			94.7	all but unassigned
avg/sum	5.0	16138.58	901.71	0	73.6	26.4	3.4	49.9	34.8	15.9			94.7	all with unassigned

PhyloPythiaS binning for FAMeS SimMC scenario (Nature Methods 2011)

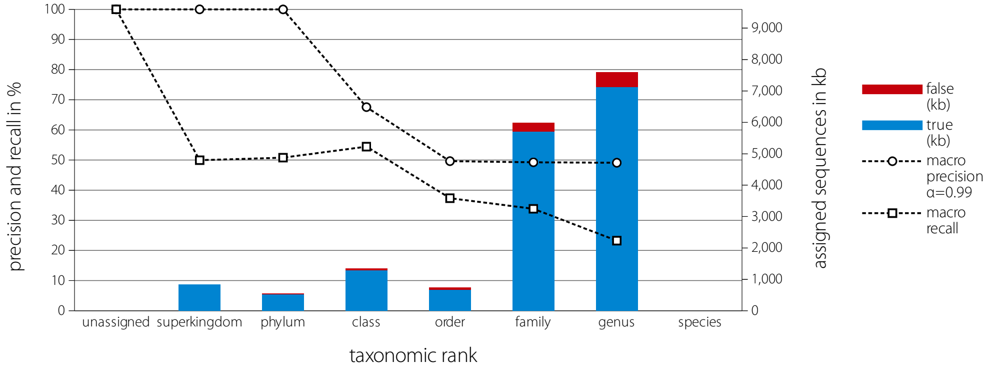

Supplementary Figure S21 - Binning for FAMeS SimMC scenario (Nature Methods 2011) **(i) Kraken**

rank	depth	true (kb)	false (kb)	unknown (kb)	macro precision α=0.99	stdev	pred. bins	macro recall	stdev	real bins	sum true (kb)	sum false (kb)	overall prec.	description
unassigned	0	5974.51	0	0	100.0	0.0	1	100.0	0.0	1	6130.53	0	100.0	root+superkingdom
superkingdom	1	78.01	0	0	100.0	0.0	1	32.5	32.5	2				
phylum	2	208.37	0	0	45.2	41.4	3	32.6	36.0	8	591.64	79.44	88.2	phylum+class+order
class	3	144	17.5	0	36.6	44.9	5	28.2	33.5	12				
order	4	239.27	61.94	0	13.2	28.5	21	36.7	38.6	22				
family	5	78.26	105.58	0	8.2	25.2	43	32.6	42.5	29	606.97	9709.72	5.9	family+genus+species
genus	6	1.01	578.56	0	4.3	17.1	83	20.6	37.9	37				
species	7	527.7	9025.58	0	2.1	13.1	123	11.5	30.9	47				
avg/sum	4.4	1276.62	9789.16	0	29.9	24.3	39.9	27.8	36.0	22.4			11.5	all but unassigned
avg/sum	0.9	7251.13	9789.16	0	38.7	21.3	35.0	36.8	31.5	19.8			42.6	all with unassigned

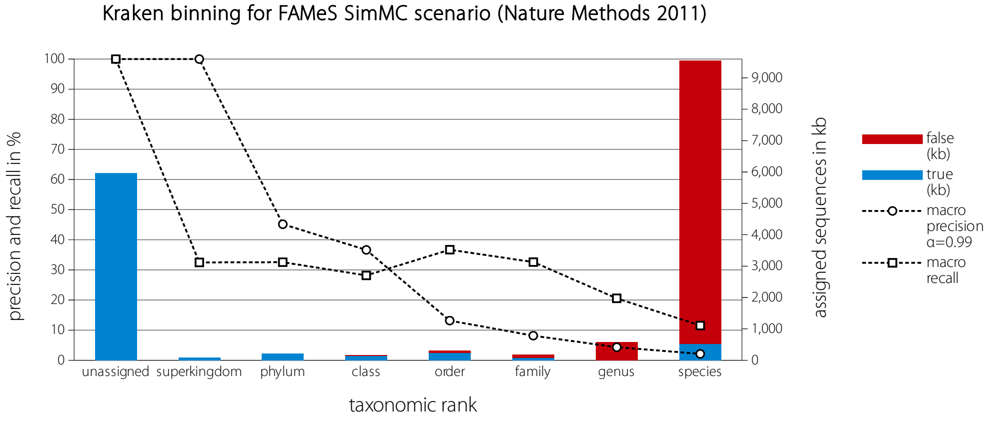

Supplementary Figure S22 - Binning for partitioned cow rumen sample **(a) CARMA (nucleotide)**

rank	depth	consistent (kb)	inconsistent (kb)	unknown (kb)	macro consistency α=0.99	stdev	pred. bins	macro recall	stdev	cons. bins	sum true (kb)	sum false (kb)	overall consist.	description
unassigned	0	144478	0	0	100.0	0.0	1	100.0	0.0	1	350414	154	100.0	root+superkingdom
superkingdom	1	102968	154	0	99.9	0.0	1	42.6	13.9	2				
phylum	2	25730	3872	22	66.6	20.9	13	13.0	5.8	30	41974	7032	85.7	phylum+class+order
class	3	2256	1350	54	61.1	18.7	28	11.1	4.7	52				
order	4	13988	1810	42	55.4	17.1	62	9.7	4.5	99				
family	5	2400	964	104	52.1	23.2	167	9.0	4.8	198	7952	12724	38.5	family+genus+species
genus	6	5552	1090	132	52.6	36.6	572	9.2	5.1	446				
species	7	0	10670	890	0.0	0.0	1254	0.0	0.0	926				
avg/sum	1.8	152894	19910	1244	55.4	16.6	299.6	13.5	5.5	250.4			88.5	all but unassigned
avg/sum	1.0	297372	19910	1244	61.0	14.5	262.3	24.3	4.8	219.3			93.7	all with unassigned

CARMA binning for partitioned cow rumen sample
using nucleotide-level alignment

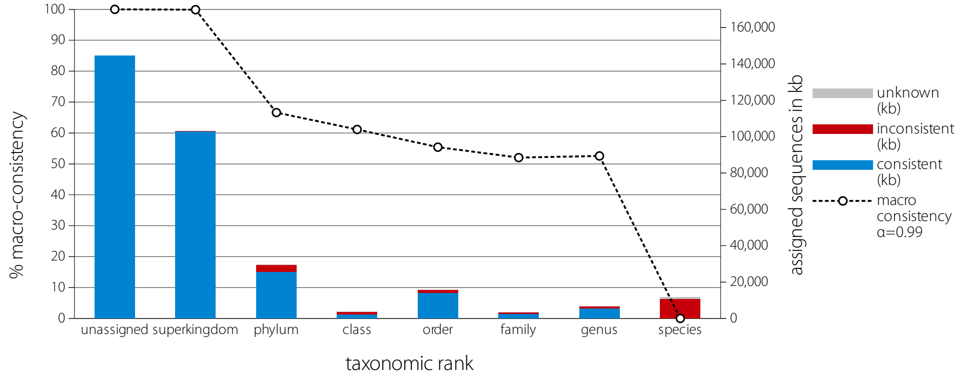

Supplementary Figure S22 - Binning for partitioned cow rumen sample **(b) CARMA (amino acid)**

rank	depth	consistent (kb)	inconsistent (kb)	unknown (kb)	macro consistency α=0.99	stdev	pred. bins	macro recall	stdev	cons. bins	sum true (kb)	sum false (kb)	overall consist.	description
unassigned	0	192034	0	0	100.0	0.0	1	100.0	0.0	1	238074	66	100.0	root+superkingdom
superkingdom	1	23020	66	0	99.9	0.0	1	33.2	7.1	2				
phylum	2	17210	2812	12	47.1	27.8	15	15.4	6.0	30	38912	7660	83.6	phylum+class+order
class	3	2620	1202	6	39.6	21.7	31	13.9	4.5	52				
order	4	19082	3646	14	31.9	17.3	71	12.3	4.3	104				
family	5	2190	2166	82	31.6	14.8	188	11.0	4.1	221	25938	28344	47.8	family+genus+species
genus	6	7412	4932	216	31.1	19.3	611	11.0	4.7	578				
species	7	16336	21246	2222	31.0	29.0	1956	10.4	5.0	1330				
avg/sum	3.0	87870	36070	2552	44.6	18.6	410.4	15.3	5.1	331.0			70.9	all but unassigned
avg/sum	0.9	279904	36070	2552	51.5	16.2	359.3	25.9	4.5	289.8			88.6	all with unassigned

CARMA binning for partitioned cow rumen sample
using protein-level alignment

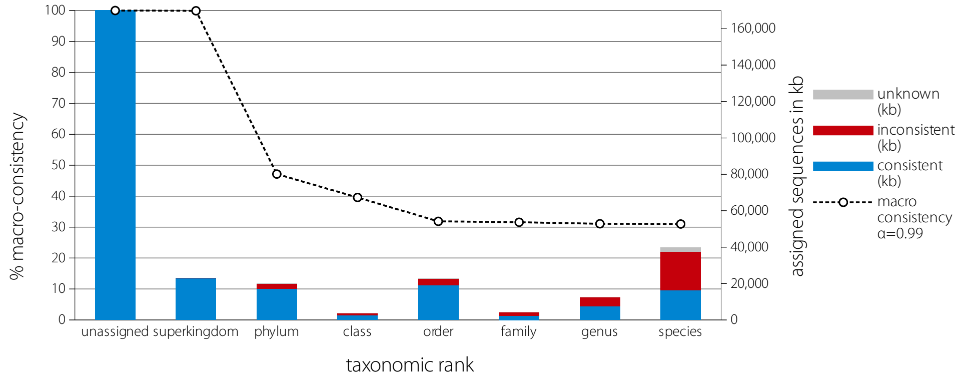

Supplementary Figure S22 - Binning for partitioned cow rumen sample **(c) MEGAN4 (nucleotide)**

rank	depth	consistent (kb)	inconsistent (kb)	unknown (kb)	macro consistency α=0.99	stdev	pred. bins	macro recall	stdev	cons. bins	sum true (kb)	sum false (kb)	overall consist.	description
unassigned	0	87760	0	0	100.0	0.0	1	100.0	0.0	1	218880	116	99.9	root+superkingdom
superkingdom	1	65560	116	0	99.9	0.0	1	43.8	26.2	3				
phylum	2	35352	2802	34	67.4	24.6	12	24.7	16.9	27	79676	7200	91.7	phylum+class+order
class	3	2242	1090	42	51.9	27.7	25	19.7	13.9	48				
order	4	42082	3308	66	39.6	26.3	51	15.5	11.9	88				
family	5	2802	3220	178	34.9	21.1	132	13.6	9.2	168	46888	28304	62.4	family+genus+species
genus	6	12764	6726	436	33.6	20.4	264	12.6	8.0	295				
species	7	31322	18358	2266	38.9	21.9	564	11.7	7.6	535				
avg/sum	2.7	192124	35620	3022	52.3	20.3	149.9	20.2	13.4	166.3			84.4	all but unassigned
avg/sum	1.8	279884	35620	3022	58.3	17.8	131.3	30.2	11.7	145.6			88.7	all with unassigned

MEGAN binning for partitioned cow rumen sample
using nucleotide-level alignment

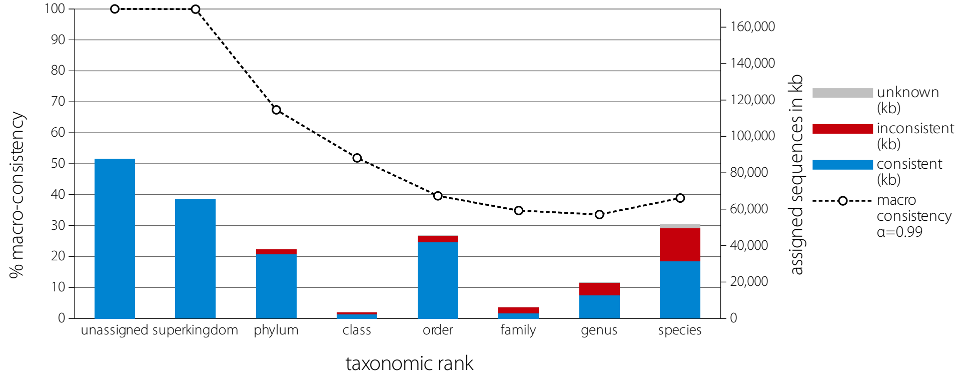

Supplementary Figure S22 - Binning for partitioned cow rumen sample **(d) MEGAN5 (amino acid)**

rank	depth	consistent (kb)	inconsistent (kb)	unknown (kb)	macro consistency α=0.99	stdev	pred. bins	macro recall	stdev	cons. bins	sum true (kb)	sum false (kb)	overall consist.	description
unassigned	0	36062	0	0	100.0	0.0	1	100.0	0.0	1	217142	220	99.9	root+superkingdom
superkingdom	1	90540	220	0	99.9	0.0	1	72.9	15.9	2				
phylum	2	59686	4074	26	67.2	25.6	12	25.3	17.8	26	112124	10496	91.4	phylum+class+order
class	3	6054	1614	24	51.0	27.9	25	20.1	13.4	44				
order	4	46384	4808	118	36.3	27.1	52	15.9	11.4	79				
family	5	4208	4130	358	35.1	21.7	119	13.1	8.7	140	41984	23208	64.4	family+genus+species
genus	6	13258	6756	778	36.7	20.3	203	12.3	7.9	218				
species	7	24518	12322	2588	46.2	20.5	347	11.5	8.6	343				
avg/sum	2.5	244648	33924	3892	53.2	20.4	108.4	24.4	11.9	121.7			87.8	all but unassigned
avg/sum	2.2	280710	33924	3892	59.1	17.9	95.0	33.9	10.5	106.6			89.2	all with unassigned

MEGAN binning for partitioned cow rumen sample
using protein-level alignment

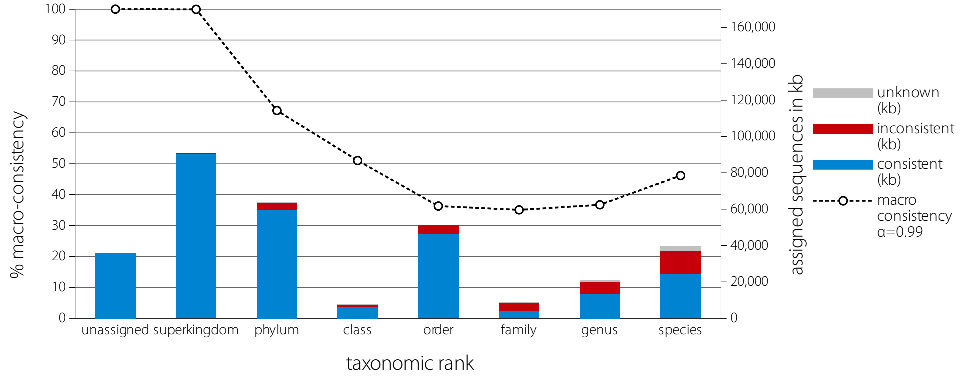

Supplementary Figure S22 - Binning for partitioned cow rumen sample **(e) taxator-tk (nucleotide)**

rank	depth	consistent (kb)	inconsistent (kb)	unknown (kb)	macro consistency α=0.99	stdev	pred. bins	macro recall	stdev	cons. bins	sum true (kb)	sum false (kb)	overall consist.	description
unassigned	0	122146	0	0	100.0	0.0	1	100.0	0.0	1	353002	62	100.0	root+superkingdom
superkingdom	1	115428	62	0	100.0	0.0	1	56.8	7.3	2				
phylum	2	39828	1152	4	87.7	16.9	7	16.4	12.3	22				
class	3	1676	334	28	80.2	17.0	14	13.7	11.3	34	65086	2212	96.7	phylum+class+order
order	4	23582	726	28	78.3	20.2	16	11.7	10.8	56				
family	5	2524	198	100	79.3	19.8	50	10.3	8.8	84				
genus	6	8938	198	94	76.2	35.9	110	9.8	7.8	94	12810	440	96.7	family+genus+species
species	7	1348	44	88	78.0	37.4	123	8.6	6.7	103				
avg/sum	1.9	193324	2714	342	82.8	21.0	45.9	18.2	9.3	56.4			98.6	all but unassigned
avg/sum	1.2	315470	2714	342	85.0	18.4	40.3	28.4	8.1	49.5			99.1	all with unassigned

taxator-tk binning for partitioned cow rumen sample
using nucleotide-level alignment

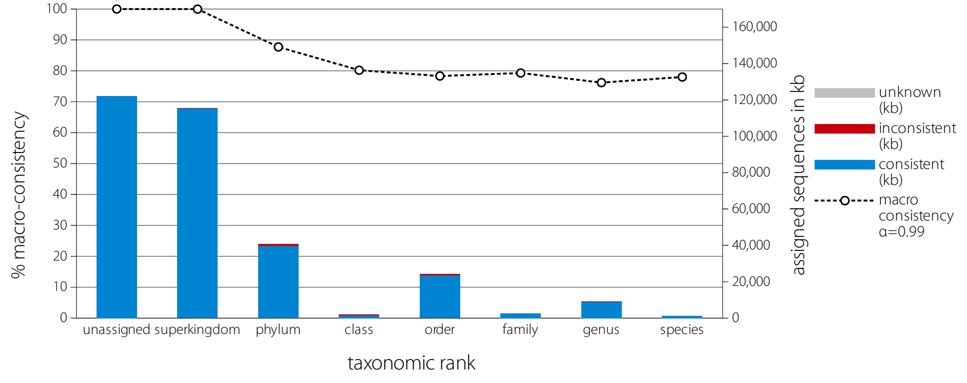

Supplementary Figure S22 - Binning for partitioned cow rumen sample **(f) taxator-tk (amino acid)**

rank	depth	consistent (kb)	inconsistent (kb)	unknown (kb)	macro consistency α=0.99	stdev	pred. bins	macro recall	stdev	cons. bins	sum true (kb)	sum false (kb)	overall consist.	description
unassigned	0	36398	0	0	100.0	0.0	1	100.0	0.0	1	361102	144	100.0	root+superkingdom
superkingdom	1	162352	144	0	100.0	0.0	1	74.1	14.5	2				
phylum	2	63942	1604	2	94.7	6.2	5	20.9	15.7	17				
class	3	5920	410	24	88.6	14.1	10	16.3	13.1	26	104336	2614	97.6	phylum+class+order
order	4	34474	600	30	89.3	15.4	9	14.0	12.4	37				
family	5	2586	92	72	78.6	22.0	32	11.8	9.4	51				
genus	6	8344	228	82	81.2	26.8	27	10.5	8.1	55	12066	328	97.4	family+genus+species
species	7	1136	8	78	78.7	37.7	59	9.8	7.8	49				
avg/sum	1.8	278754	3086	288	87.3	17.5	20.4	22.5	11.6	33.9			98.9	all but unassigned
avg/sum	1.6	315152	3086	288	88.9	15.3	18.0	32.2	10.1	29.8			99.0	all with unassigned

taxator-tk binning for partitioned cow rumen sample
using protein-level alignment

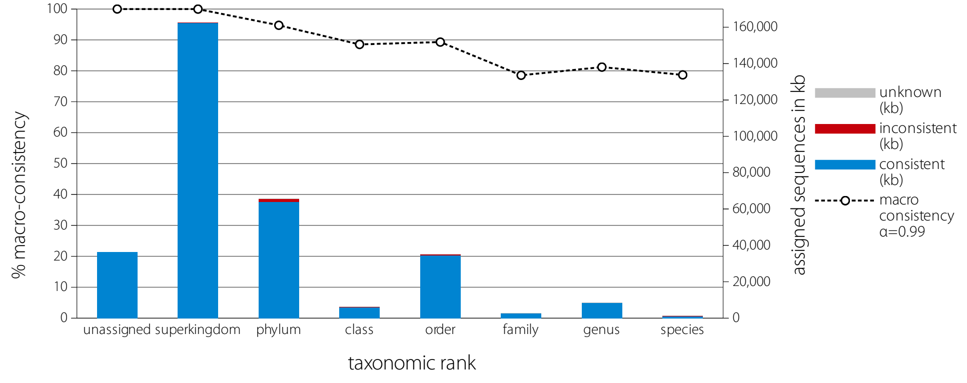

Supplementary Figure S22 - Binning for partitioned cow rumen sample **(g) PhyloPythiaS**

rank	depth	consistent (kb)	inconsistent (kb)	unknown (kb)	macro consistency α=0.99	stdev	pred. bins	macro recall	stdev	cons. bins	sum true (kb)	sum false (kb)	overall consist.	description
unassigned	0	0	0	0	100.0	0.0	1	100.0	0.0	1	296276	2810	99.1	root+superkingdom
superkingdom	1	148138	2810	0	100.0	0.0	1	81.6	17.5	2				
phylum	2	65136	24220	4	66.6	15.1	4	31.0	14.7	7				
class	3	9468	9930	568	55.3	22.0	10	21.0	10.7	13	106338	40640	72.3	phylum+class+order
order	4	31734	6490	828	56.2	21.0	19	12.8	4.9	25				
family	5	3990	1438	708	58.7	18.9	30	10.4	4.1	39				
genus	6	6144	1078	1072	62.8	18.5	33	9.2	4.2	45	13842	3054	81.9	family+genus+species
species	7	3708	538	524	64.7	29.0	64	8.0	4.6	67				
avg/sum	2.0	268318	46504	3704	66.3	17.8	23.0	24.9	8.7	28.3			85.2	all but unassigned
avg/sum	2.0	268318	46504	3704	70.6	15.6	20.3	34.3	7.6	24.9			85.2	all with unassigned

PhyloPythiaS binning for partitioned cow rumen sample

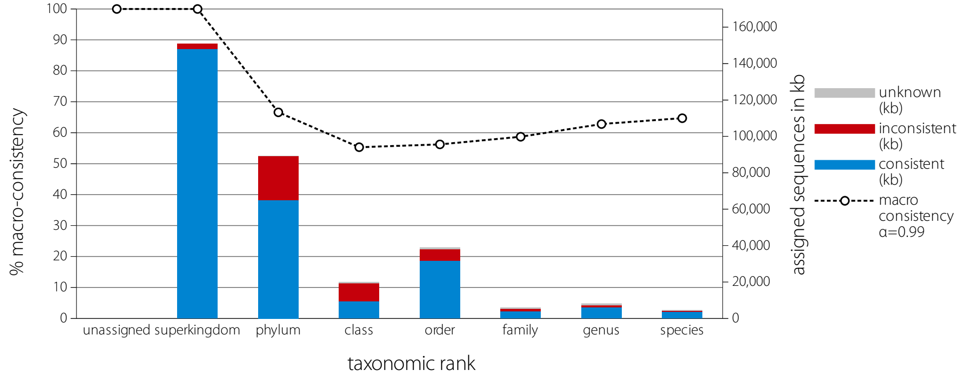

Supplementary Figure S22 - Binning for partitioned cow rumen sample **(h) Kraken**

rank	depth	consistent (kb)	inconsistent (kb)	unknown (kb)	macro consistency α=0.99	stdev	pred. bins	macro recall	stdev	cons. bins	sum true (kb)	sum false (kb)	overall consist.	description
unassigned	0	265616	0	0	100.0	0.0	1	100.0	0.0	1	270140	4	100.0	root+superkingdom
superkingdom	1	2262	4	0	99.9	0.0	1	21.0	1.2	2				
phylum	2	728	504	0	42.6	25.6	18	10.8	5.8	30				
class	3	190	434	2	41.3	22.9	33	10.5	5.3	52	2034	1628	55.5	phylum+class+order
order	4	1116	690	8	34.5	18.4	77	9.9	5.0	110				
family	5	254	684	8	33.5	15.3	195	9.3	4.3	233				
genus	6	1378	2736	34	32.4	19.5	661	9.7	4.6	640	19274	27218	41.5	family+genus+species
species	7	17642	23798	438	33.3	28.2	1953	9.9	5.2	1461				
avg/sum	3.9	23570	28850	490	45.4	18.6	419.7	11.6	4.5	361.1			45.0	all but unassigned
avg/sum	0.2	289186	28850	490	52.2	16.2	367.4	22.6	3.9	316.1			90.9	all with unassigned

Kraken binning for partitioned cow rumen sample

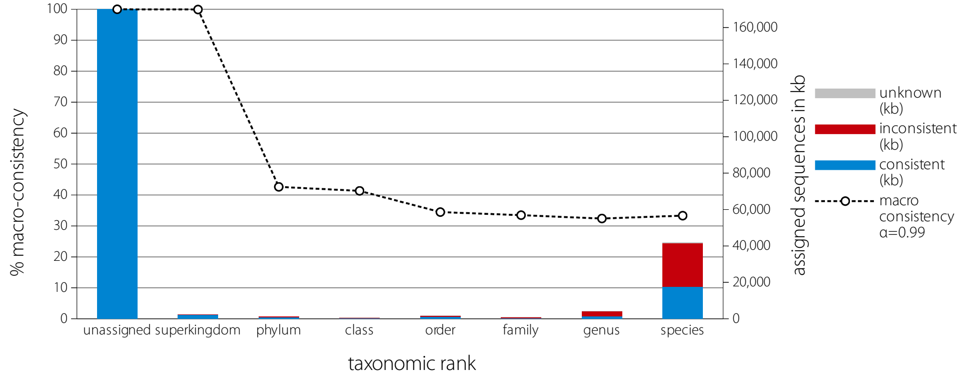

Supplementary Figure S23: Parallel speedup of program *taxator*

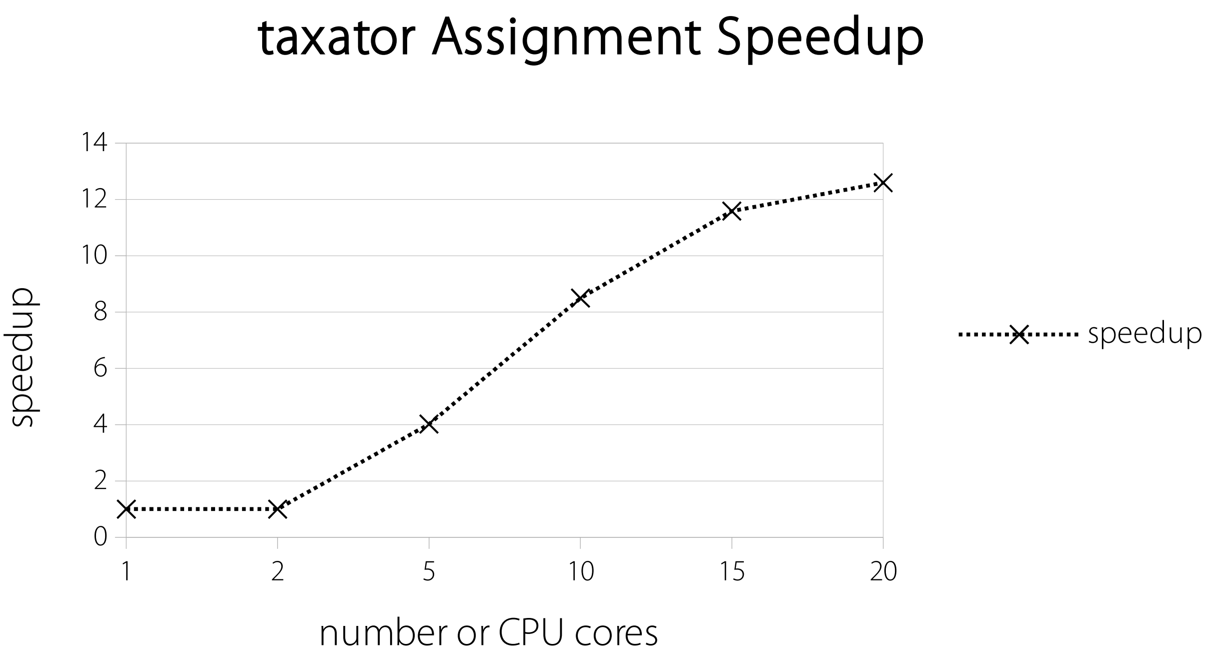

Execution time analysis with taxator for parallelized processing with multiple CPU cores. Taxonomic placement of sequence segments with taxator on input alignments for sequences of length 1000 bp (*syn1000* data-set aligned against *mRefSeq47* with *LAST*). The speedup was calculated using wall clock time for a parallelized run relative to serial execution with one CPU thread. With multiple threads, there is always one producer thread (consumer-producer model). Thus for more than two threads, multiple consumers work on the input data in parallel. An approximate linear scale-up was observed up to 15 threads and saturation effects appear when using 20 CPU cores on our system.

$$speedup = \frac{T_1}{T_p}, \textit{ with}$$

T_1: *serial execution time*

T_p: *execution time using p threads and CPU cores*

Supplementary Figure S24: Effect of input sequence length and segmentation on *taxator-tk* processing time.

(a)

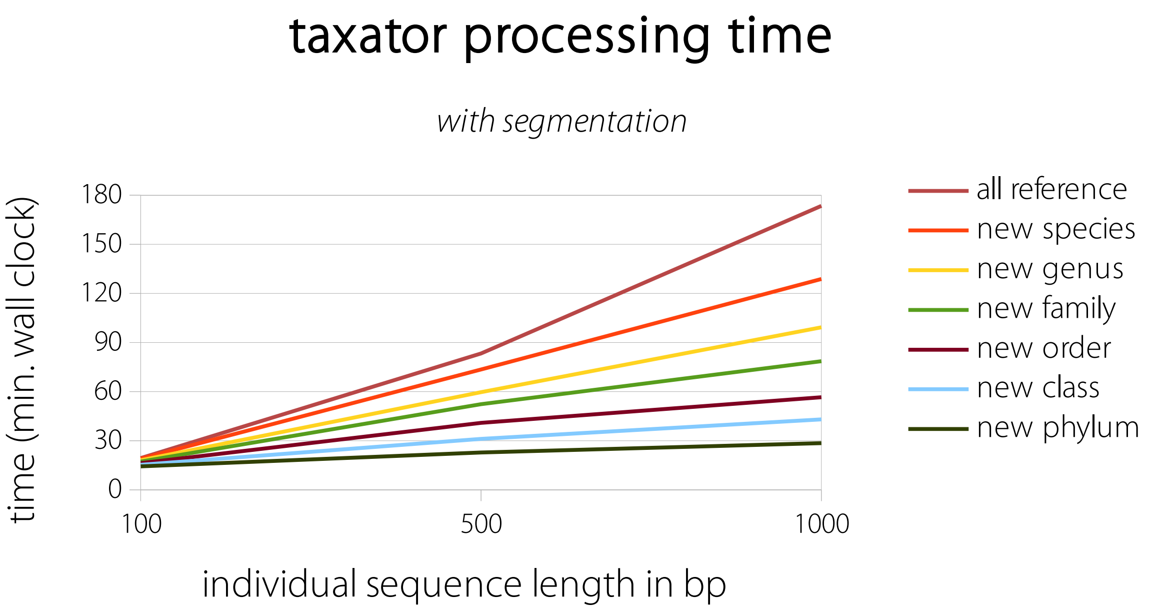

(b)

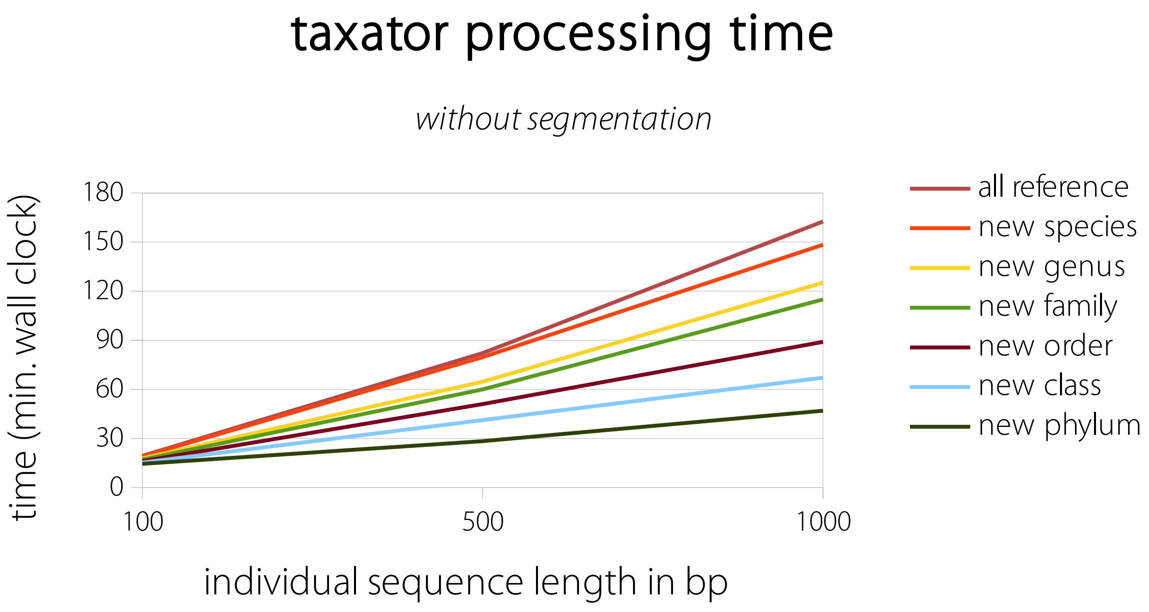

We processed approximately the same number of sequences of length 100, 500 and 1000 bp with *taxator-tk* (*syn100*,*syn500*,*syn1000*), once with the segmentation procedure being enabled (a) and once with segmentation disabled (b). The run-time increases for both cases are approximately linear with the input length, where the slope depends on the completeness of the reference sequence data. With all reference data available, the run-time increases more than linear, as there is no segmentation of queries during computations. For all other cases, segmentation substantially decreases the execution time.

Supplementary Figure S25: Example GFF3 output of taxator

```
##gff-version 3
contig_0 taxator-tk sequence_feature 102   121  1     .      .     seqlen=1012;tax=1224:19;ival=0.5
contig_0 taxator-tk sequence_feature 155   194  0.91  .      .     seqlen=1012;tax=2:32-1;ival=0.8
contig_0 taxator-tk sequence_feature 201   220  1     .      .     seqlen=1012;tax=40324:20-2;ival=0
contig_0 taxator-tk sequence_feature 225   243  1     .      .     seqlen=1012;tax=316277:19-1;ival=1
contig_0 taxator-tk sequence_feature 246   301  1     .      .     seqlen=1012;tax=731:38-1224;ival=0.72
contig_0 taxator-tk sequence_feature 326   471  1     .      .     seqlen=1012;tax=338:87-1224;ival=0.98
contig_0 taxator-tk sequence_feature 486   554  0.60  .      .     seqlen=1012;tax=1224:59-2;ival=0.67
contig_0 taxator-tk sequence_feature 555   616  0.63  .      .     seqlen=1012;tax=32008:43-1224;ival=0.86
contig_0 taxator-tk sequence_feature 633   651  1     .      .     seqlen=1012;tax=876:19-1;ival=1
contig_0 taxator-tk sequence_feature 670   745  0.89  .      .     seqlen=1012;tax=31998:60-1;ival=0.89
contig_0 taxator-tk sequence_feature 786   809  0.89  .      .     seqlen=1012;tax=256618:23-2;ival=0.2
contig_0 taxator-tk sequence_feature 886   932  1     .      .     seqlen=1012;tax=644:33-2;ival=0.67
contig_0 taxator-tk sequence_feature 958   980  1     .      .     seqlen=1012;tax=347:22-1;ival=1
```

Query identifier	Generator	Type	Begin	End	Score	Strand	Phase	Query length<br>Taxonomic range and support<br>Interpolation value

Query segment assignments calculated by the program *taxator* (version 1.1.1) are generated in standard GFF3 format. Each tab-separated field holds the information which is named in the bottom description. The score measures the assignment quality and is under ongoing improvement. Strand and phase contain a dot as placeholder as they are invalid GFF3 fields for this output. The last column holds data in a key-value scheme and includes the query sequence length, a taxonomic prediction range of the form low:support-high where low/specific (node X in Fig. 2a) and high/general (node R in Fig. 2a) are NCBI taxon IDs. The included interpolation value ranging from zero (low) and one (high) can be used to determine an approximate position in the given taxonomic range. As it might become necessary for post-processing applications such as whole sequence binning, more information can be added in the last column while preserving backward compatibility.
